# Supramolecular self-associating amphiphiles (SSAs) as enhancers of antimicrobial agents towards *Escherichia coli* (*E. coli*)†

**DOI:** 10.1039/d1ra00998b

**Published:** 2021-03-03

**Authors:** Jessica E. Boles, Rebecca J. Ellaby, Helena J. Shepherd, Jennifer R. Hiscock

**Affiliations:** School of Physical Sciences, University of Kent Canterbury CT2 7NH UK J.R.Hiscock@Kent.ac.uk

## Abstract

Supramolecular self-associating amphiphiles (SSAs) are a class of amphiphilic salt which have demonstrated antimicrobial activity against both Gram-positive and Gram-negative bacteria. Herein, we show that SSAs are also able to increase the efficacy of a range of currently used antimicrobial/therapeutic agents with a range of different chemical structures and modes of antimicrobial action against Gram-negative *Escherichia coli*, which include: octenidine (an antiseptic); ampicillin (an antibiotic); and cisplatin (a DNA chelating agent). Additionally, we show these effects to be dependent on the order of agent addition. Finally, through completion of a range of 1 : 1 SSA :  antimicrobial/therapeutic agent physicochemical studies we gain an understanding as to how the self-association events and resultant SSA aggregate structure are effected by the presence of these secondary molecular species.

## Abstract 2

Using supramolecular self-associating amphiphiles (SSAs) and antimicrobial/therapeutic agents as combination therapies that lead to increased efficacy against *Escherichia coli*.

## Graphical abstract

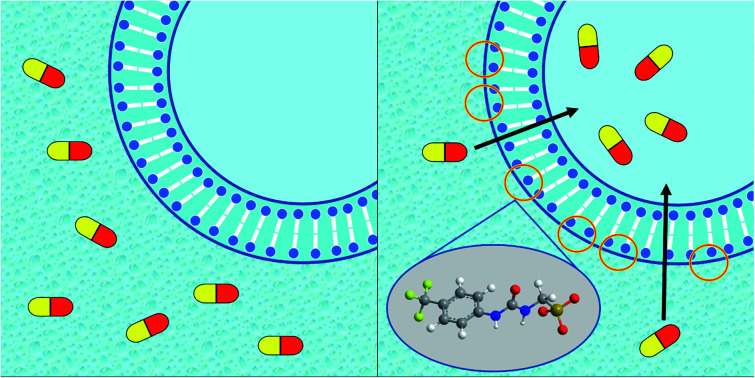

## Introduction

The discovery of antibiotics/antimicrobials has revolutionised healthcare, with these agents becoming a game changing weapon in the fight against bacterial infections.^1^ However, since the first legitimate use of these agents, bacteria have evolved resistance. The ongoing misuse of these same compounds within the clinical,^2^ veterinary^3^ and animal feedstuff sectors,^4^ has resulted in the development of widespread microbial resistance and the emergence of multi-drug-resistant bacterial strains.^5^ To date, antimicrobial resistance (AMR) has now been reported towards all antimicrobial agents currently available,^6^ including antiseptics such as octenidine^6^ and more ominously, the antibiotic of last resort, colistin.^7^ Additionally, a recently commissioned UK governmental report has predicted that by 2050, approximately 10 million people per year will die globally from the primary effects of AMR, overtaking those caused by cancer in 2014, *ca.* 8.2 million per year.^8^

In light of the growing prevalence of AMR, the development of new molecular weapons to combat the threat of bacterial infection are of increasing importance.^5^ The development of such technologies include those driven by the supramolecular chemistry community.^9^ Developments in this area include work by Gunnlaugsson *et al.* who have used the aryl-pyridyl urea scaffold within supramolecular gelator design to produce a material which demonstrates antimicrobial activity against both Gram-positive methicillin resistant *Staphylococcus aureus* (MRSA) and Gram-negative *Escherichia coli* (*E. coli*).^10^ In addition, Hou *et al.* have demonstrated the ability of pillar[5]arenes to selectively insert into Gram-positive bacterial membranes, exhibiting efficient antimicrobial activity against *Staphylococcus epidermidis*.^11^ Furthermore, the supramolecular silica nanoplatform developed by Zink *et al.* has been shown to co-deliver the antibiotic ofloxacin and the antimicrobial peptide melittin for synergistic eradication of *Pseudomonas aeruginosa* PAO1 biofilms.^12^ Finally, Zhang *et al.* have demonstrated the enhanced antibacterial activity of silver nitrate when combined with PEGylated bisimidazolylbenzyl alcohol, against both MRSA and *E. coli*.^13^

**Fig. 1:**
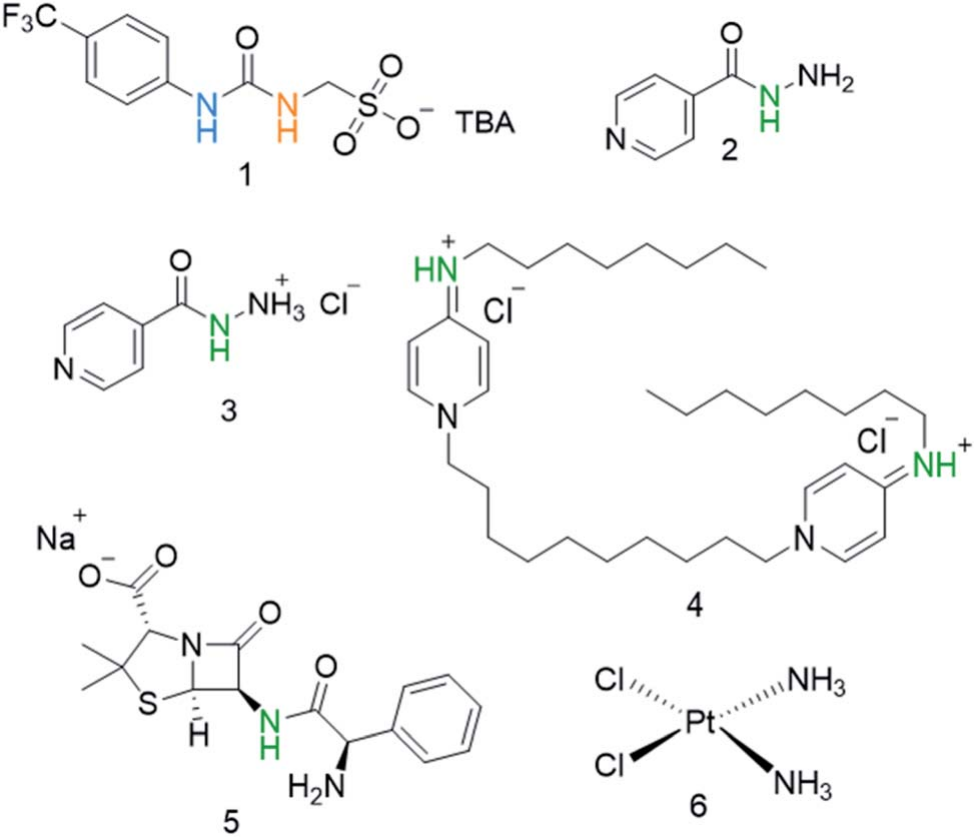
Chemical structures of SSA 1 and antimicrobial/therapeutic co-formulants 2–6. TBA = tetrabutylammonium. The NHs highlighted in green, blue and orange indicate the resonances monitored during ^1^H NMR dilution studies (Fig. 3).

Our own work in this area has focused on the development of a novel class of supramolecular, self-associating amphiphilic salts (SSAs), such as those exemplified by 1 (Fig. 1).^14–21^ Similar constructs developed by Faustino and co-workers have previously been shown to act as amphiphiles with enhanced surfactant properties, attributed to intermolecular hydrogen bonded self-association.^22,23^ Additionally, Supuran *et al.* have shown the promise of agents such as SSAs for development as therapeutics against *Mycobacterium tuberculosis* (TB).^24^ To date, members from this >70 compound SSA library have been shown to self-associate within the solution state, producing self-associated dimers, spherical aggregates and hydrogels.^16^ Additionally, SSAs have also demonstrated antimicrobial activity against Gram-positive MRSA and Gram-negative *E. coli*.^14–16,20^ This previous work has led us to hypothesise that SSA antimicrobial activity is linked to both molecular self-association and selective SSA:phospholipid complexation, resulting in molecular membrane permeation events.^15,16,25,26^ Additionally, not only have we shown SSAs to arrive at the external microbial membrane as self-associated spherical aggregates,^16^ but that these aggregates can incorporate small drug(like) guest species.^17^ The summation of this evidence has therefore led us to hypothesise that this class of compound has the potential to act as efficacy enhancers for other therapeutic agents. Herein, we characterise the SSA aggregates formed upon the co-formulation of 1 (Fig. 1) with examples of antiseptics, antibiotics, and other antimicrobial/cytotoxic agents (2–6). Additionally, we explore the ability of 1 to enhance the activity of 2–6 against the model Gram-negative bacteria, *E. coli*.

## Results and discussion

### Physicochemical characterisation of SSA co-formulations

A series of SSA co-formulation physicochemical studies, conducted in line with previous work,^17^ allowed us to understand the effects of the addition of 2–6 on the self-associative properties of SSA 1, when supplied in an 1 : 1 molecular ratio. The preparation of these 1 : 1 SSA :  secondary compound mixtures produced co-formulations a–e, as detailed in Table 1. Here, 1 was synthesised in line with previously published procedures,^27^ whereas 2–6 were purchased from commercial sources and used without further purification.

**Table 1:** Molecular components, supplied in a 1 : 1 ratio, for co-formulations a–e

Co-formulation	Molecular components	Co-formulation	Molecular components
a	1 + 2	d	1 + 5
b	1 + 3	e	1 + 6
c	1 + 4		

Due to the complexities associated with SSA self-association events, the physicochemical properties of 1 when co-formulated with 2–6 have been studied through a variety of complimentary methods that include: single crystal X-ray diffraction, quantitative ^1^H NMR, ^1^H NMR self-association constant determination, ^1^H NMR DOSY, dynamic light scattering (DLS), zeta potential studies, surface tension and critical micelle concentration (CMC) determination.† However, we have not attempted scanning/transmission electron microscopy (SEM/TEM) studies, as the self-associated aggregate structures produced by SSAs have previously been shown not to survive traditional SEM/TEM sample preparation methods.^27^

**Fig. 2:**
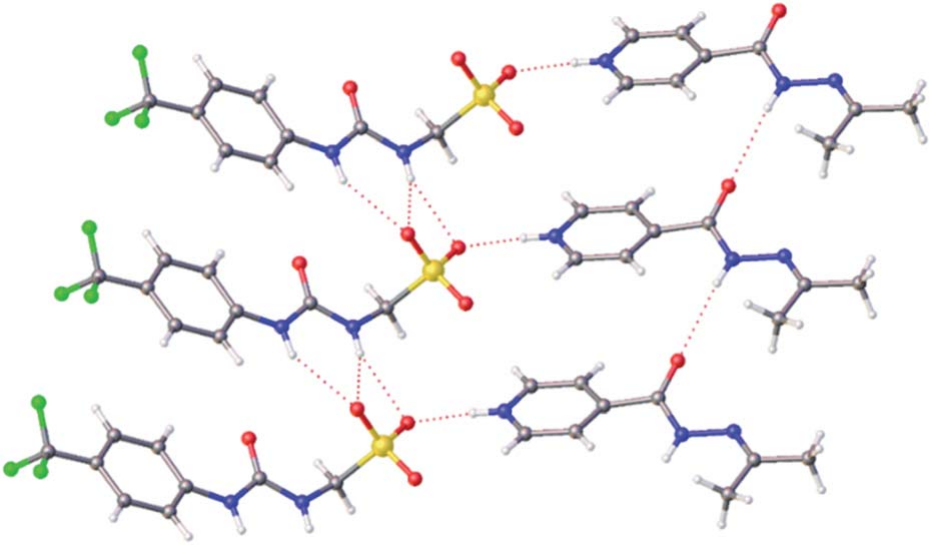
A single crystal X-ray structure obtained from a single crystal sample produced from a solution of co-formulation a in acetone. Grey = carbon, blue = nitrogen, red = oxygen, yellow = sulfur, white = hydrogen, green = fluorine, red dashed lines = hydrogen bonds.‡

Slow evaporation of co-formulation a in acetone led to the generation of a crystal sample, suitable for single crystal X-ray diffraction analysis, Fig. 2.‡‡A suitable crystal was selected and mounted on a Rigaku Oxford Diffraction Supernova diffractometer. Data were collected using Cu Kα radiation at 100 K. The structure was solved with the ShelXS^38^*via* direct methods and refined with ShelXL^39^ on least squares minimisation. Olex2 (ref. 40) was used as an interface to all ShelX programs. CCDC deposition number for the structure shown in Fig. 2 = 1999018. Here 2 has undergone a reaction with the acetone, as previously described by Sarcevica *et al.*^28^ The product of this reaction, the cation shown in Fig. 2, has then formed a salt with the anionic component of 1. Here we see the formation of a hydrogen bonded urea-sulfonate anionic tape, similar to that observed previously for the pyridinium salt of this same anion.^29^ As observed for this analogous structure, the counter cation is also involved in competitive hydrogen bonded complexation events with the anionic sulfonate group, preventing sulfonate-urea dimer formation as observed for 1 only.^29^

SSA self-association events are known to be dependent on solvent environment, typically forming hydrogen bonded anionic dimers in DMSO, larger (≈100–550 nm in diameter) self-associated spherical aggregates in aqueous or aqueous ethanol (19 : 1 H_2_O : EtOH) solutions and self-associated hydrogel fibers in aqueous salt solutions.^16^ Initially, to enable characterisation of those SSA self-associated species present within the solution state, quantitative ^1^H NMR techniques are used to confirm the presence of larger self-associated species. Here comparative integration against an internal standard is used to calculate the proportion of a molecular component visible using standard solution state NMR techniques. The proportion of a molecular component that appears ‘lost’ from a solution is assumed to form larger higher-order self-associated structures with solid-like properties, thus rendering them NMR inactive. These studies are thus also able to enable the elucidation of the proportion of any secondary molecular substituents (such as 2–6) involved in the construction of these larger higher order species.

Those solutions which exhibit no apparent ‘loss’ of molecular components, through comparative integration with the appropriate internal standard, are taken forward for ^1^H NMR self-association constant determination studies. These studies enable us to quantify the strength of the hydrogen-bonded self-association events undertaken by the anionic SSA components, through completion of ^1^H NMR dilution studies and the subsequent fitting of these data and generation of self-association constants, using BindFit v0.5.^30^ The limitation of this method however, is that these models are limited to fitting one component, one dimensional, homogeneous aggregation events.^31^

A summary of the quantitative ^1^H NMR results obtained for 1 alone and when co-formulated as co-formulations a–e in a DMSO-*d*_6_ (1% DCM) and D_2_O (5% EtOH) solution at 112 mM and 5.56 mM respectively is given in Table 2. As with SSA 1 alone, co-formulations a–e showed no evidence for the formation of larger self-associated species with solid-like properties in a DMSO-*d*_6_ (1% DCM) solution.

**Table 2:** Overview of the results from quantitative ^1^H NMR studies obtained from (i) DMSO-*d*_6_, standardised with 1.0% DCM at 112 mM and; (ii) D_2_O standardised with 5.0% ethanol at 5.56 mM. Values given in % represent the observed proportion of compound to become NMR silent. All quantitative ^1^H NMR experiments were conducted with a delay time (*d*_1_) of 60 s at 298 K. Control = compound 1 only

Co-formulation	Solvent system	Anion	Cation	Co-formulant
1 only^19^	DMSO-*d*_6_	0	0	n/a
	D_2_O	51	50	n/a
a^a^	DMSO-*d*_6_	0	0	0
	D_2_O	56	57	50
b^a^	DMSO-*d*_6_	0	0	0
	D_2_O	50	49	41
c^a^	DMSO-*d*_6_	0	0	0
	D_2_O	53	29	64
d^a^	DMSO-*d*_6_	0	0	0
	D_2_O	48	55	62
e	DMSO-*d*_6_	0	0	0
	D_2_O	65	83	b

a Analogous quantitative ^1^H NMR control experiments conducted with 2–5 only in a D_2_O solution standardised with 5.0% ethanol at 5.56 mM confirmed the absence of any larger self-associated structures.

b Values could not be determined due to peak overlap.

A complimentary series of ^1^H NMR DOSY studies confirmed that under these experimental conditions, the anionic and cationic components of the SSA and appropriate co-formulant 2–6, were all shown to exhibit different diffusion rates in a DMSO-*d*_6_/0.5% H_2_O solution, meaning that these molecular components are not strongly associated to one another. The hydrodynamic diameter (*D*_H_) of the molecular species present within these solutions, calculated using the Stokes–Einstein equation from the appropriate diffusion rate measurements, are listed in Table 3. The size of the species present also indicates that as previously observed for this class of compound, where self-association of the SSA's anionic component (1) exists, it is likely to adopt a dimeric binding mode.

**Table 3:** Hydrodynamic diameter (*D*_H_) (nm) of those molecular species present in a DMSO-*d*_6_/0.5% H_2_O solution at 298 K, as determined by ^1^H NMR DOSY. Self-association dimerization constants (*K*_dim_) (M^−1^) calculated for 1 and co-formulations a–e in a DMSO-*d*_6_/0.5% H_2_O solution at 298 K. These constants were obtained from the fitting of ^1^H NMR dilution data and refined to the EK/dimerization model using BindFit v 0.5 (ref. 30)^a^

Co-formulation	*D*_H_			*K*_dim_	± % error
	SSA anion	SSA cation	Co-formulant		
1 only^19^	1.15	1.08	n/a	2.7	0.3
a	1.23	1.13	0.74	2.09	1. 5
b	1.57	1.51	1.37	4.37	0.3
c	1.69	1.74	2.21	2.92	0.8
d	1.70	1.29	1.99	4.91	0.5
e	*a*	*a*	*a*	3.67	0.4

a *a* = values could not be determined due to peak overlap. n/a = not applicable.

**Fig. 3:**
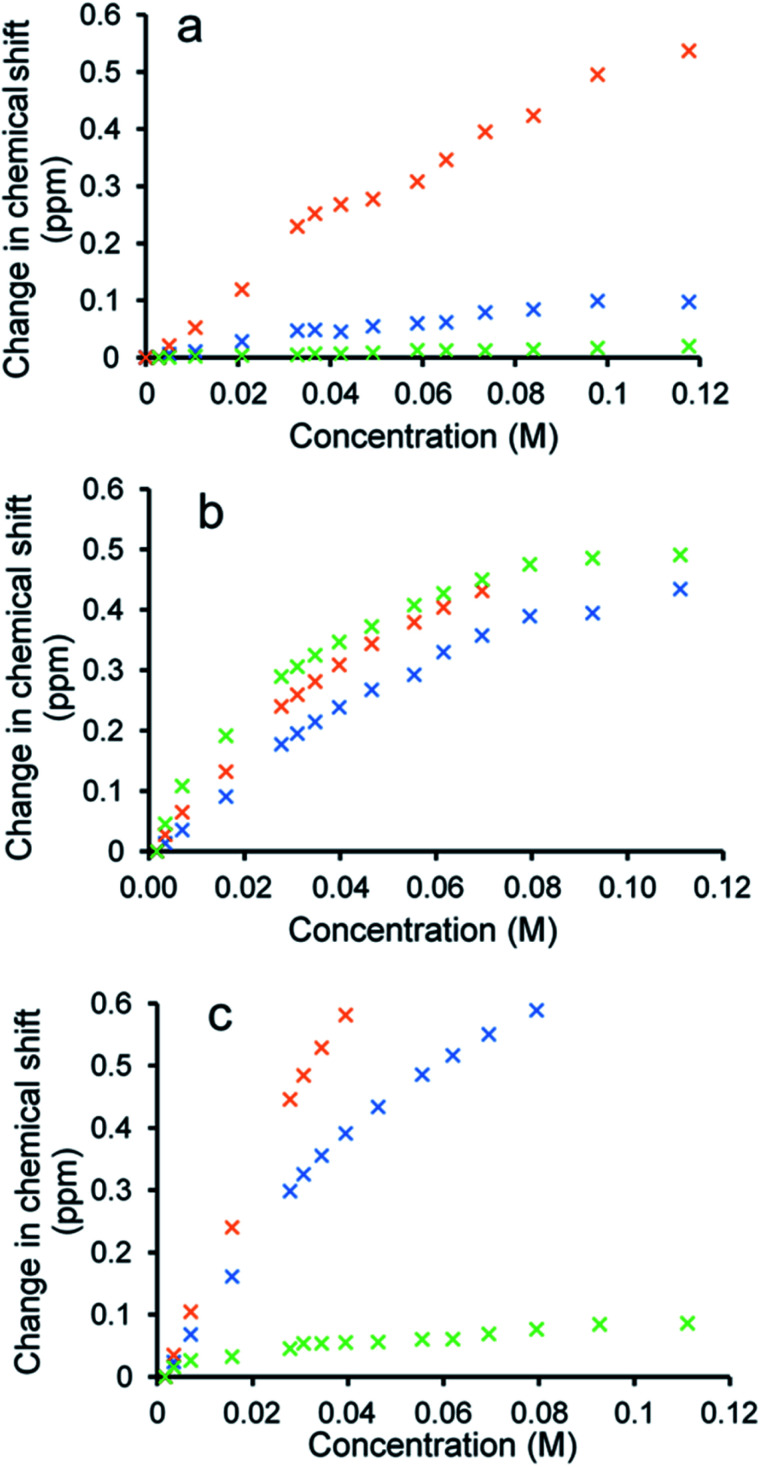
Graphs illustrating the ^1^H NMR down-field change in chemical shift of SSA (1) urea and appropriate co-formulant NH resonances in DMSO-*d*_6_/0.5% H_2_O (298 K) for: (a) co-formulation a; (b) co-formulation c; and (c) co-formulation d. The relevant NH's plotted within these graphs have been highlighted in the appropriate colour within Fig. 1.

To verify the presence of any hydrogen bonded self-association events within a DMSO-*d*_6_/0.5% H_2_O solution at 298 K, ^1^H NMR dilution studies were performed. From these data collected, the change in chemical shift for those NH resonances within the SSA anion and co-formulant were plotted with respect to concentration (Fig. 3). With these data fitted to the dimerization (EK) model using Bindfit v0.5,^30^ a *K*_dim_ of 2.7 M^−1^ was calculated for 1 only.^19^ A similar *K*_dim_ of 2.1 M^−1^ was calculated for the SSA anion in co-formulation a. This is not surprising as comparing the change in chemical shift for those NH resonances of the SSA anion and co-formulant 2, we observed a downfield change in chemical shift for the urea NH's of the anion but no discernible change for those NH resonances associated with 2, meaning that it is unlikely this co-formulant is involved within the SSA anion self-association events (Fig. 3a).

Fitting the analogous ^1^H NMR dilution data obtained for the SSA component of co-formulations b and e to the same dimerization isotherm resulted in an increased *K*_dim_ of 4.4 and 3.7 M^−1^ respectively. Unfortunately, in this instance we were unable to observe the position of the NH resonances associated with co-formulants 3 and 6. Compound 6 is a metal complex also capable of competing with any SSA self-association events. However, 3 exists as an HCl salt, therefore introducing Cl^−^ ions into the solution. It is likely that these ions will also compete with the SSA sulfonate unit to coordinate with the urea functionality and therefore, this complex self-associative event would mean that these data are no longer appropriate to fit to this binding model, so this dimerization value should be treated with caution.

When comparing the change in chemical shift values for the different components of co-formulation c, the NH resonances of the SSA anion and co-formulant (4) were found to exhibit similar changes in chemical shift with respect to concentration (Fig. 3b). This leads us to believe that co-formulant 4, is also involved in molecular association/self-association events within this solution. This means that once again the *K*_dim_ reported in Table 2 should be treated with caution due to the possible complex nature of these self-association events. It is also hypothesised that complex self-association events also occur with co-formulation d (Fig. 3c). Here the co-formulant contains a carboxylate functionality, carboxylate anions are known to form stronger complexes with a urea functionality than the sulfonate anion,^32^ therefore it is possible that this ion is preferentially coordinating with the anionic component of the SSA. Although the *K*_dim_ calculated in this instance is similar to that of 1 alone.

Moving from a DMSO-*d*_6_ solution into an aqueous D_2_O : EtOH 19 : 1 solution at an SSA/co-formulation concentration of 5.56 mM at 298 K, our quantitative ^1^H NMR control experiment (Table 2) containing 1 only shows 50% of this SSA to become incorporated into larger self-associated structures. The proportion of SSA (1) to become incorporated when supplied as co-formulation b, remains the same as with the SSA only however, 41% of 3 also becomes NMR silent. We hypothesise that this is because this proportion of 3 is now incorporated into the larger self-associated aggregates of 1. Interestingly, the presence of 2 (co-formulation a) results in approximately 5–7% more of the SSA becoming incorporated into these larger self-associated aggregates, along with 50% of the available co-formulant. We hypothesise that this may be due to the lack of competitive Cl^−^ and H^+^ ions, alongside the stabilising effects of uncharged, comparatively hydrophobic isoniazid (2) incorporation within the extended aggregate structure.

The cationic component of 4 in co-formulation c is amphiphilic in nature and in the presence of this SSA (1) 64% of this amphiphilic cation is incorporated into these larger SSA structures, replacing a portion of the SSA cation, as now 53% of the SSA anion remains incorporated into the extended aggregate structure whereas, only 29% of the SSA TBA counter cation is now utilised within aggregate formation. The reverse is true for co-formulation d. Here the carboxylate anion is incorporated into the SSA self-associated aggregate (62%), replacing a proportion of the SSA's anionic component (48%) in comparison to the SSA counter cation (55%). Interestingly, except for co-formulations a and e, the proportion of SSA anion to be incorporated into those self-associated aggregate species appears to be maintained at ≈50%.

To further characterise the formation of these larger self-associated aggregates, CMC values were also determined for co-formulations a–e and, compared to a control solution containing 1 only. These results have been summarised in Table 4. Here, co-formulation b exhibits a similar CMC value (11.21 mM) to that of 1 only (10.39 mM) however, co-formulations a, c and e, did exhibit decreased CMC values of 6.76 mM, 5.58 mM and 3.29 mM respectively. We hypothesise that this may be due to the presence of the amphiphilic/comparatively non-polar agents within these co-formulations. Interestingly the CMC obtained for co-formulation d was found to have increased significantly from that of 1 only, from 10.38 mM to 16.24 mM. It is believed that this is due to the presence of the carboxylate moiety within the structure of 5. As suggested by the results of the ^1^H NMR dilution studies, this compound perturbs the self-associative interaction of the SSA anion. Here we predict that those same competitive association events are responsible for this increase in CMC. It is therefore plausible that the slight increase in CMC observed for co-formulation b may also be due to competitive molecular association events. This hypothesis is further supported by the results of comparative dynamic light scatting and zeta potential studies (Table 4). Here a value of −76 mV was recorded for the control solution of 1 only at 5.56 mM. This value was found to decrease through the addition of 3 and 5 to −22 mV and −3 mV for co-formulations b and d respectively while, the stability of those larger aggregate structures formed with co-formulation a were found to exhibit a similar stability to 1 alone.

**Table 4:** Overview of average DLS intensity particle size distribution peak maxima (aggregate *D*_H_ measurements), zeta potential and CMC, measurements obtained for a H_2_O/5.0% EtOH solution of an SSA (1) or co-formulation a–e (5.56 mM) at 298 K^a^

Co-formulation	*D*_H_ (nm)	Polydispersity (%)	Zeta potential (mV)	CMC (mM)
1 alone^19^	164	24 (±1.20)	−76	10.39
1* alone^19^	142	25 (±0.68)	−66	n/a
a	200	20 (±1.56)	−72	6.76
b	209	17 (±1.65)	−22	11.21
c*	240	25 (±0.75)	+76	5.58
d	136	13 (±1.26)	−3	16.24
e*	160	21 (±21.51)	−42	3.29

a * = experiments were performed at 0.56 mM due to solubility issues.

**Fig. 4:**
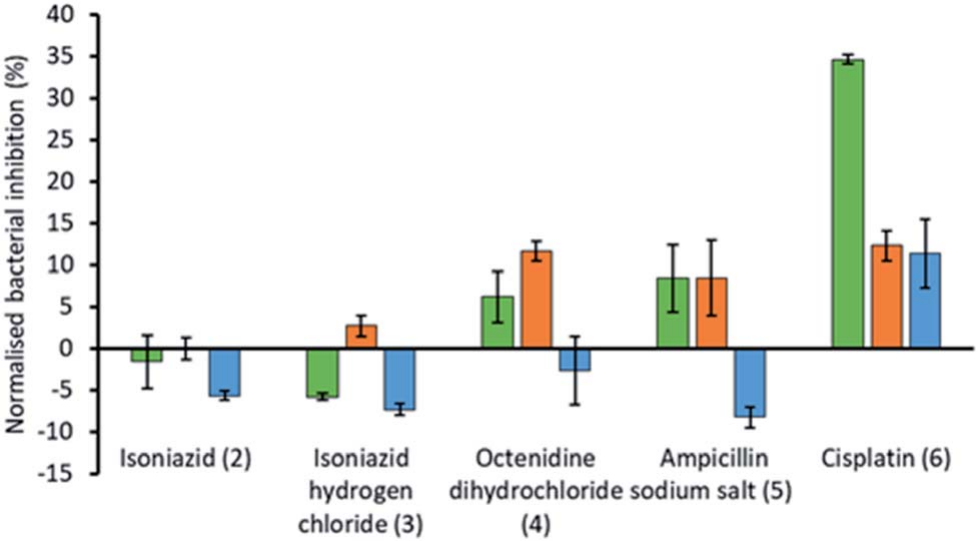
Bar chart showing the increase/decrease in efficacy of the antimicrobial/therapeutic agent (2–6) when supplied as a co-therapy in the presence of SSA 1. These results have been normalised against the combined bacterial growth inhibition effects of 1 and co-formulants 2–6 alone. OD_600_ measurements were obtained at 1100 minutes against *E. coli* DH10B in the presence of SSA 1 (1.5 mM) and either isoniazid (2) (1.45 μM), isoniazid hydrogen chloride (3) (1.73 μM), octenidine dihydrochloride (4) (0.29 μM), ampicillin sodium salt (5) (0.89 μM) or cisplatin (6) (16.6 μM). Each co-formulated system was studied where the: (i) SSA was pre-incubated with the *E. coli* for 10 min before the antimicrobial agent was added (green); (ii) antimicrobial agent was pre-incubated with the *E. coli* for 10 min before the SSA was added (orange); (iii) SSA and antimicrobial agent was added in co-formulation without any prior incubation (blue). The concentration of 2–6 alone was found to impede bacterial growth by <30% over 1100 min.

To explore the effects of the presence of an SSA on the efficacy of different currently available antimicrobial/therapeutic agents with different modes of action, *E. coli* was chosen as a model Gram-negative organism. Additionally, the effects of the order of SSA or antimicrobial/therapeutic agent addition was also investigated. Here either the SSA and antimicrobial agent was added simultaneously as the co-formulation (a–e) or, the cells were incubated firstly; with the SSA only for 10 minutes, followed by the addition of the antimicrobial agent and secondly; by incubating first with the antimicrobial agent for 10 minutes followed by the addition of the SSA. In this instance the concentrations of both the SSA (1) and antimicrobial agents (2–6) were chosen through completion of microbial susceptibility studies. These studies were conducted to ensure that both the SSA and antimicrobial/therapeutic agent were at a concentration where some inhibition of bacterial growth, and therefore antimicrobial efficacy was being imparted on the *E. coli*.† The results of these studies have been summarised for convenience within Fig. 4.

The antimicrobial/therapeutic agents were chosen for this study not only to be diverse in chemical structure but also diverse in antimicrobial mode of action. These agents include: (i) the antibiotic ampicillin (5) which inhibits the synthesis of the peptidoglycan bacterial cell wall;^33^ (ii) octenidine (4) which acts to disrupt the cell membrane,^34^ and is thought to therefore act in a similar fashion to the SSAs themselves, at least in part; (iii) cisplatin (6), which is more commonly considered an anticancer agent, but still promotes bacterial cell death through DNA cross-linking within the cell, preventing/blocking DNA replication;^35^ (iv) two variations of isoniazid (2 and 3), which is traditionally used to treat mycobacteria infections through inhibition of mycolic acid synthesis.^36^

From these studies we have shown that when *E. coli* undergoes combination therapy with both an antimicrobial agent and an SSA, the presence of the SSA is able to enhance the activity of the antimicrobial agent in the cases of octenidine (4), ampicillin (5) and cisplatin (6). However, interestingly the degree of antimicrobial efficacy enhancement was found to be dependent on the order in which those agents were supplied as part of the appropriate combination therapy to be studied. When the SSA and antimicrobial agent were supplied to the *E. coli* as a co-formulation (a–e), with all co-formulations apart from co-formulation e there is an antagonistic effect, where bacterial growth is decreased when compared to the additive effects of the single SSA or antimicrobial agent alone. However, the reverse is true for co-formulation e where there is an agonistic effect. This is hypothesised to be because co-formulation e retains similar properties to that of the SSA alone at comparative concentrations. As we have previously shown a decrease in CMC value of an SSA was found to correlate with the MIC_50_ value obtained for a series of 50 SSAs against this same strain of bacteria, where the CMC value is *ca.* ≥11 mM.^15^ Here the presence of cisplatin (6), supplied in a 1 : 1 ratio with SSA 1 was shown to lower the CMC from 10.39 mM to 3.29 mM, which we suggest could increase SSA antimicrobial activity in line with these previous observations. Additionally, the size and stability of those self-associated structures produced in the presence of cisplatin (6) also retain some similarity (Table 4).

The presence of an SSA was not found to increase the efficacy of isoniazid, either when supplied as the neutral compound (2), or as the hydrochloric acid salt (3), with the exception of preincubation of the hydrochloric acid salt with the bacteria. However, this increase in efficacy is very small. The greatest increase in antimicrobial efficacy was observed for 1 with cisplatin (6). Here an enhancement of 34.6%, 12.3% and 11.4% in bacterial growth inhibition was observed with prior incubation of the bacteria with SSA (1), prior incubation of the bacteria with cisplatin (6) and the SSA co-formulated with the cisplatin before addition, respectively (Fig. 4). Thus, the greatest enhancement in antimicrobial efficacy was observed when the cells were incubated with the SSA, prior to the addition of the cisplatin (6).

Additionally, SSA 1 was also confirmed to slightly enhance the efficacy of both ampicillin (5) and octenidine (4), which impart antimicrobial effects through disruption of either the cell wall (ampicillin) or cell membrane (octenidine). However, these enhancement effects were only observed when the two agents were supplied to the cells as a combination therapy (one after another) and not when added simultaneously as a co-formulation. From evidence collected from our physicochemical analysis we hypothesise that this is because these antimicrobial agents interfere with SSA self-association, as observed within the scope of our ^1^H NMR dilution study experiments (Fig. 3b and c). In disrupting SSA self-association and membrane interaction events, we believe that this decreases the proportion of therapeutic agents to arrive at the surface of the cell in an active form, due to competitive multicomponent interactions between antimicrobial/therapeutic agents at the cell surface.

## Conclusions

As one of the greatest threats to human health remains the rise of antimicrobial resistant bacterial infections, particularly those caused by Gram-negative bacteria,^37^ new technologies are required to combat this threat. Here, we have shown that the self-associated aggregates formed by SSA 1 are capable of interacting with antimicrobial/therapeutic agents currently in use. However, of particular interest are the observed differences in SSA/antimicrobial combination therapy over co-formulation efficacy brought about by changing the order of agent addition to bacteria.

Here, we have confirmed that 1 is able to increase the efficacy of cisplatin (6) (a DNA chelating agent), ampicillin (5) (an antibiotic that disrupts cell wall synthesis) and octenidine (4) (a membrane disrupting antiseptic). However, in the case of 4 and 5 an antagonistic effect was observed when these agents were added as a co-formulation. The summation of the evidence presented here in leads us to conclude that SSA 1 acts most effectively as an antimicrobial efficacy enhancer when supplied as a combination therapy, through a prior 10 minute incubation period, before the addition of the antimicrobial/therapeutic agent. We also hypothesise, through data provided from a range of complimentary SSA co-formulation physicochemical studies, that the degree of antimicrobial effect maybe due to a combination of either destructive or constructive molecular interaction events at the cell surface.

## Conflicts of interest

There are no conflicts to declare.

## Supplementary Material

RA-011-D1RA00998B-s001

RA-011-D1RA00998B-s002

## References

[cit1] Bérdy J. (2012). J. Antibiot..

[cit2] Davey P., Marwick C. A., Scott C. L., Charani E., Mcneil K., Brown E., Gould I. M., Ramsay C. R., Michie S. (2017). Cochrane Database Syst. Rev..

[cit3] Morley P. S., Apley M. D., Besser T. E., Burney D. P., Fedorka-Cray P. J., Papich M. G., Traub-Dargatz J. L., Weese J. S. (2005). J. Vet. Intern. Med..

[cit4] Marshall B. M., Levy S. B. (2011). Clin. Microbiol. Rev..

[cit5] Brown E. D., Wright G. D. (2016). Nature.

[cit6] Bock L. J., Hind C. K., Sutton J. M., Wand M. E. (2018). Lett. Appl. Microbiol..

[cit7] Mott B. , Payne D., Riley M., Rubio A., Therapeutics S., Silver L., Silver Consulting L., Silverman J., Sutcliffe J., Pharmaceuticals T., Tomaras A., Tommasi R., Therapeutics E. and Zurawski D., A report from A Scientific Roadmap for Antibiotic Discovery, 2016

[cit8] Davies S. , Farrar J., Rex J., White L. J., Murry R. and O'Neill J., The Review on Antimicrobial Resistance: Tackling a crisis for the health and wealth of nations, ed. H. Government, 2014, pp. 1–14

[cit9] Li X., Bai H., Yang Y., Yoon J., Wang S., Zhang X. (2018). Adv. Mater..

[cit10] Pandurangan K., Kitchen J. A., Blasco S., Paradisi F., Gunnlaugsson T. (2014). Chem. Commun..

[cit11] Zhang M., Zhu P.-P., Xin P., Si W., Li Z.-T., Hou J.-L. (2017). Angew. Chem., Int. Ed..

[cit12] Yu Q., Deng T., Lin F. C., Zhang B., Zink J. I. (2020). ACS Nano.

[cit13] Wu F., He D., Chen L., Liu F., Huang H., Dai J., Zhang S., You J. (2018). RSC Adv..

[cit14] Ng K. K. L., Dimitrovski M., Boles J. E., Ellaby R. J., White L. J., Hiscock J. R. (2020). Supramol. Chem..

[cit15] Allen N., White L. J., Boles J. E., Williams G. T., Chu D. F., Ellaby R. J., Shepherd H. J., Ng K. K. L., Blackholly L. R., Wilson B., Mulvihill D. P., Hiscock J. R. (2020). ChemMedChem.

[cit16] White L., Boles J. E., Allen N., Alesbrook L., Sutton J. M., Hind C., Hilton K., Blackholly L. R., Ellaby R., Williams G., Mulvihill D. P., Hiscock J. R. (2020). J. Mater. Chem. B.

[cit17] White L. J., Boles J. E., Hilton K. L. F., Ellaby R. J., Hiscock J. R. (2020). Molecules.

[cit18] White L. J., Wells N. J., Blackholly L. R., Shepherd H. J., Wilson B., Bustone G. P., Runacres T. J., Hiscock J. R. (2017). Chem. Sci..

[cit19] White L. J., Tyuleva S. N., Wilson B., Shepherd H. J., Ng K. K. L., Holder S. J., Clark E. R., Hiscock J. R. (2018). Chem.–Eur. J..

[cit20] Tyuleva S. N., Allen N., White L. J., Pépés A., Shepherd H. J., Saines P. J., Ellaby R. J., Mulvihill D. P., Hiscock J. R. (2019). Chem. Commun..

[cit21] Gumbs T. L., White L. J., Wells N. J., Shepherd H. J., Hiscock J. R. (2018). Supramol. Chem..

[cit22] Faustino C. M. C., Calado A. R. T., Garcia-Rio L. (2009). J. Phys. Chem. B.

[cit23] Faustino C. M. C., Calado A. R. T., Garcia-Rio L. (2010). J. Colloid Interface Sci..

[cit24] Scozzafava A., Mastrolorenzo A., Supuran C. T. (2001). J. Enzyme Inhib..

[cit25] Townshend G., Thompson G. S., White L. J., Hiscock J. R., Ortega-Roldan J. L. (2020). Chem. Commun..

[cit26] Medina-Carmona E., Varela L., Hendry A. C., Thompson G. S., White L. J., Boles J. E., Hiscock J. R., Ortega-Roldan J. L. (2020). Chem. Commun..

[cit27] Hiscock J. R., Bustone G. P., Wilson B., Belsey K. E., Blackholly L. R. (2016). Soft Matter.

[cit28] Sarcevica I., Orola L., Veidis M. V., Belyakov S. (2014). Acta Crystallogr., Sect. C: Struct. Chem..

[cit29] Blackholly L. R., Shepherd H. J., Hiscock J. R. (2016). CrystEngComm.

[cit30] Supramolecular.org , Binding Constant Calculators|Supramolecular, http://supramolecular.org/, accessed 1 June 2020

[cit31] Von Krbek L. K. S., Schalley C. A., Thordarson P. (2017). Chem. Soc. Rev..

[cit32] Gale P. A., Hiscock J. R., Lalaoui N., Light M. E., Wells N. J., Wenzel M. (2012). Org. Biomol. Chem..

[cit33] Kaushik D., Mohan M., Borade D. M., Swami O. C. (2014). J. Clin. Diagn. Res..

[cit34] Malanovic N., Ön A., Pabst G., Zellner A., Lohner K. (2020). Int. J. Antimicrob. Agents.

[cit35] Chowdhury N., Wood T. L., Martínez-Vázquez M., García-Contreras R., Wood T. K. (2016). Biotechnol. Bioeng..

[cit36] Timmins G. S., Deretic V. (2006). Mol. Microbiol..

[cit37] Breijyeh Z., Jubeh B., Karaman R. (2020). Molecules.

[cit38] Sheldrick G. M. (2015). Acta Crystallogr., Sect. A: Found. Crystallogr..

[cit39] Sheldrick G. M. (2015). Acta Crystallogr., Sect. C: Struct. Chem..

[cit40] Dolomanov O. V., Bourhis L. J., Gildea R. J., Howard J. A. K., Puschmann H. (2009). J. Appl. Crystallogr..

