# Supplementary material for: Supramolecular self-associating amphiphiles (SSAs) as enhancers of antimicrobial agents towards Escherichia coli (E. coli)
Source: RSC Adv. 2021 Mar 3;11(16):9550–6. doi: 10.1039/d1ra00998b (PMC8695399; doi:10.1039/d1ra00998b)
Supplement: RA-011-D1RA00998B-s001 [file RA-011-D1RA00998B-s001.pdf]

Electronic Supplementary Material (ESI) for RSC Advances.
This journal is © The Royal Society of Chemistry 2021

Supramolecular Self-associating Amphiphiles (SSAs) as enhancers of antimicrobials agents towards *Escherichia coli (E. coli)*.

Jessica E.Boles, [a] Rebecca J. Ellaby,[a] Helena J. Shepherd[a] and Jennifer R. Hiscock*[a]

Contents

Chemical Structures 2
Experimental 4
Biological experiments 5
Chemical synthesis 6
NMR 6
Characterisation NMR 6
^{1}H DOSY NMR experiments 16
Overview 22
Quantitative ^{1}H NMR experiments 23
Overview 31
^{1}H NMR self-association studies 32
Overview 42
Dynamic Light Scattering 43
Zeta potential 51
Surface Tension measurements and Critical micelle concentrations (CMC) determination 54
Overview 56
Single Crystal X-ray structures 57
Co-formulation growth curves 58
References 70

Chemical Structures

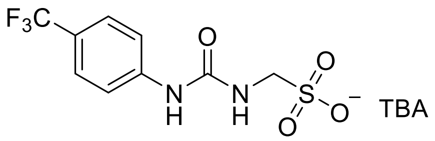

1

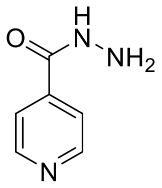

2

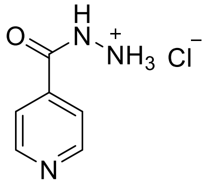

3

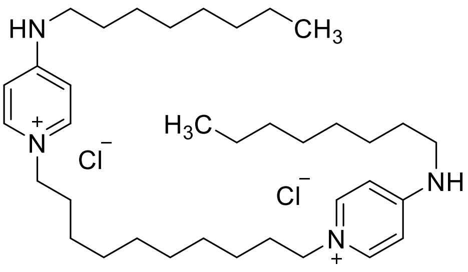

4

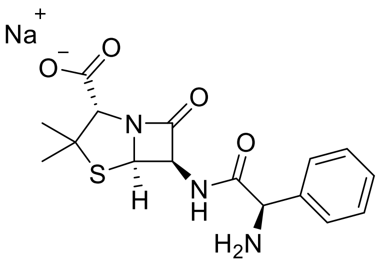

5

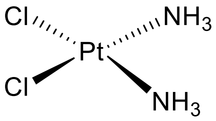

6

Figure S1 – Chemical structures of SSA **1** and co-formulant agents **2**-**6**. TBA = tetrabutylammonium

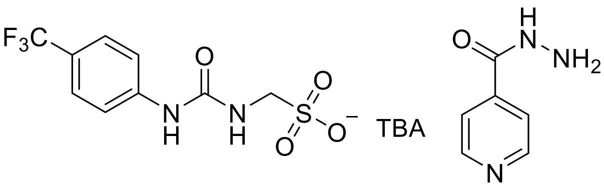

Co-formulation a

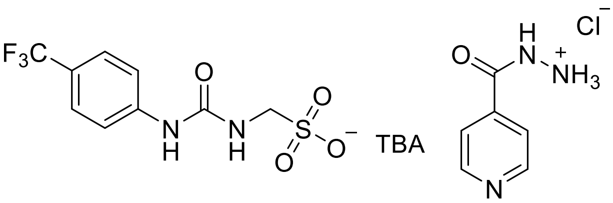

Co-formulation b

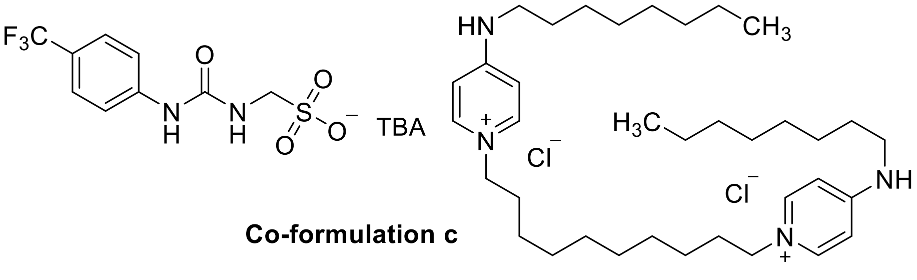

Co-formulation c

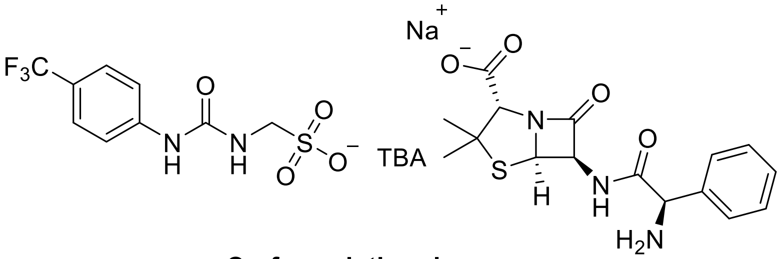

Co-formulation d

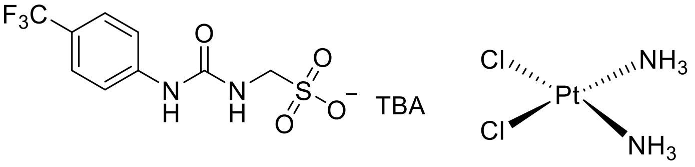

Co-formulation e

Figure S2 - Chemical structures of co-formulations **a**-**e**. TBA = tetrabutylammonium

Experimental

General remarks: A positive pressure of nitrogen and oven dried glassware were used for all reactions. All solvents and starting materials were purchased from known chemical suppliers or available stores and used without any further purification unless specifically stipulated. The NMR spectra were obtained using a Burker AV2 400 MHz or AVNEO 400 MHz spectrometer. The data was processed using ACD Labs. NMR Chemical shift values are reported in parts per million (ppm) and calibrated to the centre of the residual solvent peak set (s = singlet, br = broad, d = doublet, t = triplet, q = quartet, m = multiplet). Tensiometry measurements were undertaken using the Biolin Scientific Theta Attension optical tensiometer. The data was processed using Biolin OneAttension software. A Hamilton (309) syringe was used for the measurements. The melting point for each compound was measured using Stuart SMP10 melting point apparatus. DLS and Zeta Potential studies were carried out using Anton Paar Litesizer^{TM} 500 and processed using KalliopeTM Professional. Cellular growth curve measurements obtained using Thermo Scientific Multiscan Go 1510-0318C plate reader and recorded using the SkanIt Software 4.0 and a Clariostar plater reader using MARS data analysis software.

Self-association constant calculation: Self-association constants were determined using Bindfit v0.5 (http://app.supramolecular.org/bindfit/). All the data can be accessed online using the hyperlinks provided.

Tensiometry Studies: All the samples were prepared in an EtOH:H_2O (1:19) solution. All samples underwent an annealing process in which the various solutions were heated to approximately 40 °C before being allowed to cool to room temperature, allowing each sample to reach a thermodynamic minimum. All samples were prepared through serial dilution of the most concentrated sample. Three surface tension measurements were obtained for each sample at a given concentration, using the pendant drop method. The average values were then used to calculate the critical micelle concentration (CMC).

DLS Studies: All vials used for preparing the samples were clean and dry. All solvents used were filtered to remove any particulates that may interfere with the results obtained. Samples of differing concentrations were obtained through serial dilution of a concentrated solution. All samples underwent an annealing process, in which they were heated to 40 °C before being allowed to cool to 25 °C. A series of 9 runs were recorded at 25 °C.

Zeta Potential Studies: All vials used for preparing the samples were clean and dry. All solvents used were filtered to remove any particulates that may interfere with the results obtained. All samples underwent an annealing process in which the various solutions were heated to approximately 40 °C before cooling to room temperature, allowing each sample to reach a thermodynamic minimum. The final zeta potential value given is an average of the number of experiments conducted at 25 °C.

Single Crystal X-ray Studies: A suitable crystal of each amphiphile was selected and mounted on a Rigaku Oxford Diffraction Supernova diffractometer. Data were collected using Cu Kα radiation at 100 K or 293 K as necessary due to crystal instability at lower temperatures. Structures were solved with the ShelXT[1] or ShelXS structure solution programs via Direct Methods and refined with ShelXL[2] by Least Squares minimisation. Olex2[3] was used as an interface to all ShelX programs (CCDC 1999018).

Biological experiments

Preparation of Luria Broth media (LB): Yeast extract (5 g), tryptone (10 g) and sodium chloride (10 g) were dissolved in dH_2O (1 L) then divided into bottles and autoclaved.

Preparation of Luria Broth (LB) agar plates: Agar (6 g) was added to LB (400 mL) and autoclaved. Once cool, the LB agar was poured into sterile petri dishes under sterile conditions and allowed to set. LB plates were stored at 4 °C until use.

Preparation of bacterial plates: Sterile LB agar plates were streaked using *Escherichia coli* then incubated at 37 °C overnight.

Preparation of Inoculum: An initial culture was made up by inoculating LB media (5 mL) with a single colony of bacteria under sterile conditions and incubated at 37 °C overnight. The following day, a subculture was made up using LB (5 mL) and the initial culture (50 µL), then incubated at 37 °C until the culture had reached an optical density (OD) of 0.4 at 600 nm. Optical Density was adjusted using sterile dH_2O to equal 0.5 McFarland standard (107 – 108 cfu/mL), then a 1:10 dilution was carried out using sterile dH_2O (900 µL) and the McFarland adjusted suspension (100 µL). A final dilution (1:100) was carried using the 1:10 suspension (150 µL) and LB (14.85 mL) before use to achieve a final cell concentration of 105 cfu/mL.

Preparation of 96 well microplate for co-formulation plates: Solutions of compound **1** were made up using 5 % ethanol in water. Solutions of co-formulations **a**-**e** were made up on a 1:1 molar ratio with compound **1** and the desired drug (**2**-**6**) in 5 % ethanol. A 1:100 cell suspension (150 µL) was aliquoted into each well. A co-formulation solution (**a**-**e**) (30 µL) was added to give a final well volume of 180 µL. The plates were incubated for 20 hours in a plate reader, with OD_{600} readings at 15-minute intervals. Optical density readings were plotted against time to produce growth curves. The optical density at 1100 mins was used to study synergistic interactions.

Preparation of 96 well microplate for SSA preincubation plates: Solutions of compound **1** were made up using 5 % ethanol in water. A 1:100 cell suspension (150 µL) was aliquoted into each well. Compound **1** solution (15 µL) was added to each well and the plate was pre-incubated for 10 mins at 37 °C before addition of co-formulant (**2**-**6**) (15 µL). These were incubated for 20 hours in a plate reader, with OD_{600} readings at 15-minute intervals. Optical density readings were plotted against time to produce growth curves. The optical density at 1100 mins was used to study synergistic interactions.

Preparation of 96 well microplate for drug preincubation plates: Solutions of compound **1** were made up using 5 % ethanol in water. A 1:100 cell suspension (150 µL) was aliquoted into each well. Co-formulant solution (**2**-**6**) (15 µL) was added to each well and the plate was pre-incubated for 10 mins at 37 °C before addition of compound **1** (15 µL). These were incubated for 20 hours in a plate reader, with OD_{600} readings at 15-minute intervals. Optical density readings were plotted against time to produce growth curves. The optical density at 1100 mins was used to study synergistic interactions.

Chemical synthesis

Compound 1: This compound was synthesised in line with our previously published methods. Proton NMR was found to match our previously published values.[4] ^{1}H NMR (400 MHz, 298K, DMSO-d_6): δ: 9.24 (s, 1H), 7.55 (dd, J = 8.72 Hz, 4H), 6.88 (s, 1H), 3.92 (d, J = 5.96 Hz, 2H), 3.17 - 3.13 (m, 8H), 1.60-1.52 (m, 8H), 1.34 - 1.25 (m, 8H), 0.92 (t, J = 7.36 Hz, 12H).

NMR

Characterisation NMR

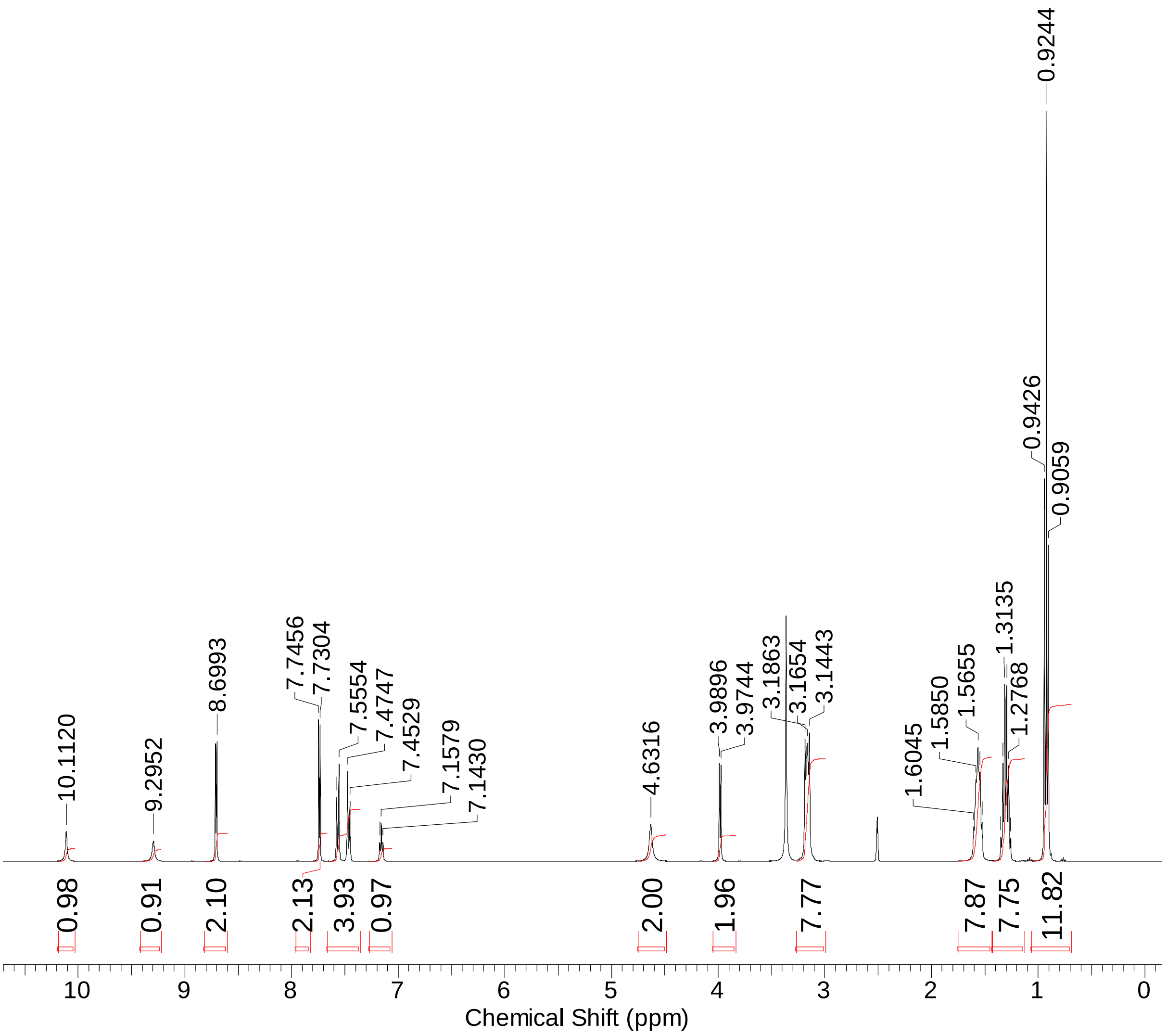

Figure S3 - ^{1}H NMR of co-formulation **a** in DMSO-d_6 conducted at 298 K.

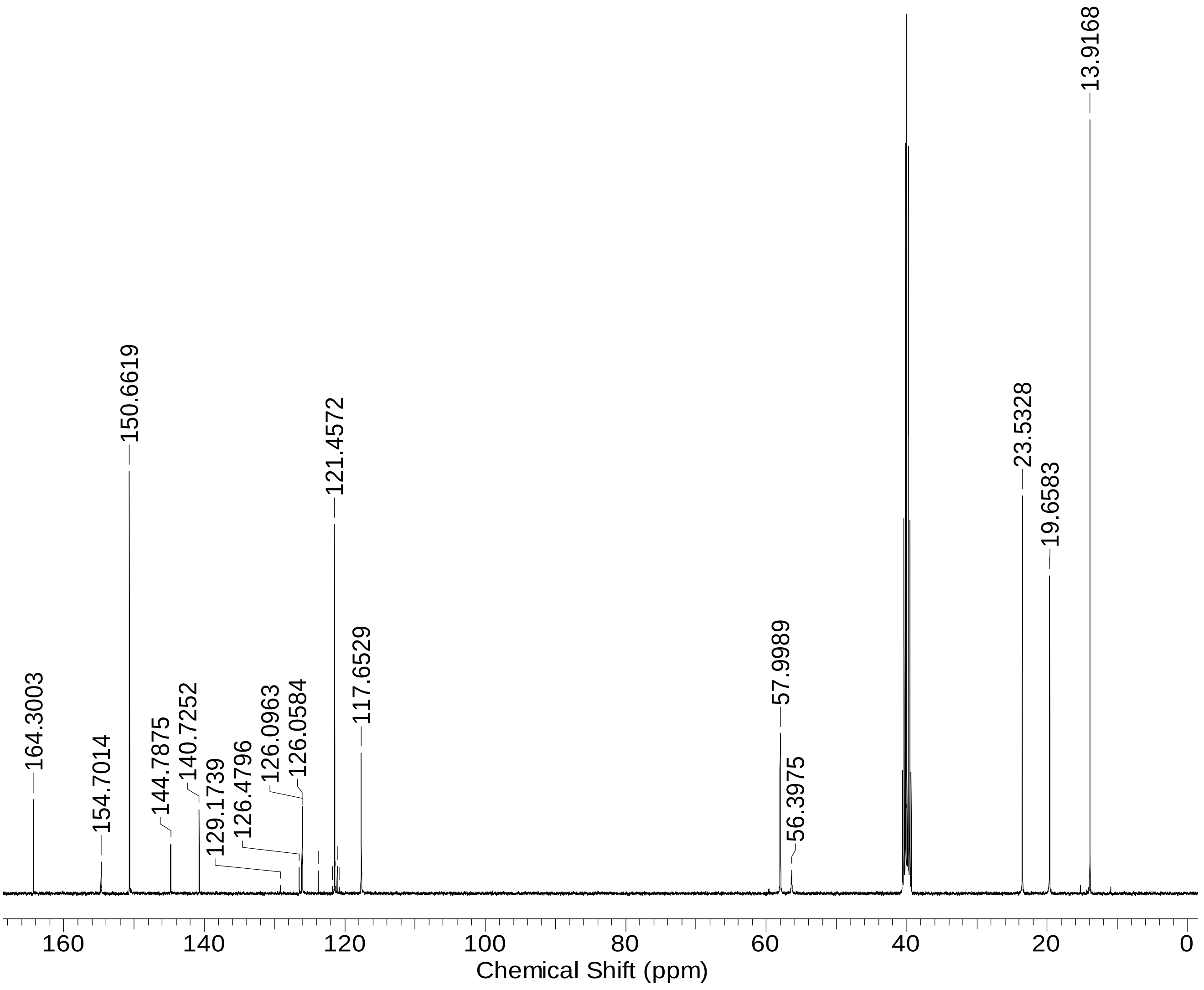

Figure S4 - ^{13}C NMR of co-formulation **a** in DMSO-d_6 conducted at 298 K.

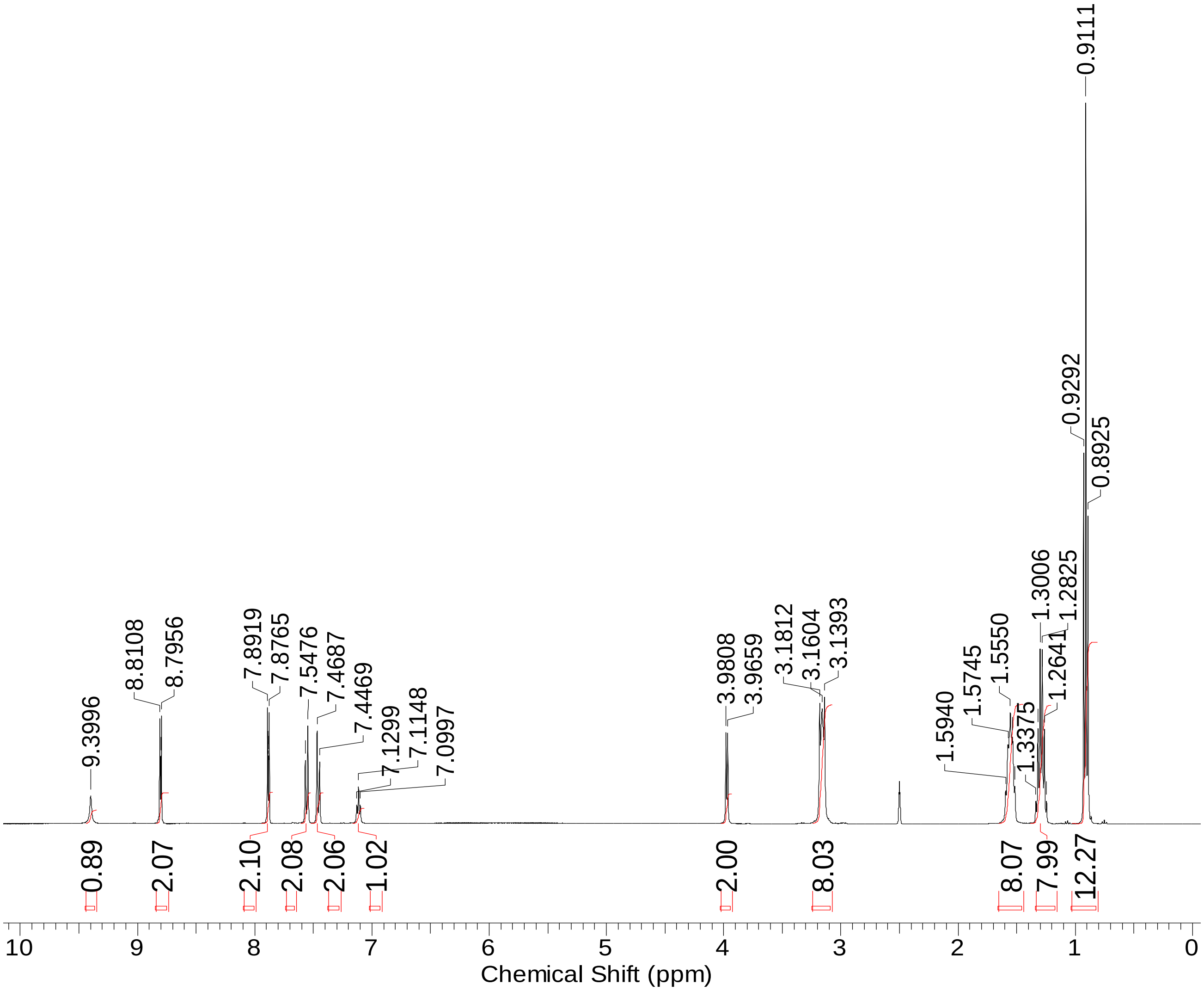

Figure S5 - ^{1}H NMR of co-formulation **b** in DMSO-d_6 conducted at 298 K.

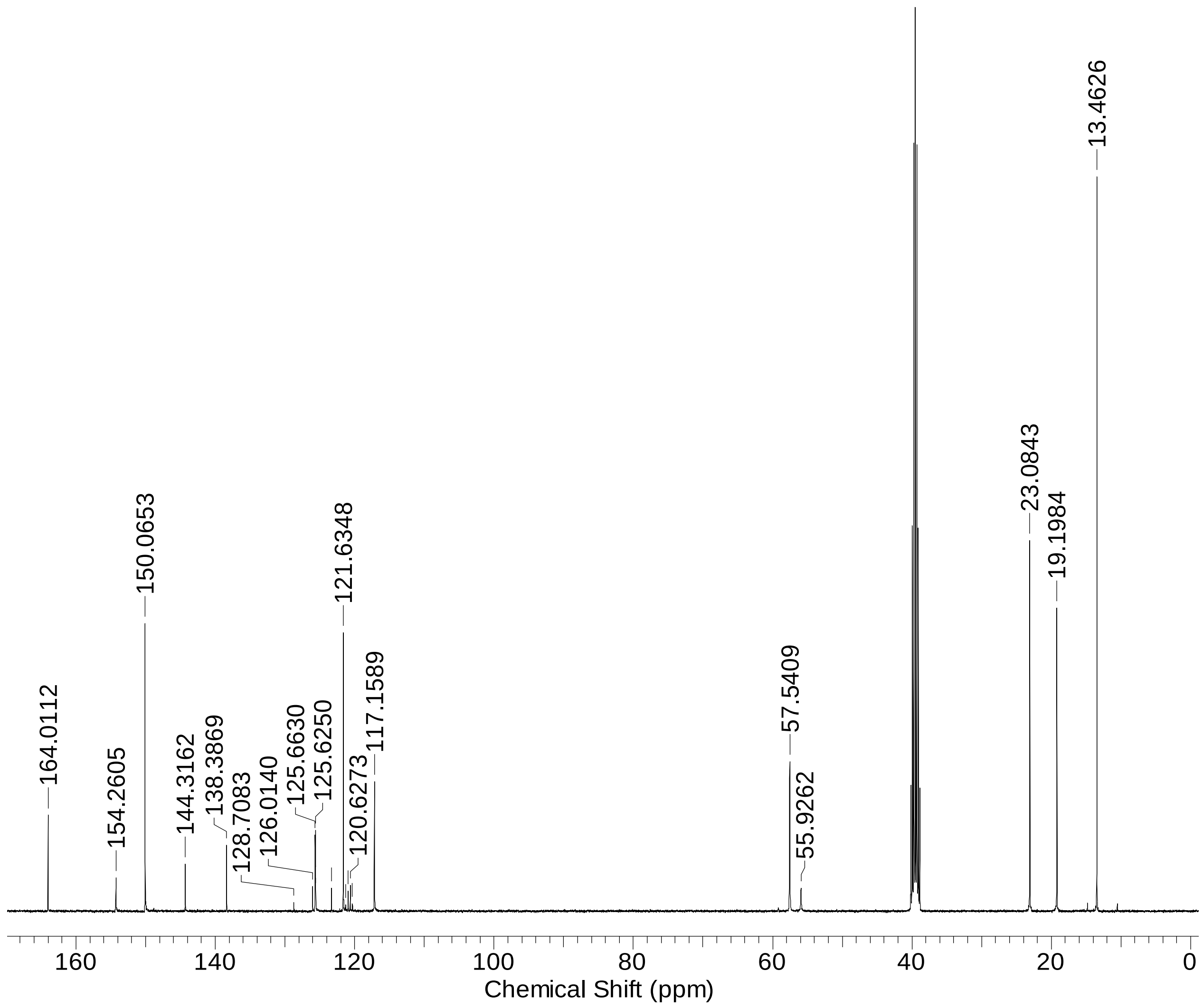

Figure S6 - ^{13}C NMR of co-formulation **b** in DMSO-d_6 conducted at 298 K.

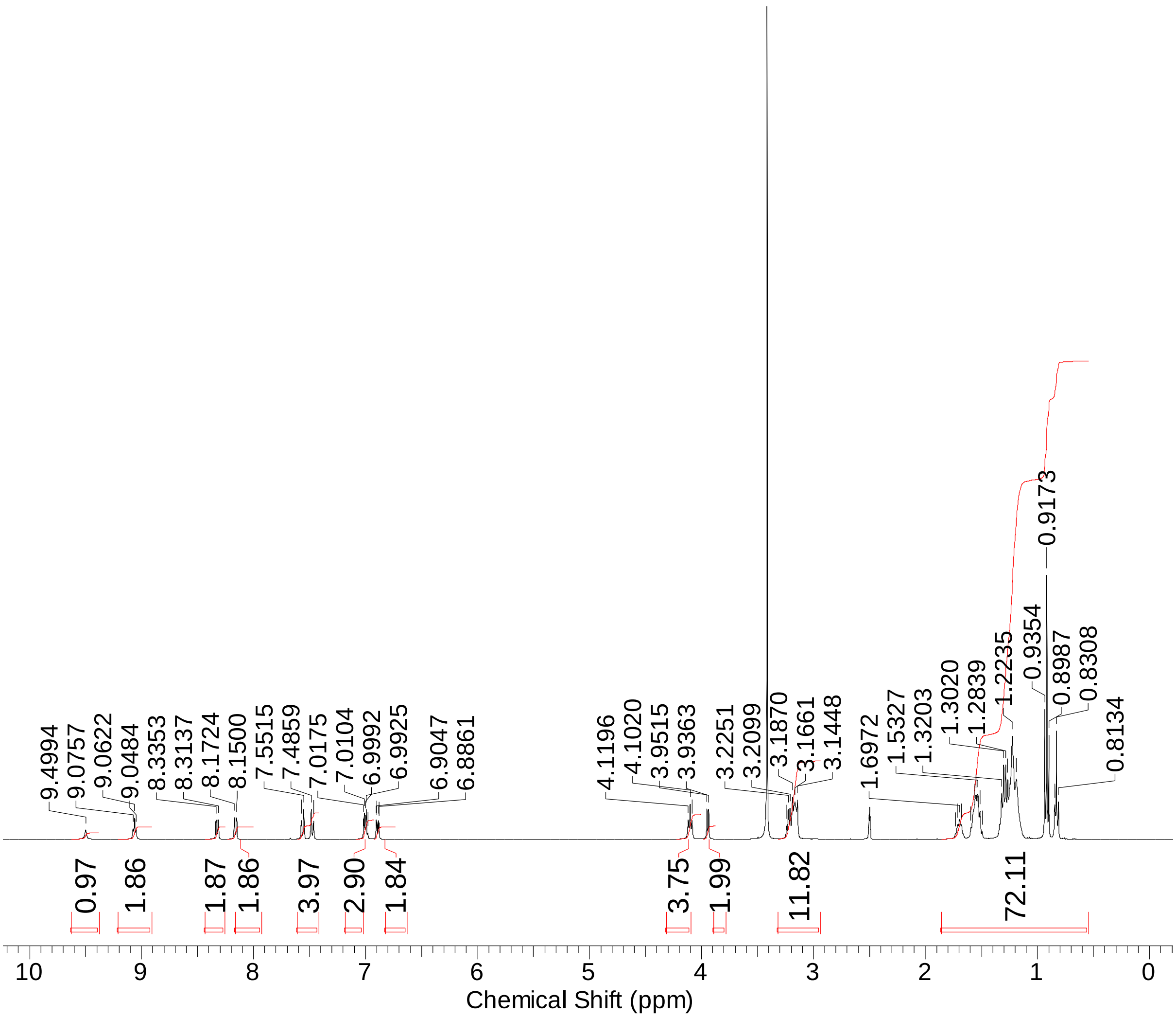

Figure S7 - ^{1}H NMR of co-formulation **c** in DMSO-d_6 conducted at 298 K.

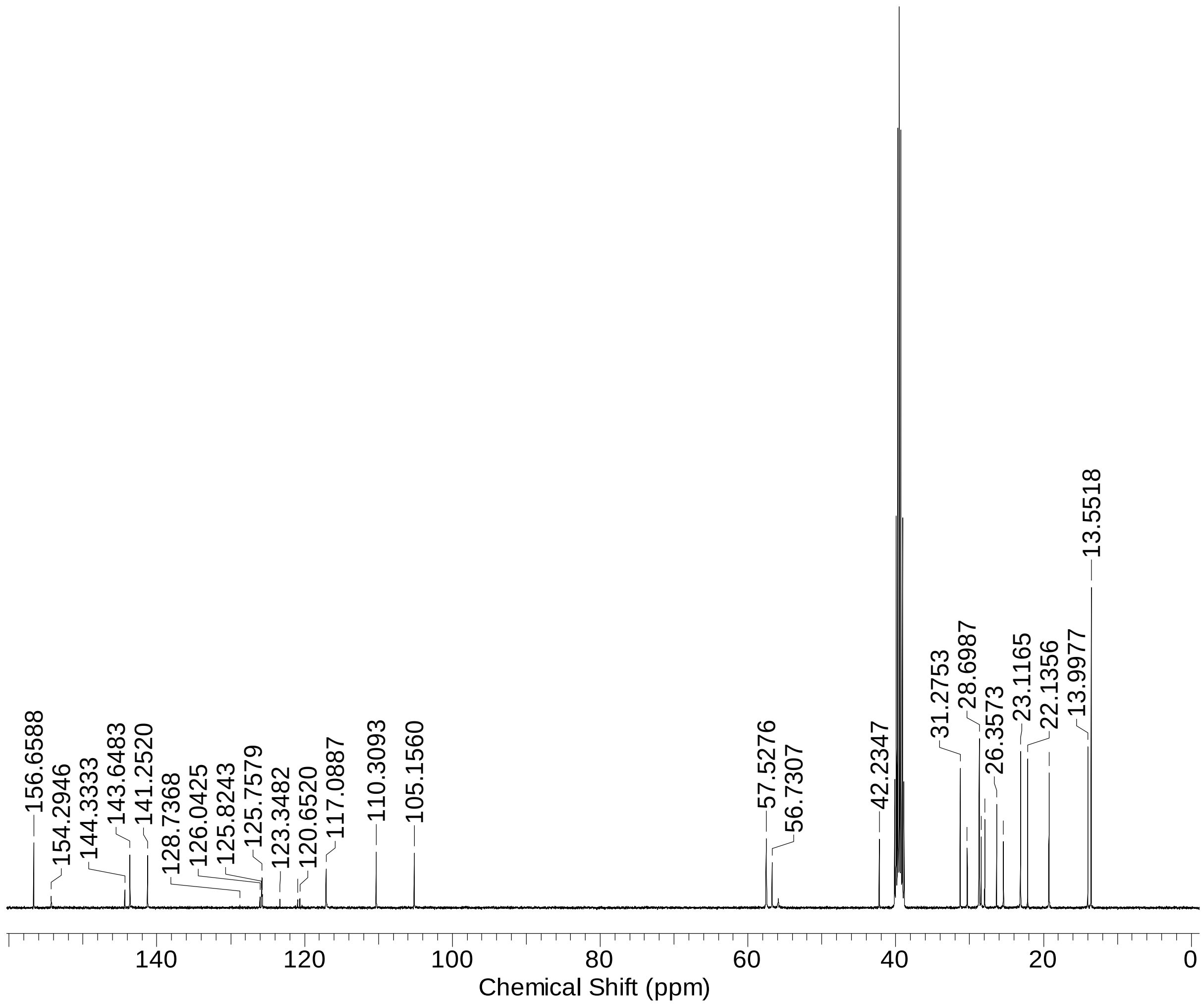

Figure S8 - ^{13}C NMR of co-formulation **c** in DMSO-d_6 conducted at 298 K.

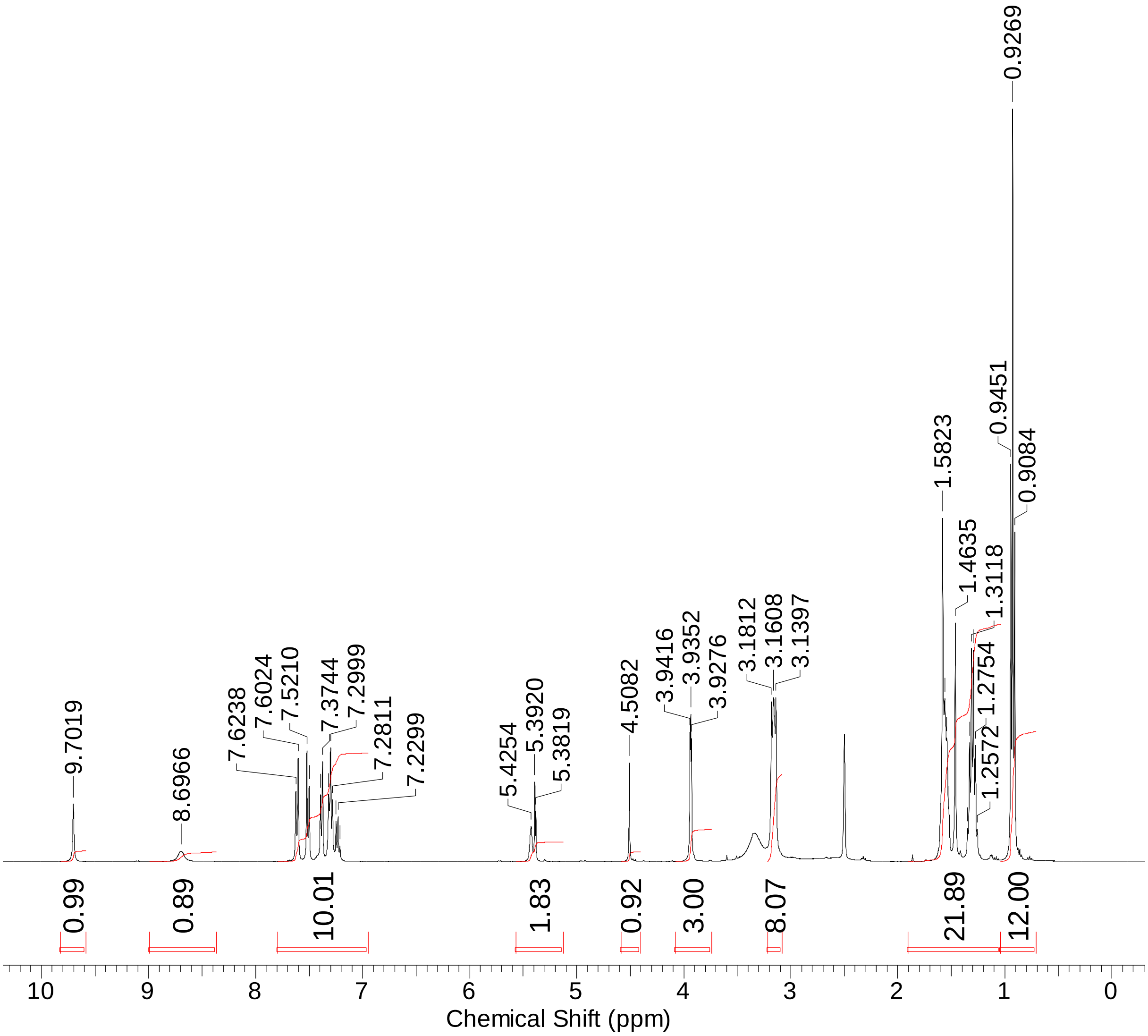

Figure S9 - ^{1}H NMR of co-formulation **d** in DMSO-d_6 conducted at 298 K.

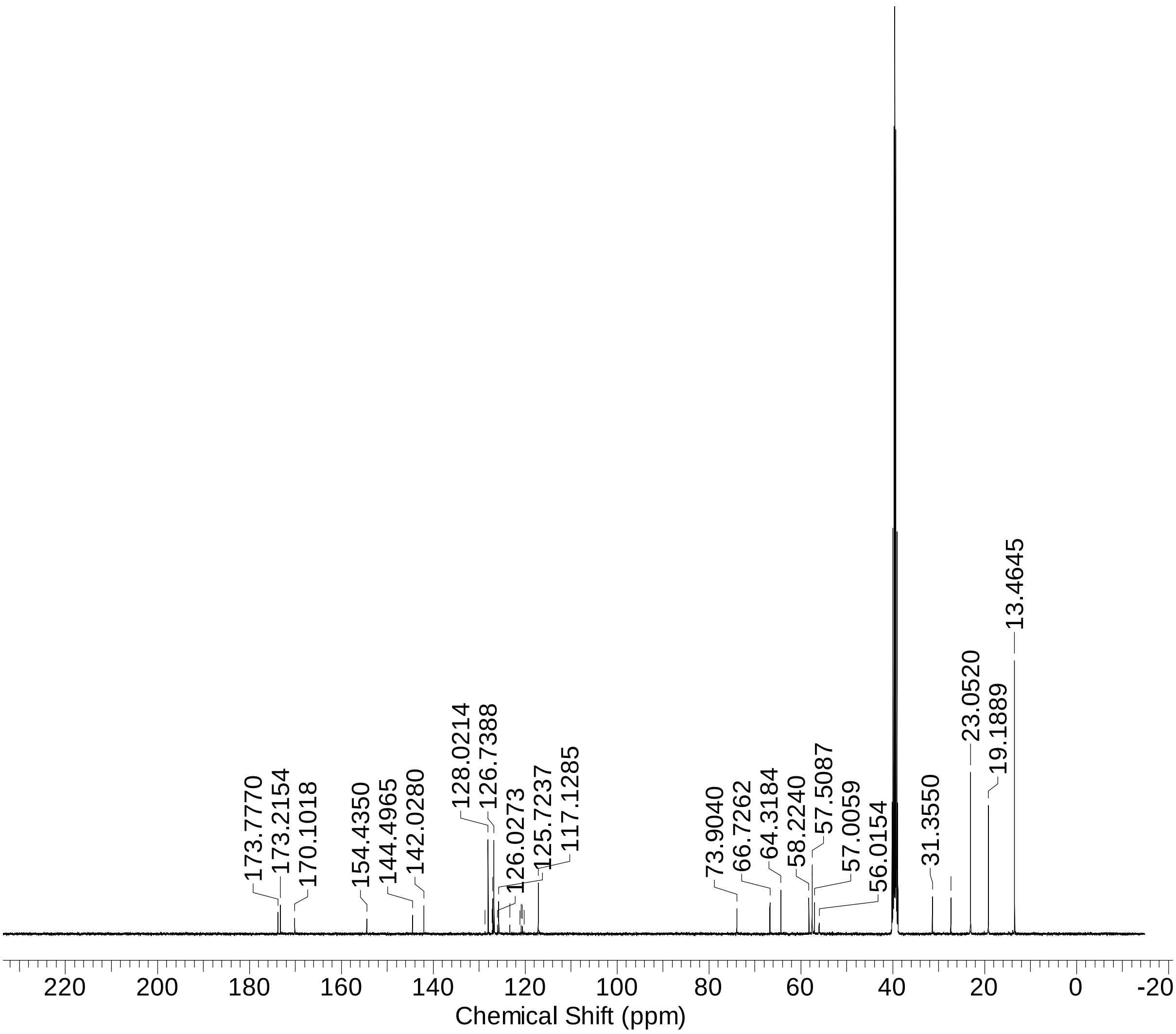

Figure S10 - ^{13}C NMR of co-formulation **d** in DMSO-d_6 conducted at 298 K.

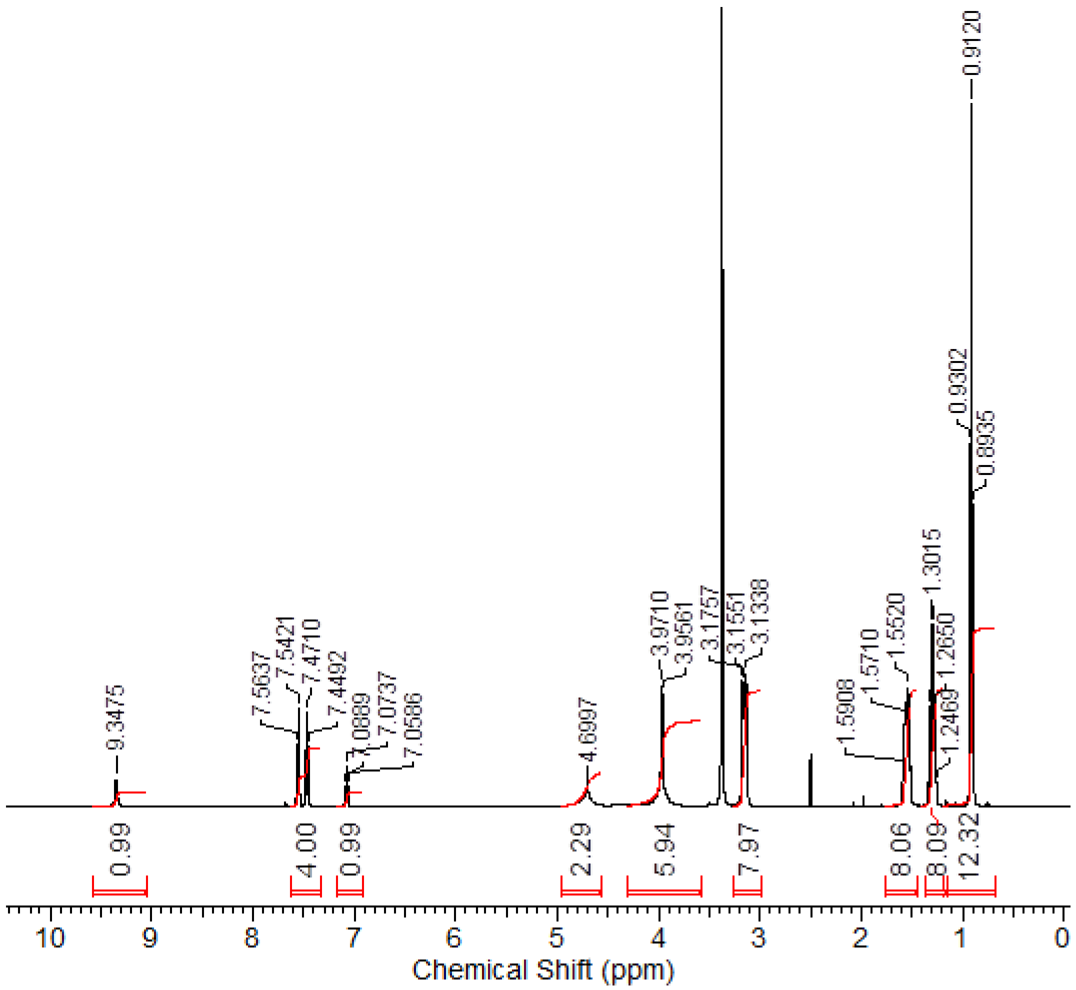

Figure S11 - ^{1}H NMR of co-formulation **e** in DMSO-d_6 conducted at 298 K.

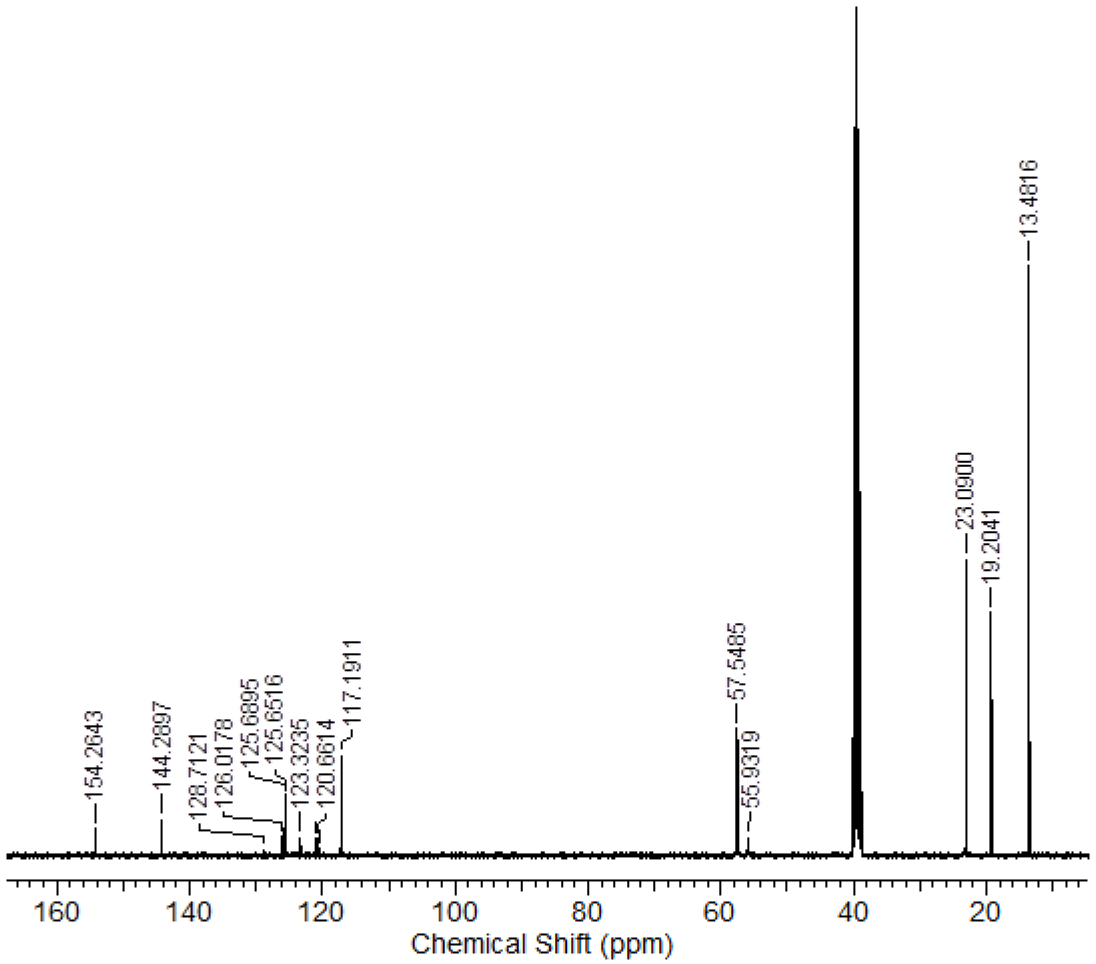

Figure S12 - ^{13}C NMR of co-formulation **e** in DMSO-d_6 conducted at 298 K.

^1H DOSY NMR experiments

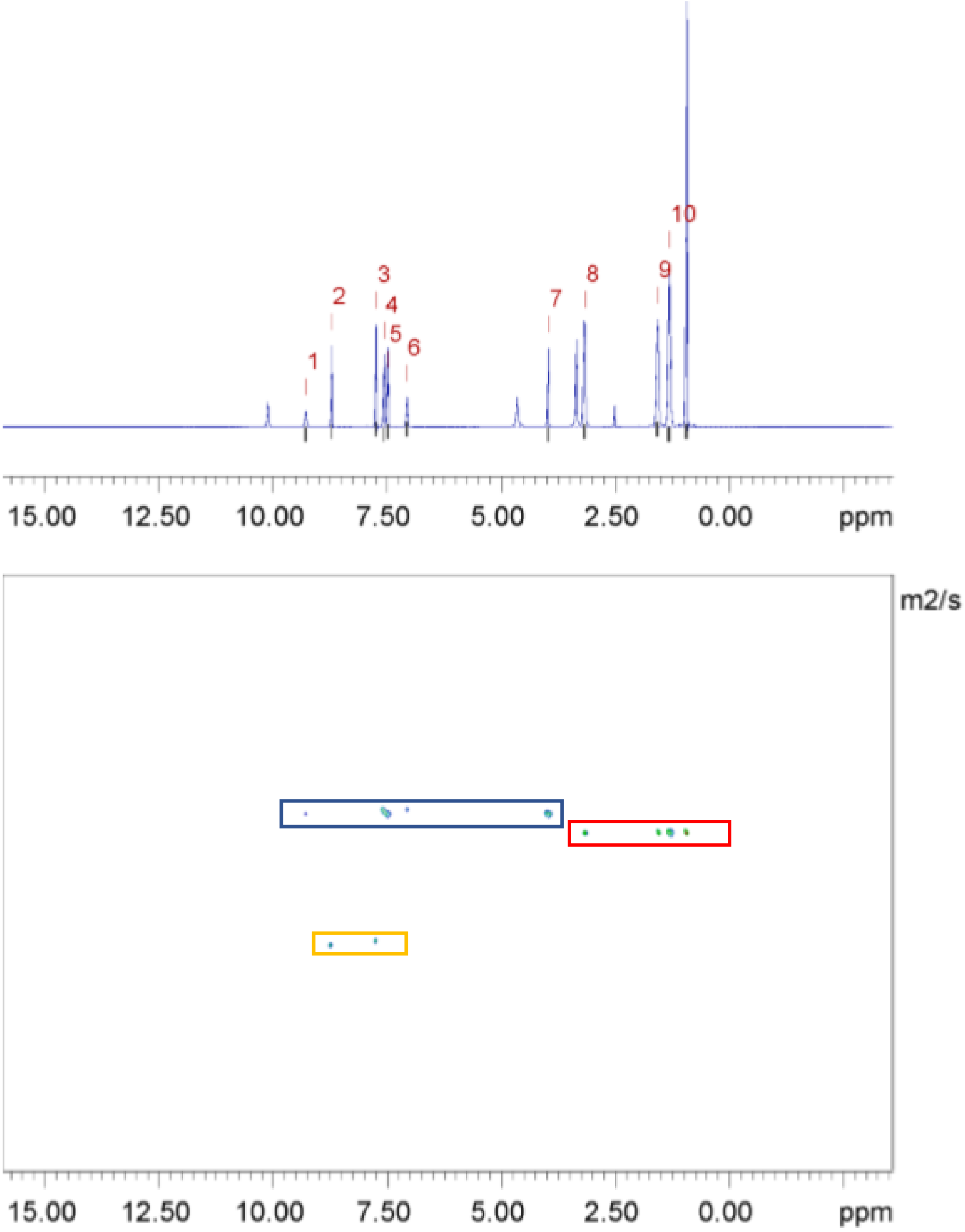

Peak name	F2 [ppm]	Io	error	D [m2/s]	error
1	9.279	1.15e+09	1.246e+05	1.80e-10	4.258e-14
2	8.709	3.61e+09	1.181e+05	2.95e-10	2.042e-14
3	7.739	4.13e+09	1.179e+05	2.94e-10	1.777e-14
4	7.566	3.96e+09	1.054e+05	1.79e-10	1.041e-14
5	7.482	4.24e+09	1.151e+05	1.80e-10	1.063e-14
6	7.067	1.76e+09	1.185e+05	1.79e-10	2.641e-14
7	3.964	3.76e+09	1.046e+05	1.79e-10	1.090e-14
8	3.166	1.12e+10	1.425e+05	1.95e-10	5.354e-15
9	1.567	1.16e+10	1.467e+05	1.94e-10	5.306e-15
10	1.309	1.47e+10	1.466e+05	1.94e-10	4.212e-15
11	0.935	2.61e+10	1.377e+05	1.94e-10	2.218e-15

Figure S13 - ^{1}H DOSY NMR spectrum of co-formulation **a** (111.6 mM) in DMSO-d_6 at 298 K and a table eporting the diffusion constants calculated for each peak used to determine the hydrodynamic diameter of the anionic components of ***a*** (d_H = 1.23 nm). Peaks 1, 4-7 correspond to the anionic component of **a** while peaks 8-11 correspond to the cationic component of **a**. Peaks 2-3 correspond to the drug component, isoniazid.

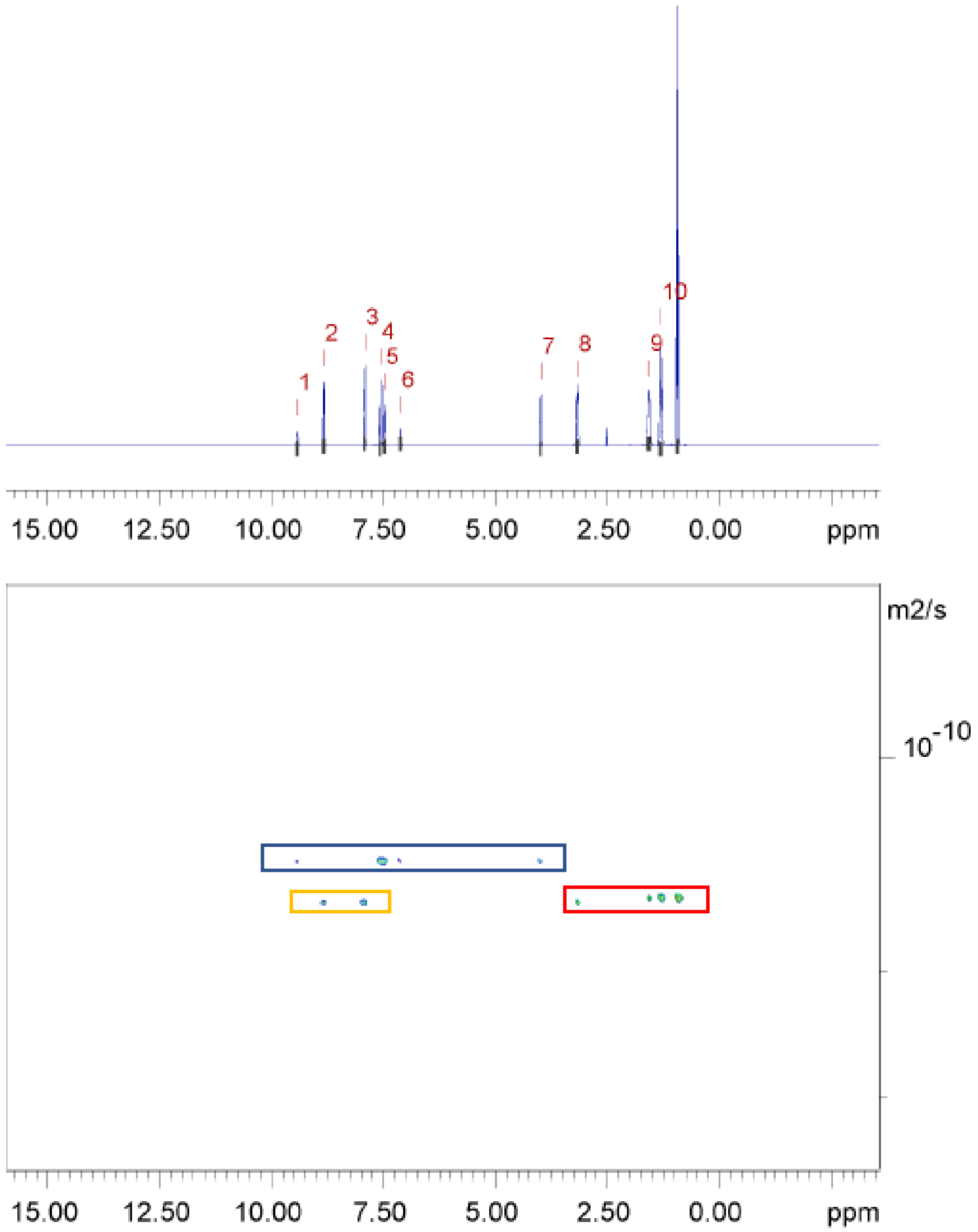
1
2
3
4
5
6
7
8
9
10
15.00
12.50
10.00
7.50
5.00
2.50
0.00
ppm
m2/s
10-10

Peak name	F2 [ppm]	Io	error	D [m2/s]	error
1	9.417	7.53e+08	5.190e+04	1.40e-10	2.112e-14
2	8.827	3.04e+09	5.390e+04	1.60e-10	6.127e-15
3	7.915	3.18e+09	4.951e+04	1.60e-10	5.382e-15
4	7.564	2.74e+09	5.699e+04	1.40e-10	6.358e-15
5	7.470	2.69e+09	5.580e+04	1.40e-10	6.348e-15
6	7.110	9.24e+08	6.292e+04	1.40e-10	2.083e-14
7	3.981	2.33e+09	5.582e+04	1.40e-10	7.347e-15
8	3.166	6.32e+09	8.105e+04	1.59e-10	4.417e-15
9	1.560	7.36e+09	9.104e+04	1.57e-10	4.196e-15
10	1.300	9.69e+09	9.271e+04	1.57e-10	3.248e-15
11	0.911	1.62e+10	7.274e+04	1.57e-10	1.517e-15

Figure S14 - ^{1}H DOSY NMR spectrum of co-formulation **b** (112.6 mM) in DMSO-d_6 at 298 K and a table reporting the diffusion constants calculated for each peak used to determine the hydrodynamic diameter of the anionic components of **b** (d_H = 1.57 nm). Peaks 1, 4-7 correspond to the anionic component of **b** while peaks 8-11 correspond to the cationic component of **b**. Peaks 2-3 correspond to the drug component, isoniazid hydrogen chloride.

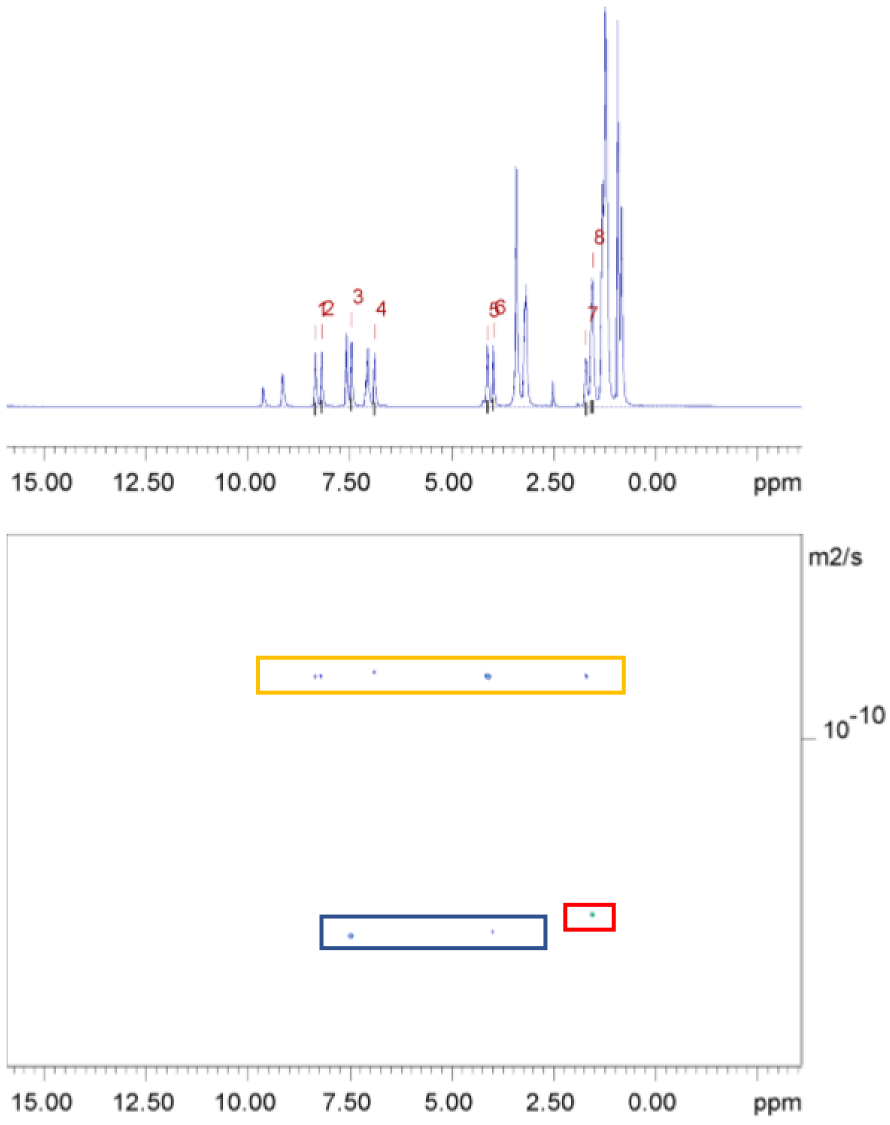

Peak name	F2 [ppm]	Io	error	D [m2/s]	error
1	8.353	3.85e+09	1.722e+05	9.22e-11	8.818e-15
2	8.190	3.82e+09	1.680e+05	9.18e-11	8.652e-15
3	7.461	5.06e+09	1.861e+05	1.30e-10	1.002e-14
4	6.893	3.64e+09	1.602e+05	9.17e-11	8.649e-15
5	4.118	4.86e+09	1.725e+05	9.17e-11	6.977e-15
6	3.982	3.77e+09	1.734e+05	1.30e-10	1.251e-14
7	1.703	4.83e+09	1.881e+05	9.17e-11	7.652e-15
8	1.550	1.78e+10	2.490e+05	1.26e-10	3.714e-15

Figure S15 - ^{1}H DOSY NMR spectrum of co-formulation **c** (112.2 mM) in DMSO-d_6 at 298 K and a table reporting the diffusion constants calculated for each peak used to determine the hydrodynamic diameter of the anionic components of **c** (d_H = 1.69 nm). Peaks 3 and 6 correspond to the anionic component of **c** while peak 8 corresponds to the cationic component of **c**. Peaks 1-2, 4-5 and 7 correspond to the drug component, octenidine dihydrochloride. Note, some signals were excluded due to poor fitting.

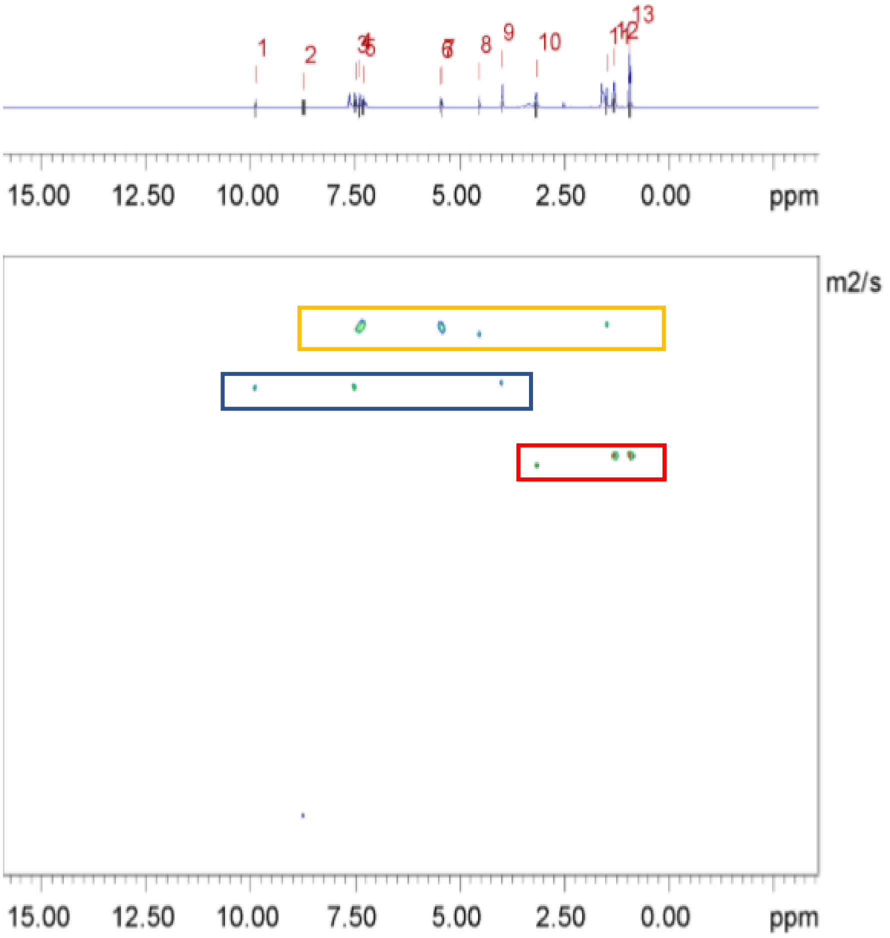

Peak name	F2 [ppm]	Io	error	D [m2/s]	error
1	9.877	1.83e+09	1.042e+05	1.30e-10	1.622e-14
2	8.724	5.43e+08	2.410e+05	4.48e-10	4.091e-13
3	7.496	4.92e+09	1.111e+05	1.30e-10	6.406e-15
4	7.395	4.16e+09	9.989e+04	1.10e-10	5.864e-15
5	7.307	4.70e+09	1.206e+05	1.09e-10	6.226e-15
6	5.455	1.62e+09	8.172e+04	1.09e-10	1.220e-14
7	5.414	1.77e+09	7.367e+04	1.11e-10	1.018e-14
8	4.526	1.54e+09	6.384e+04	1.12e-10	1.028e-14
9	3.988	1.26e+09	4.928e+04	1.28e-10	1.092e-14
10	3.167	8.88e+09	1.335e+05	1.64e-10	5.325e-15
11	1.483	3.42e+09	7.567e+04	1.09e-10	5.351e-15
12	1.308	1.48e+10	1.487e+05	1.59e-10	3.447e-15
13	0.928	2.62e+10	1.280e+05	1.59e-10	1.676e-15

Figure S16 - ^{1}H DOSY NMR spectrum of compounds **d** (114.6 mM) in DMSO-d_6 at 298 K and a table reporting the diffusion constants calculated for each peak used to determine the hydrodynamic diameter of the anionic components of **d** (d_H = 1.69 nm). Peaks 1, 3 and 9 correspond to the anionic component of **d** while peaks 10, 12-13 correspond to the cationic component of **d**. Peaks 4-8 and 11 correspond to the drug component, ampicillin.

Overview

Table S1 – Overview of diffusion coefficients (m^2/s) for compound **1** only and co-formulations **a**-**d** in DMSO-d_6 at 298 K. Errors for diffusion constants are no greater than ± 1 x 10^{-13} m^2/s.

Co-formulation	Anion	Cation	Co-formulant
Compound **1** only[5]	1.90 x 10^{-10}	2.03 x 10^{-10}	n/a
**a**	1.79 x 10^{-10}	1.94 x 10^{-10}	2.95 x 10^{-10}
**b**	1.40 x 10^{-10}	1.45 x 10^{-10}	1.60 x 10^{-10}
**c**	1.30 x 10^{-10}	1.26 x 10^{-10}	9.91 x 10^{-11}
**d**	1.29 x 10^{-10}	1.60 x 10^{-10}	1.10 x 10^{-10}
**e**	*a*	*a*	*a*

a = Experiment not performed due to peak overlap.

Table S2 - Overview of hydrodynamic diameters (nm) for compound **1** only and co-formulations **a**-**d** in DMSO-d_6 at 298 K.

Co-formulation	Anion	Cation	Co-formulant
Compound **1** only[5]	1.15	1.08	n/a
**a**	1.23	1.13	0.74
**b**	1.57	1.51	1.37
**c**	1.69	1.74	2.21
**d**	1.70	1.29	1.99
**e**	*a*	*a*	*a*

a = Experiment not performed due to peak overlap.

Quantitative ^{1}H NMR experiments

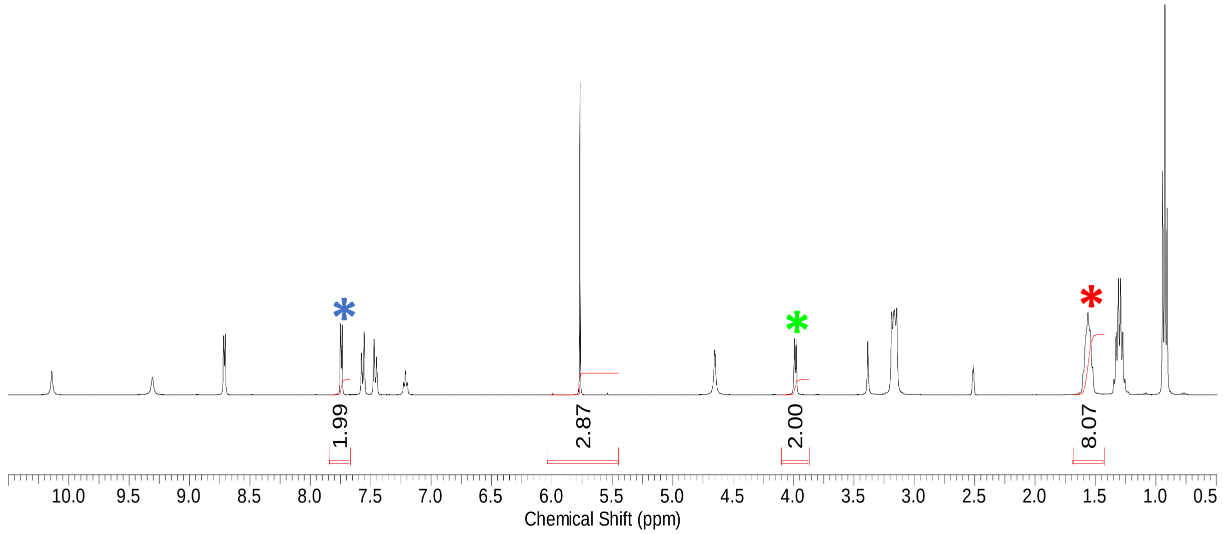

Figure S17 - ^{1}H NMR spectrum with a delay (d_1 = 60 s) of co-formulation **a** (111.4 mM) in DMSO-d_6/ 1.0 % DCM. Comparative integration indicated 0 % of the sample has become NMR silent. (Isoniazid*, anionic component of the SSA*, TBA counter cation*).

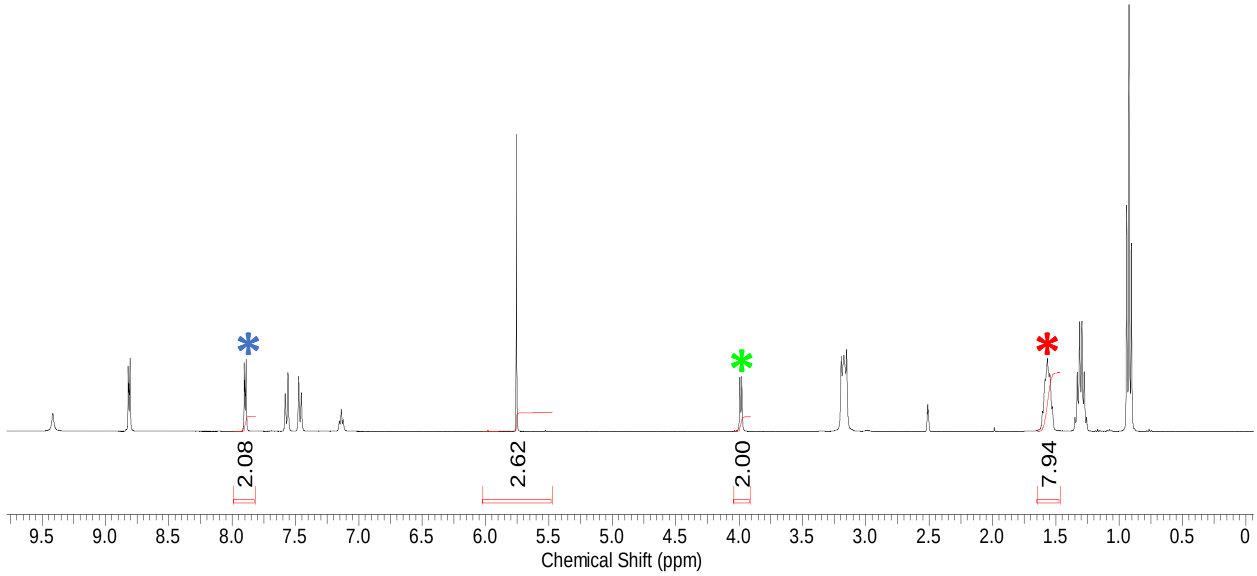

Figure S18 - ^{1}H NMR spectrum with a delay (d_1 = 60 s) of co-formulation **b** (122.1 mM) in DMSO-d_6/ 1.0 % DCM. Comparative integration indicated 0 % of the sample has become NMR silent. (Isoniazid hydrogen chloride*, anionic component of the SSA*, TBA countercation*).

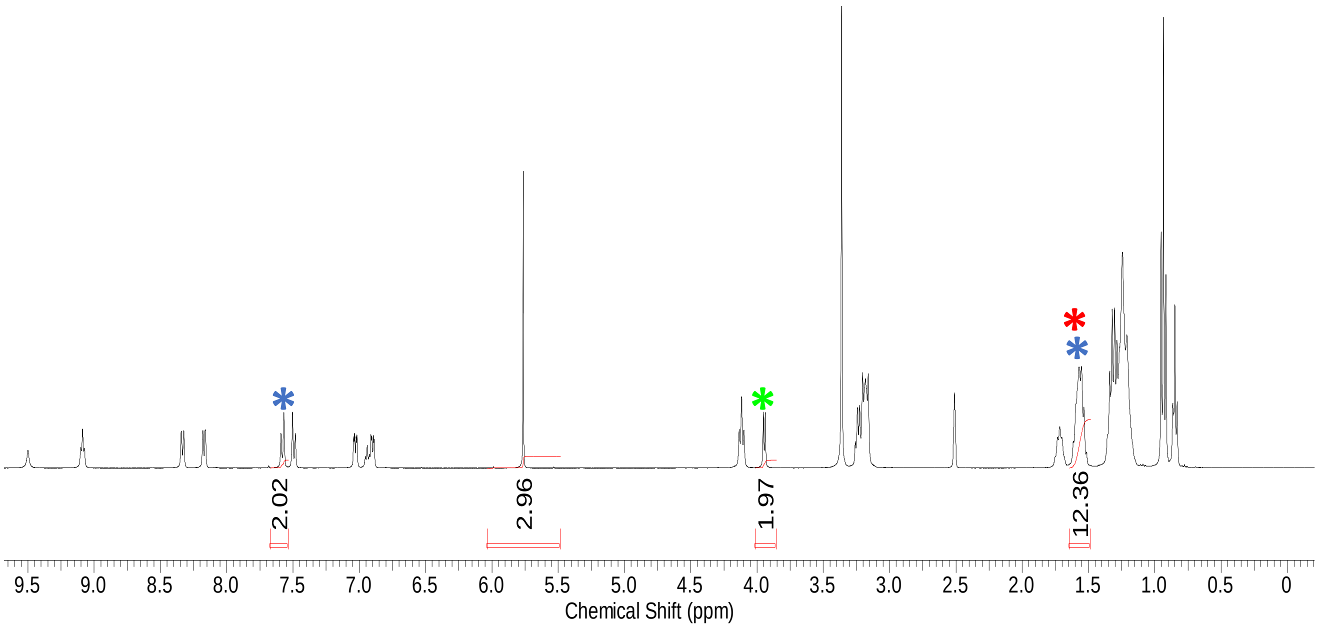

Figure S19 - ^{1}H NMR spectrum with a delay (d_1 = 60 s) of co-formulation **c** (108.1 mM) in DMSO-d_6/ 1.0 % DCM. Comparative integration indicated 0 % of the sample has become NMR silent. (Octenidine dihydrochloride*, anionic component of the SSA*, TBA countercation*).

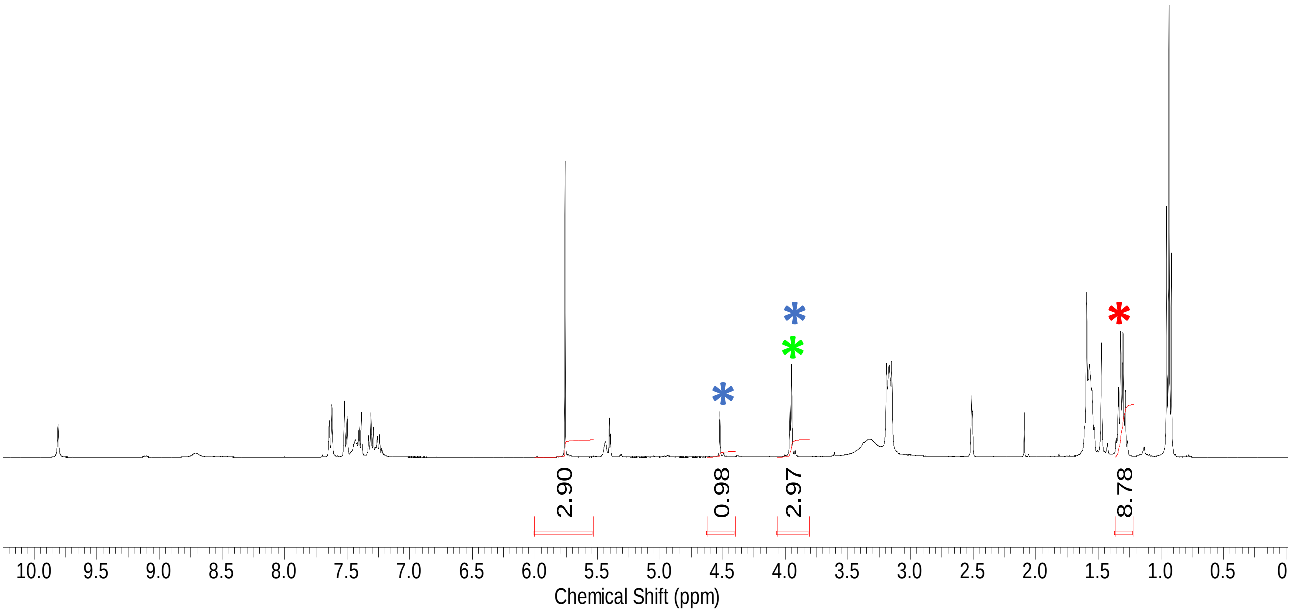

Figure S20 - ^{1}H NMR spectrum with a delay (d_1 = 60 s) of co-formulation **d** (110.0 mM) in DMSO-d_6/ 1.0 % DCM. Comparative integration indicated 0 % of the sample has become NMR silent. (Ampicillin*, anionic component of the SSA*, TBA countercation*).

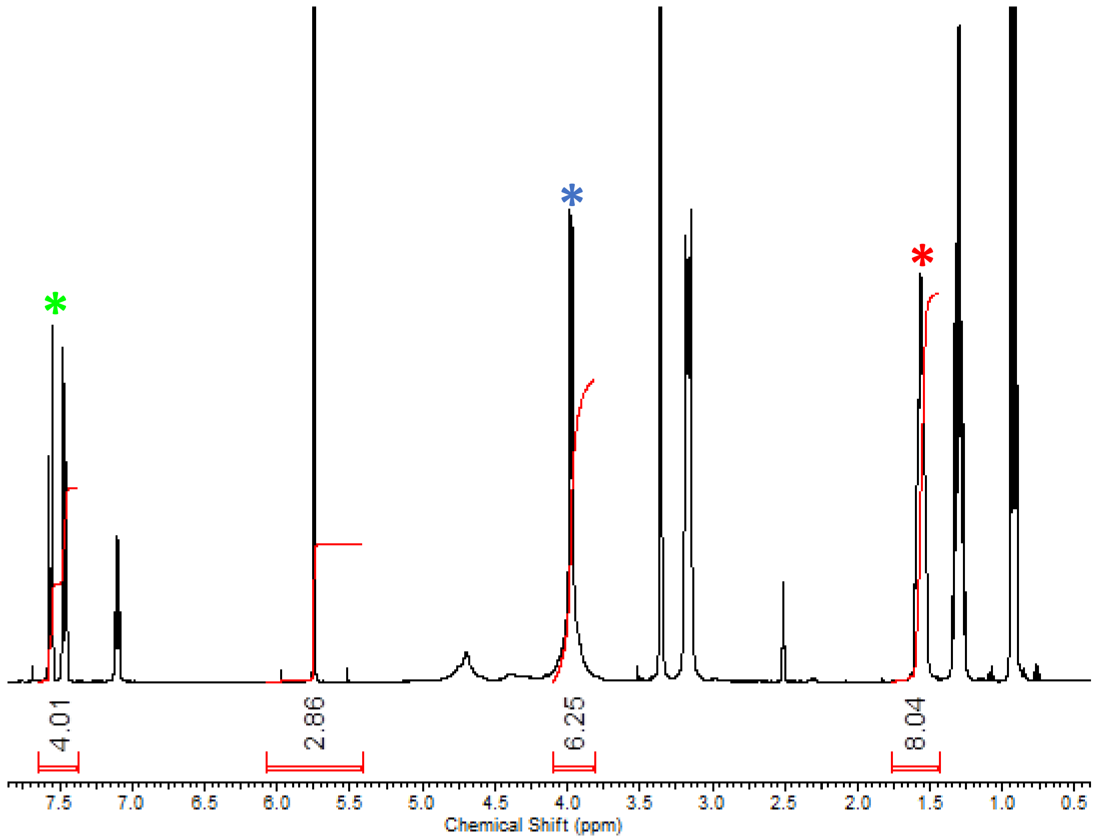

Figure S21 - ^{1}H NMR spectrum with a delay (d_1 = 60 s) of co-formulation **e** (111.8 mM) in DMSO-d_6/ 1.0 % DCM. Comparative integration indicated 0 % of sample has become NMR silent (anionic component of SSA*, TBA countercation* and cisplatin*).

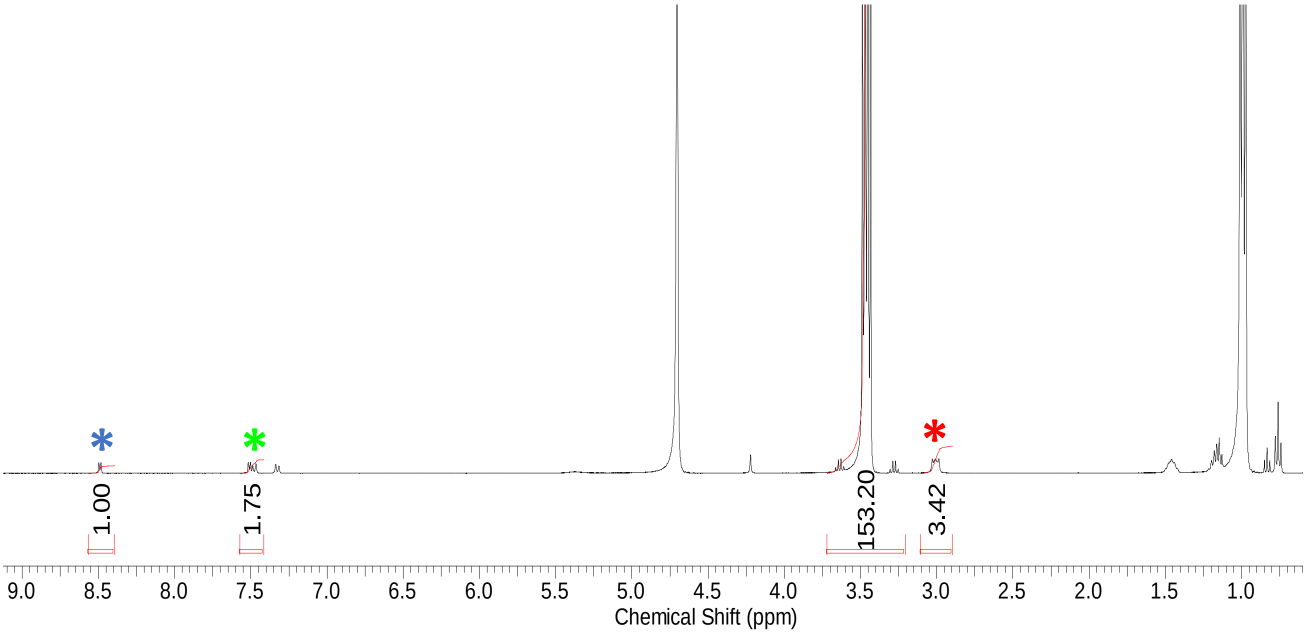

Figure S22 - ^{1}H NMR spectrum with a delay (d_1 = 60 s) of co-formulation **a** (11.2 mM) in D_2O/ 5.0 % EtOH. Comparative integration indicated 56 % of the anionic component of the SSA, 57 % of TBA countercation and 50 % of the isoniazid has become NMR silent. (Isoniazid*, anionic component of the SSA*, TBA countercation*).

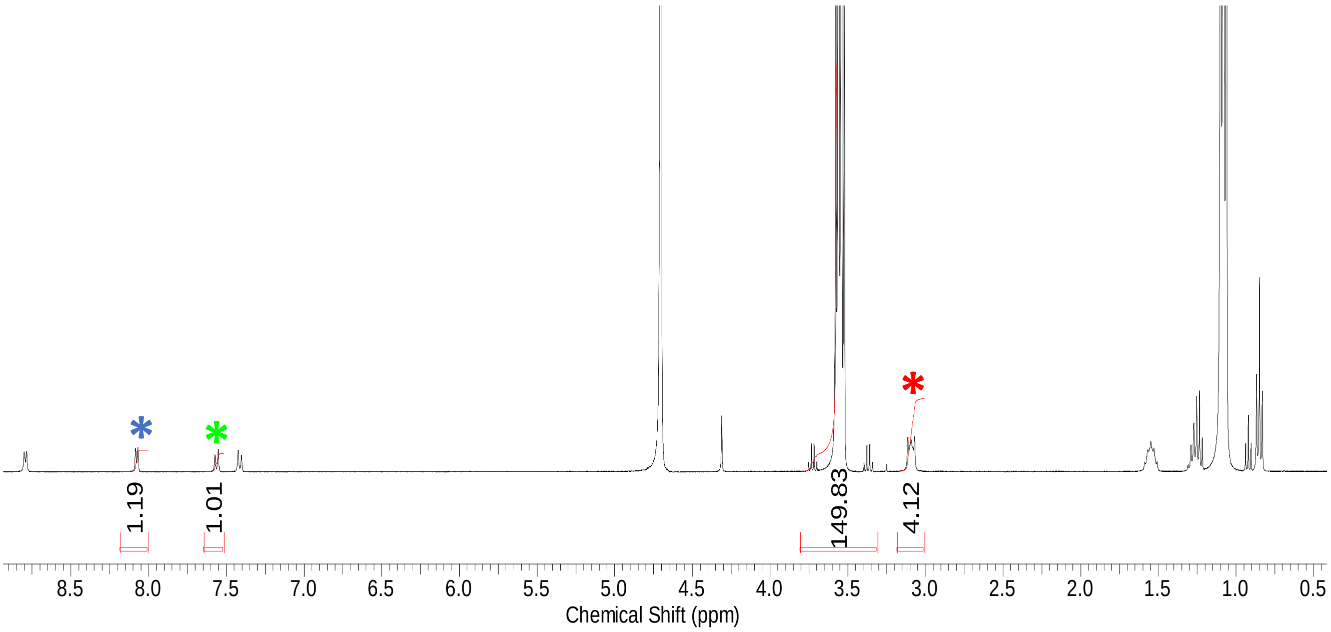

Figure S23 - ^{1}H NMR spectrum with a delay (d_1 = 60 s) of co-formulation **b** (11.4 mM) in D_2O/ 5.0 % EtOH. Comparative integration indicated 50 % of the anionic component of the SSA, 49 % of TBA countercation and 41 % of the isoniazid has become NMR silent. (Isoniazid hydrogen chloride*, anionic component of the SSA*, TBA countercation*).

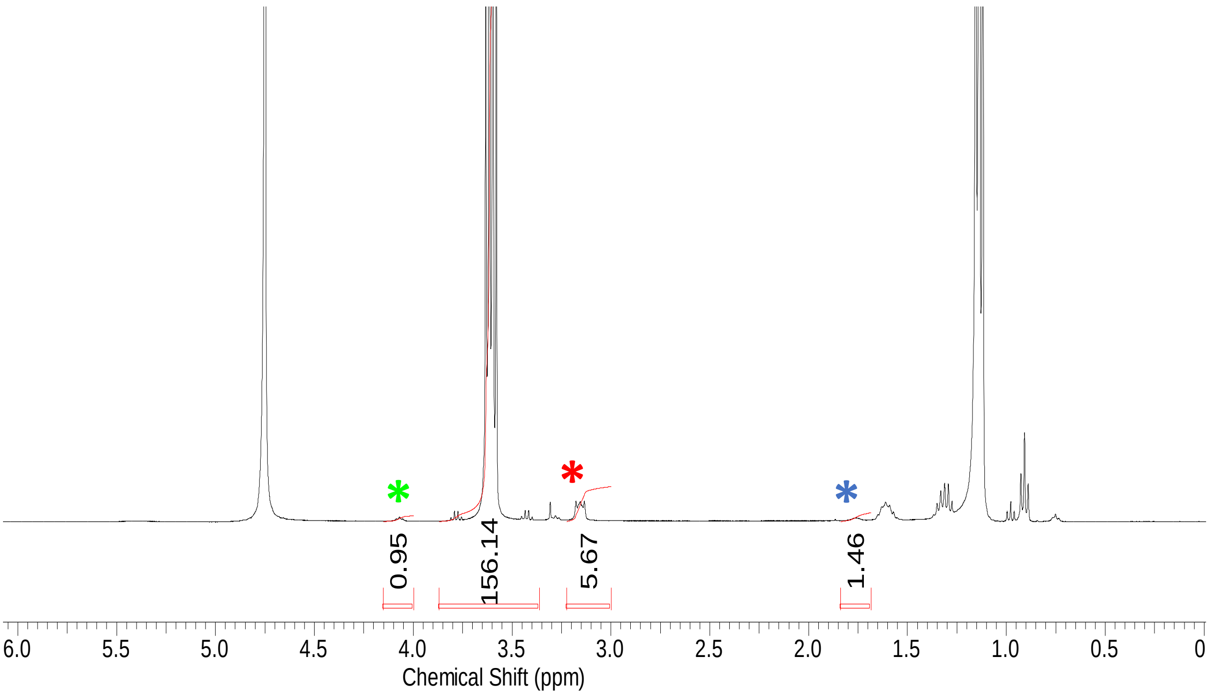

Figure S24 - ^{1}H NMR spectrum with a delay (d_1 = 60 s) of co-formulation **c** (11.0 mM) in D_2O/ 5.0 % EtOH. Comparative integration indicated 53 % of the anionic component of the SSA, 29 % of TBA countercation and 64 % of the octenidine dihydrochloride has become NMR silent. (Octenidine dihydrochloride*, anionic component of the SSA*, TBA countercation*).

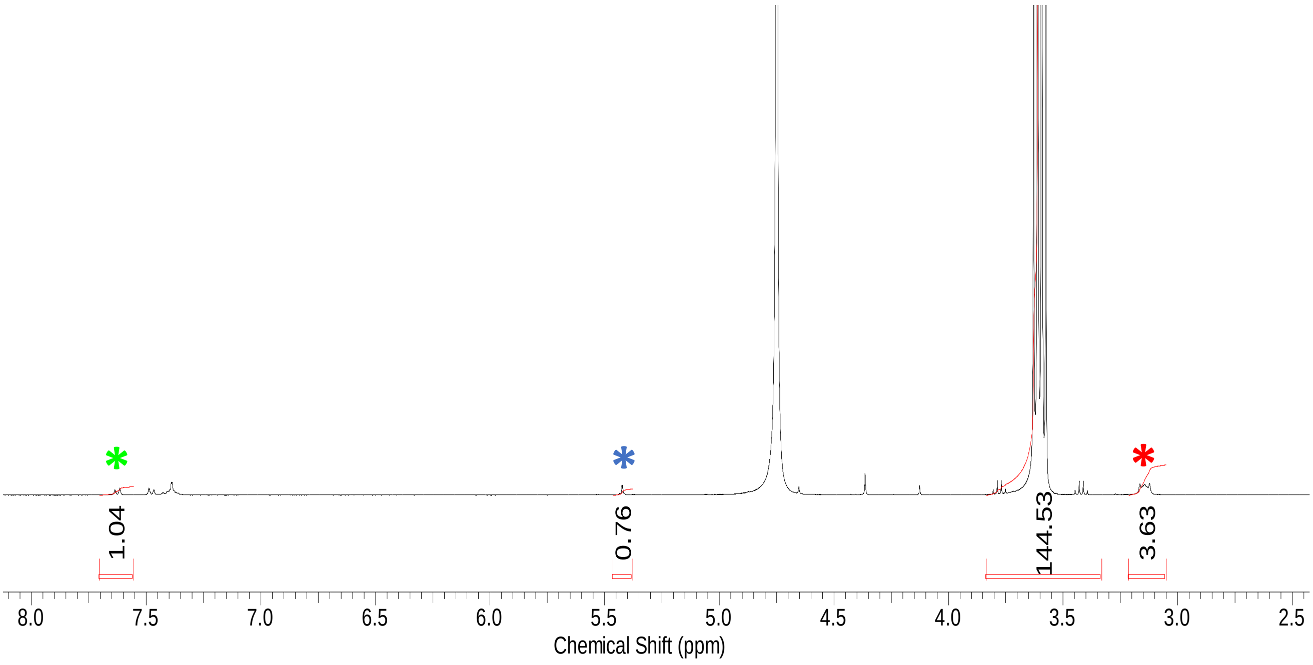

Figure S25 - ^{1}H NMR spectrum with a delay (d_1 = 60 s) of co-formulation **d** (11.9 mM) in D_2O/ 5.0 % EtOH. Comparative integration indicated 48 % of the anionic component of the SSA, 55 % of TBA countercation and 62 % of the octenidine dihydrochloride has become NMR silent. (Ampicillin*, anionic component of the SSA*, TBA countercation*).

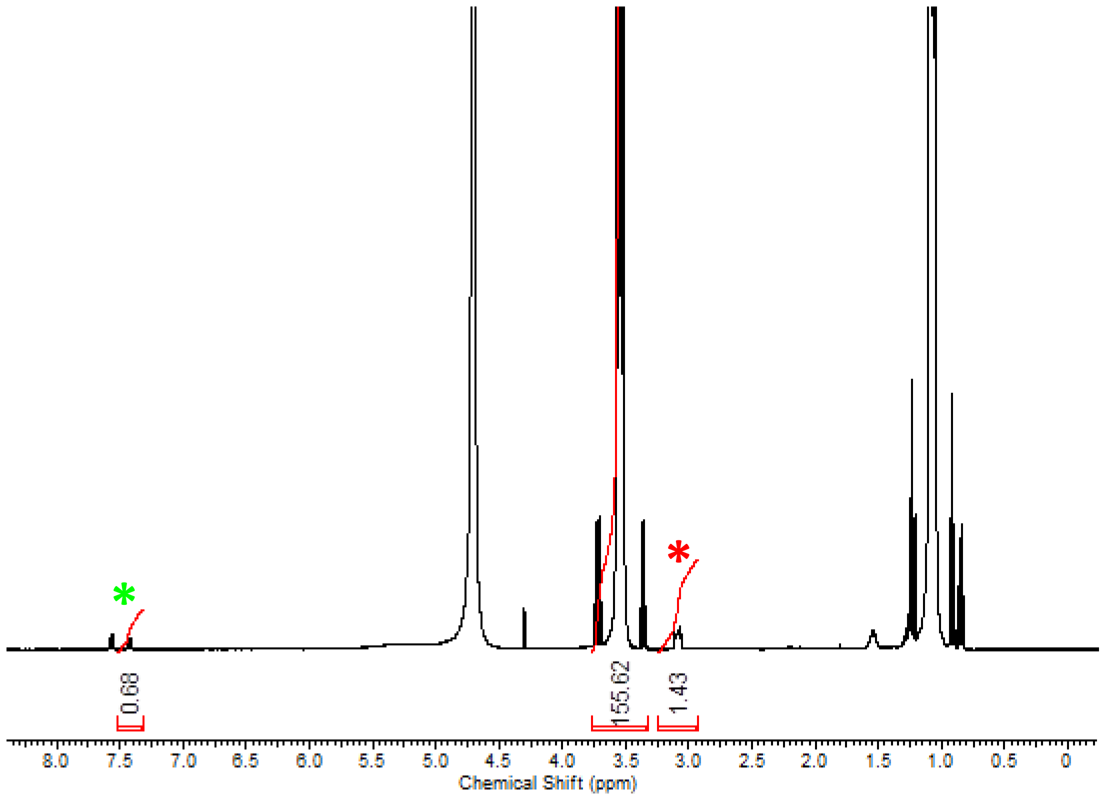

Figure S26 - ^{1}H NMR spectrum with a delay (d_1 = 60 s) of co-formulation **e** (11.0 mM) in D_2O/ 5.0 % EtOH. Comparative integration indicated 65 % of the anionic component of the SSA and 83 % of TBA counteraction has become NMR silent. (Anionic component of the SSA* and TBA countercation*).

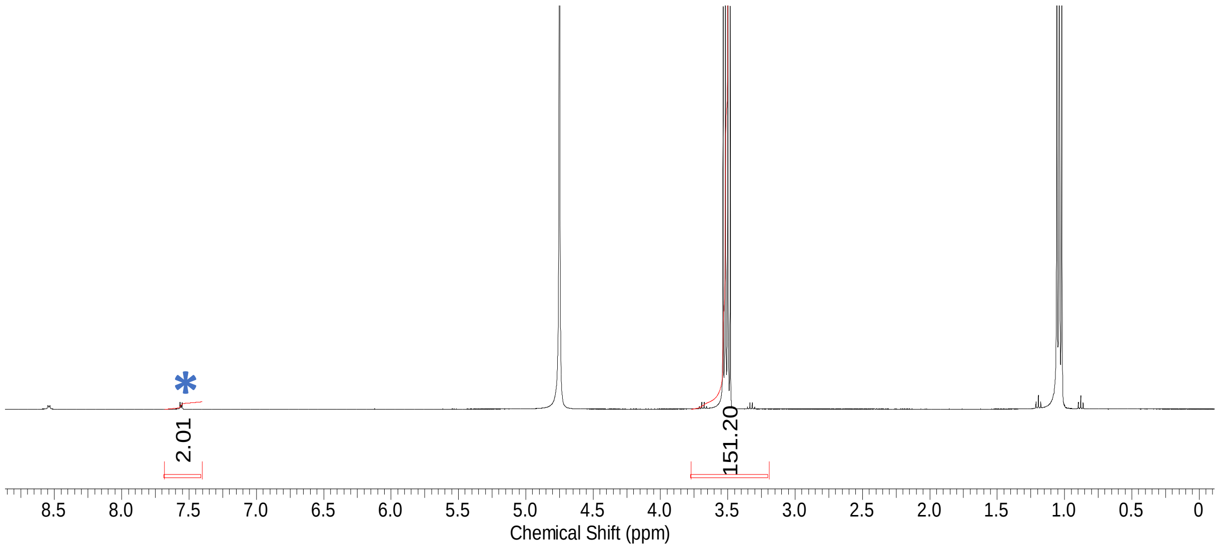

Figure S27 - ^{1}H NMR spectrum with a delay (d_1 = 60 s) of co-formulant **2**, isoniazid (11.3 mM) in D_2O/ 5.0 % EtOH. Comparative integration indicated 0 % of the isoniazid has become NMR silent. (Isoniazid, **2***).

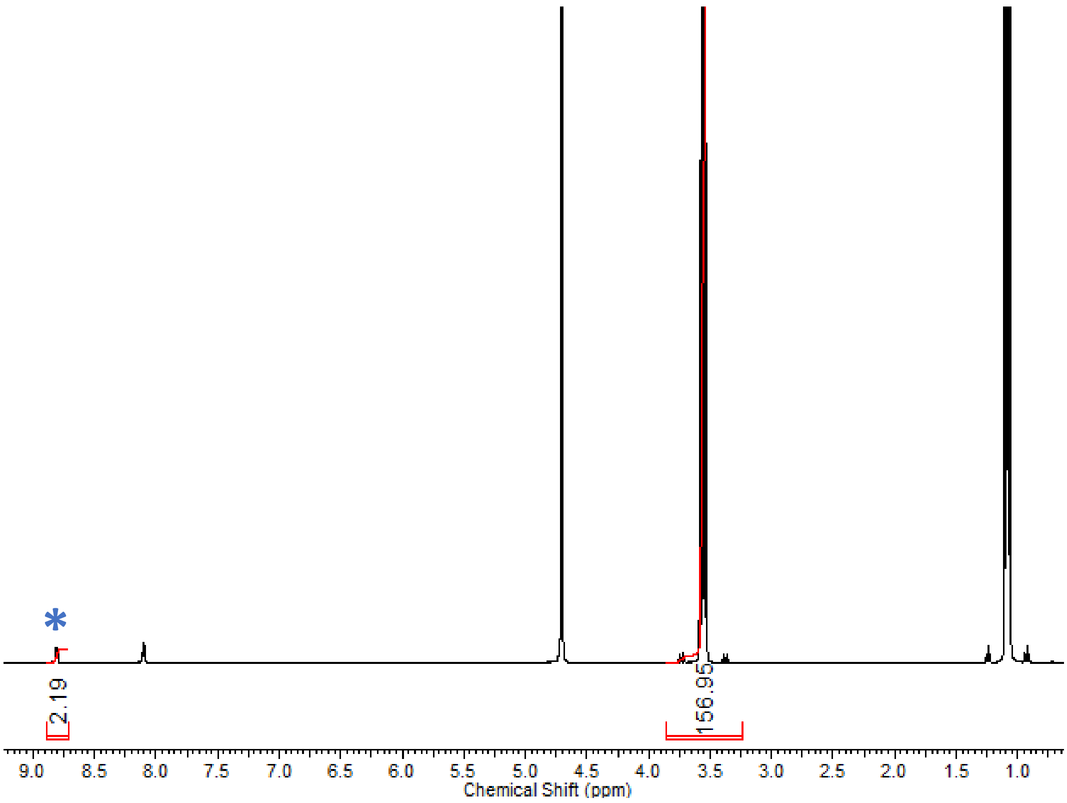

Figure S28 -^{1}H NMR spectrum with a delay (d_1 = 60 s) of co-formulant **3**, isoniazid hydrogen chloride (10.9 mM) in D_2O/ 5.0 % EtOH. Comparative integration indicated 0 % of the isoniazid has become NMR silent. (Isoniazid hydrogen chloride, **3***).

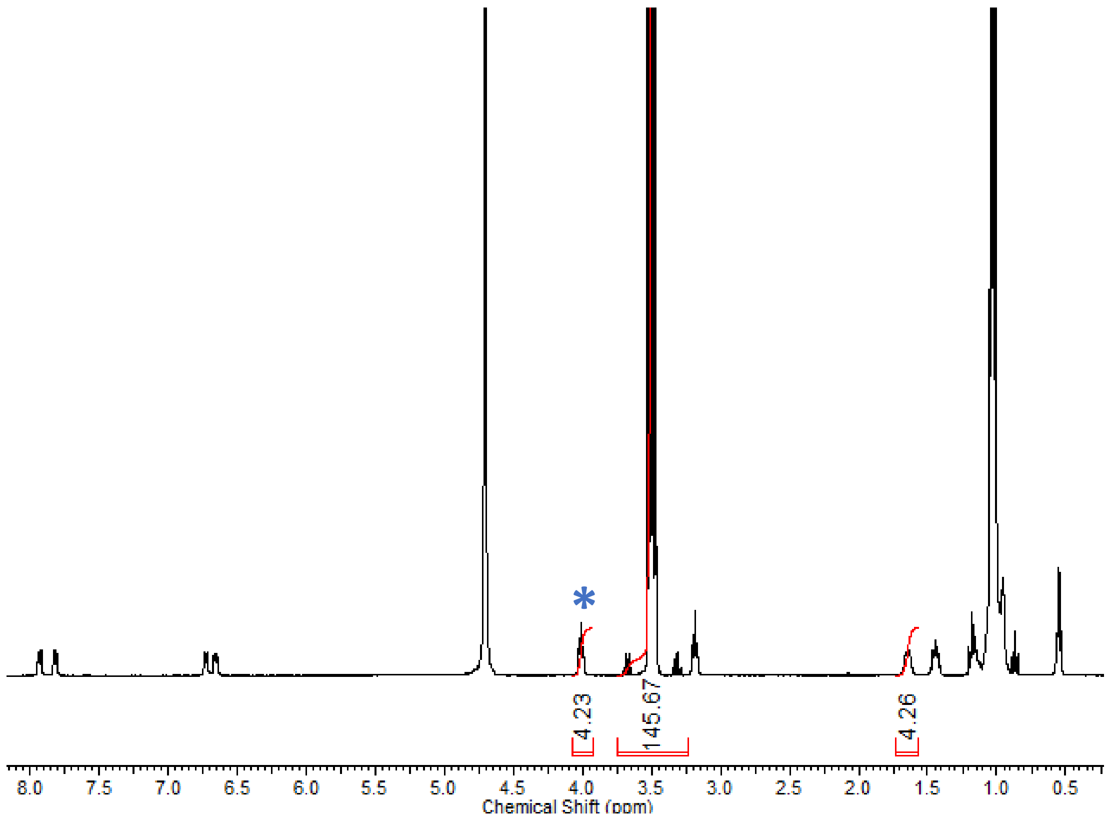

Figure S29 - ^{1}H NMR spectrum with a delay (d_1 = 60 s) of co-formulant **4**, octenidine (11.8 mM) in D_2O/ 5.0 % EtOH. Comparative integration indicated 0 % of the octenidine has become NMR silent. (Octenidine, **4***).

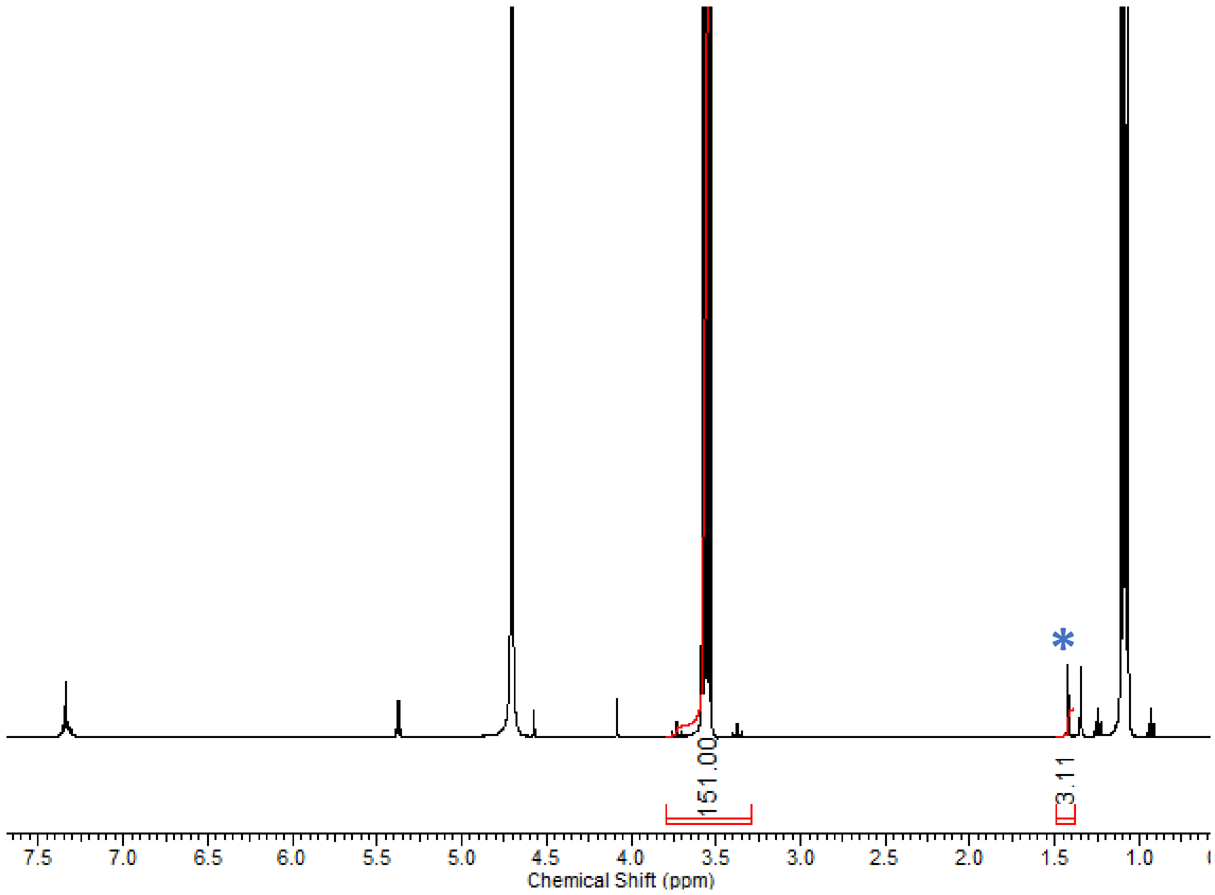

Figure S30 - ^{1}H NMR spectrum with a delay (d_1 = 60 s) of co-formulant **5**, ampicillin (11.4 mM) in D_2O/ 5.0 % EtOH. Comparative integration indicated 0 % of the ampicillin has become NMR silent. (Ampicillin, **5***).

Overview

Table 3 – Overview of the results from quantitative ^{1}H NMR studies obtained from i) DMSO-d_6, standardised with 1.0 % DCM at 112 mM and; ii) D_2O standardised with 5.0 % ethanol at 5.56 mM. Values given in % represent the observed proportion of compound to become NMR silent. All quantitative ^{1}H NMR experiments were conducted with a delay time (d_1) of 60 s at 298 K.

Co-formulation	Solvent system	Anion	Cation	Co-formulant
**1** only[5]	DMSO-d_6	0	0	n/a
	D_2O	51	50	n/a
**a**	DMSO-d_6	0	0	0
	D_2O	56	57	50
**b**	DMSO-d_6	0	0	0
	D_2O	50	49	41
**c**	DMSO-d_6	0	0	0
	D_2O	53	29	64
**d**	DMSO-d_6	0	0	0
	D_2O	48	55	62
**e**	DMSO-d_6	0	0	0
	D_2O	65	83	*a*

a = could not be determined due to peak overlap.

^{1}H NMR self-association studies

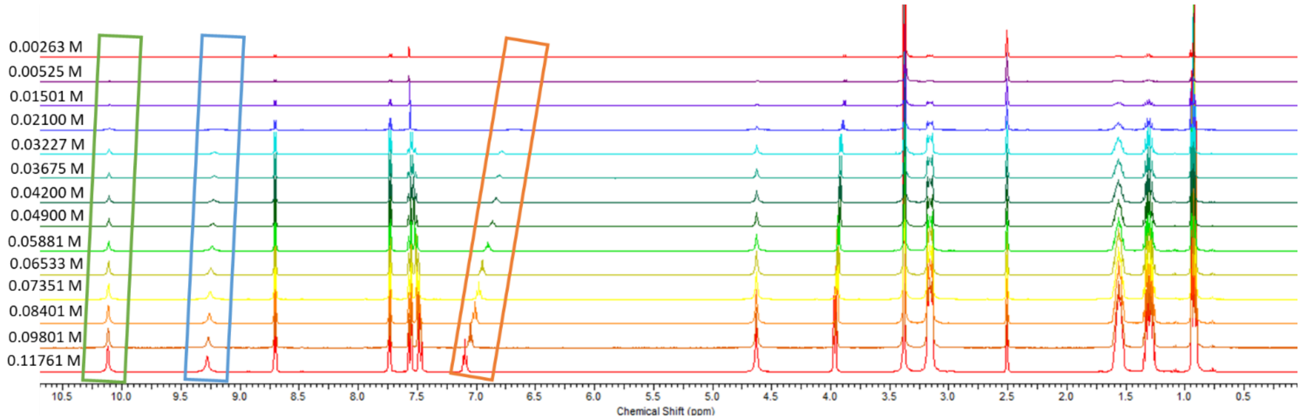

Figure S31 - ^{1}H NMR stack plot of co-formulation **a** in DMSO-d_6 0.5 % H_2O solution. Samples were prepared in series with an aliquot of the most concentrated solution undergoing serial dilution.

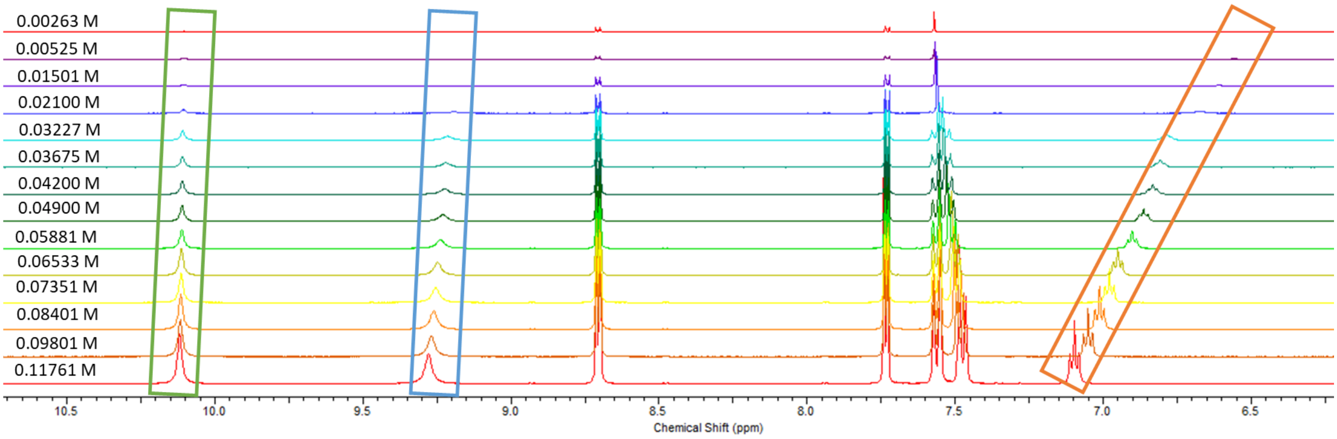

Figure S32 - Enlarged ^{1}H NMR stack plot of co-formulation **a** in DMSO-d_6 0.5 % H_2O solution. Samples were prepared in series with an aliquot of the most concentrated solution undergoing serial dilution.

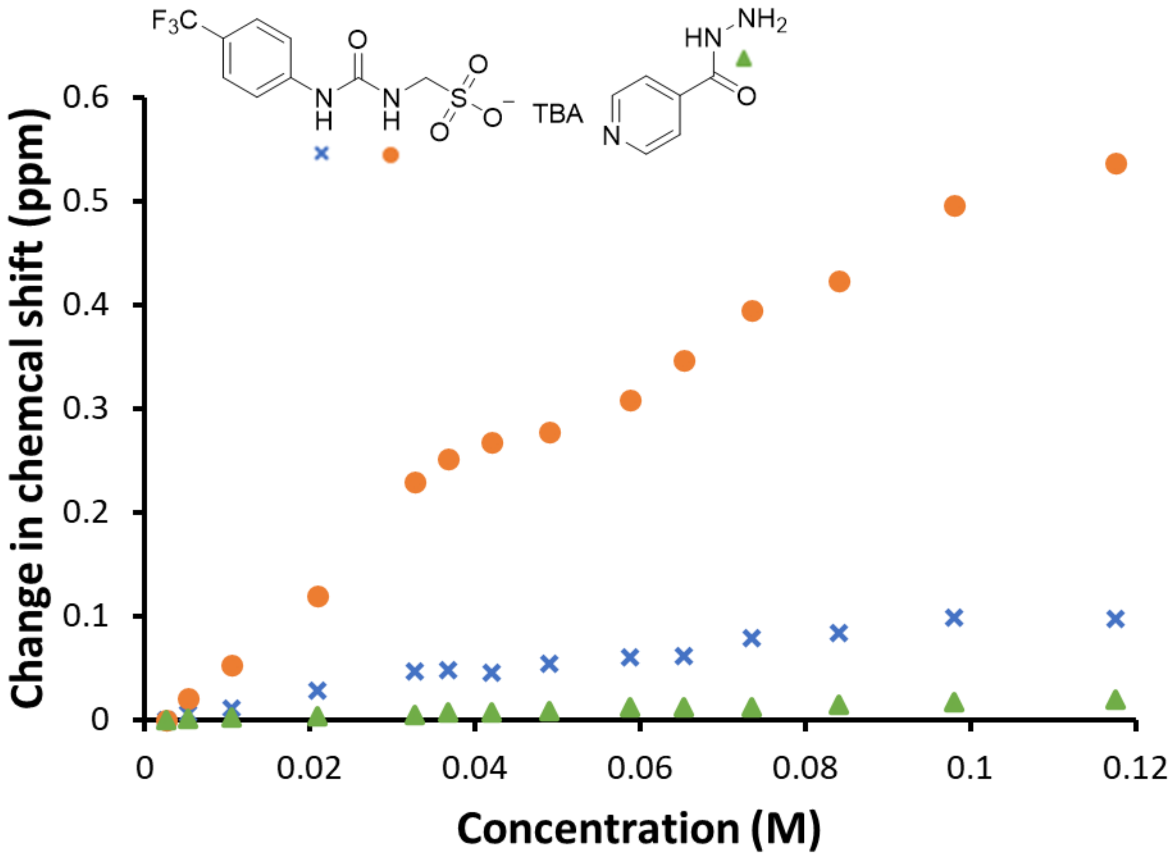

Figure S33 - Graph illustrating the ^{1}H NMR down-field change in chemical shift of SSA (**1**) urea and appropriate co-formulant NH resonances with increasing concentration of co-formulation **a** in DMSO-d_6 0.5 % H_2O (298 K).

Self-association constant calculation

Co-formulation **a** - Dilution study in DMSO-d_6 0.5 % H_2O. Values calculated from data gathered from SSA (1) NH urea resonances.

Equal K/Dimerization model

K_e = 4.18 M^{-1} ± 2.9347 % K_{dim} = 2.09 M^{-1} ± 1.4673 %

http://app.supramolecular.org/bindfit/view/af60fbcd-6906-4279-8651-4a153e3f9d7e

CoEK model

K_e = 1.88 M^{-1} ± 36.2950 % K_{dim} = 0.94 M^{-1} ± 18.1475 %   ρ = 1.70 ± 45.4394 %

http://app.supramolecular.org/bindfit/view/8483344b-5f77-4661-adff-f15e4b0f2e8a

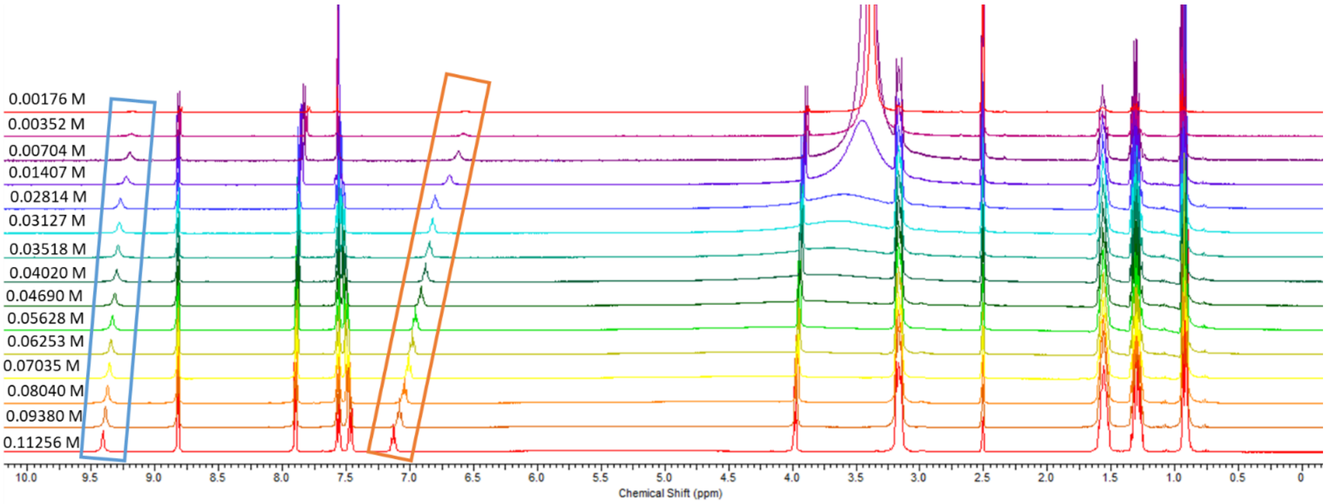

Figure S34 - ^{1}H NMR stack plot of co-formulation **b** in DMSO-d_6 0.5 % H_2O solution. Samples were prepared in series with an aliquot of the most concentrated solution undergoing serial dilution.

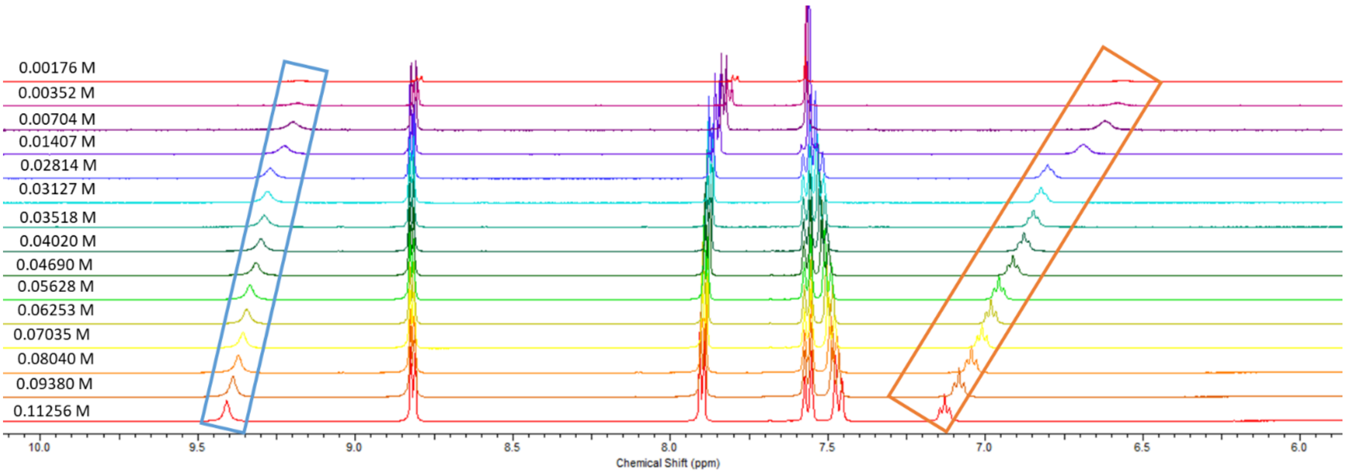

Figure S35 - Enlarged ^{1}H NMR stack plot of co-formulation **b** in DMSO-d_6 0.5 % H_2O solution. Samples were prepared in series with an aliquot of the most concentrated solution undergoing serial dilution.

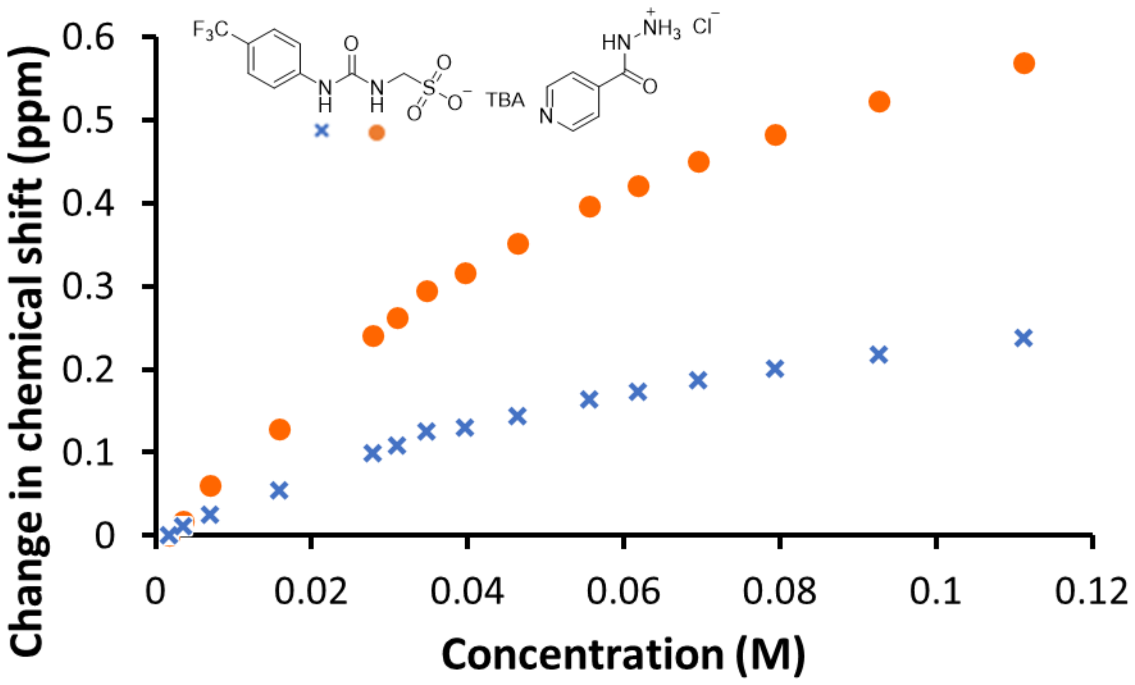

Figure S36 - Graph illustrating the ^{1}H NMR down-field change in chemical shift of SSA (**1**) urea resonances with increasing concentration of co-formulation **b** in DMSO-d_6 0.5 % H_2O (298 K).

Self-association constant calculation

Co-formulation **b** - Dilution study in DMSO-d_6 0.5 % H_2O. Values calculated from data gathered from SSA (1) NH urea resonances.

Equal K/Dimerization model

K_e = 8.73 M^{-1} ± 0.6136 % K_{dim} = 4.37 M^{-1} ± 0.3068 %

http://app.supramolecular.org/bindfit/view/72564cd3-8777-42c1-8286-91afc97421e7

CoEK model

K_e = 13.37 M^{-1} ± 1.1955 % K_{dim} = 6.69 M^{-1} ± 0.5978 %   ρ = 0.70 ± 3.4165 %

http://app.supramolecular.org/bindfit/view/2512868c-efd5-40cf-afee-14431c8f1db7

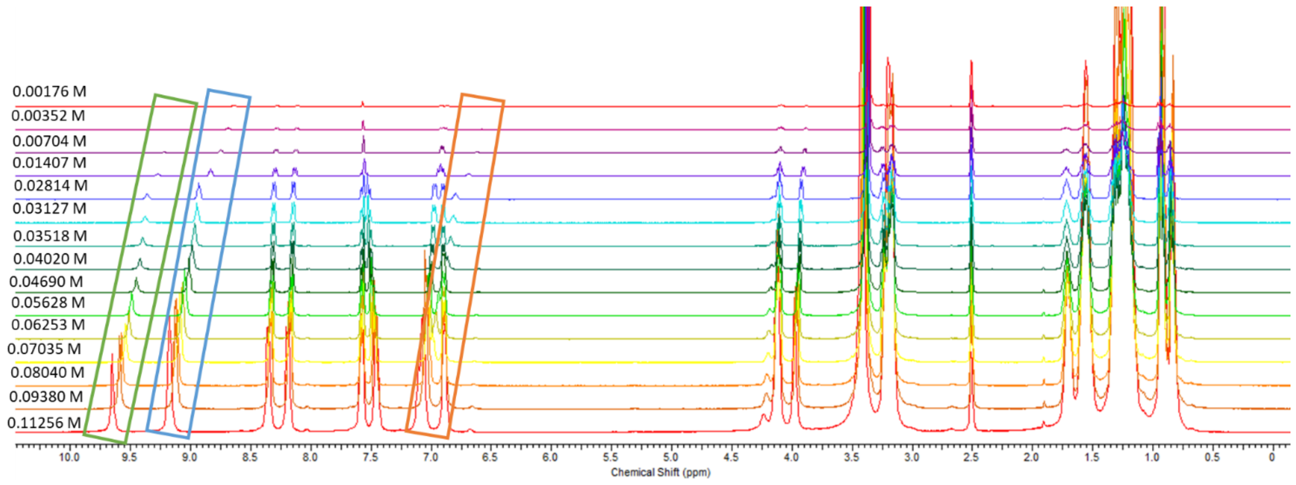

Figure S37 - ^{1}H NMR stack plot of co-formulation **c** in DMSO-d_6 0.5 % H_2O solution. Samples were prepared in series with an aliquot of the most concentrated solution undergoing serial dilution.

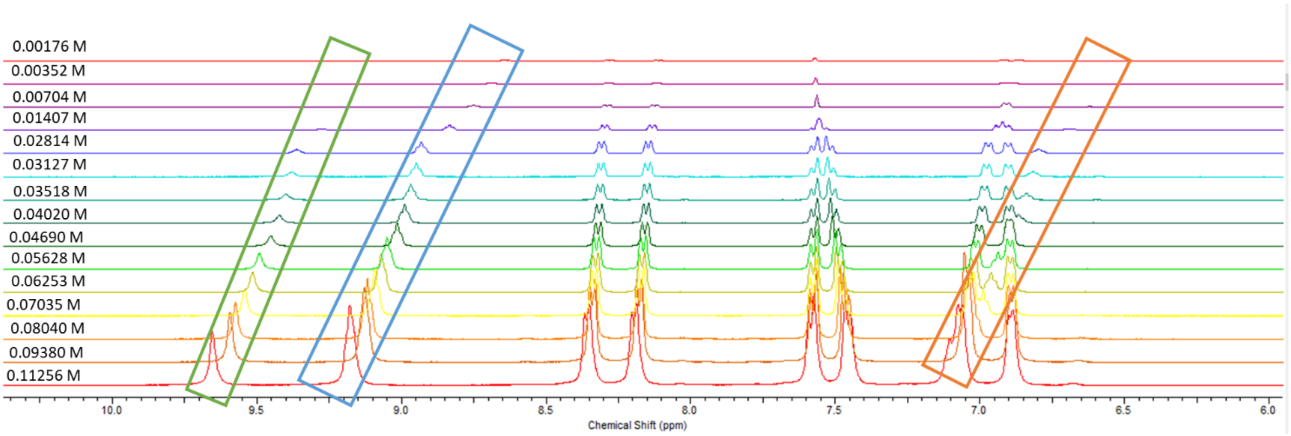

Figure S38 -Enlarged ^{1}H NMR stack plot of co-formulation **c** in DMSO-d_6 0.5 % H_2O solution. Samples were prepared in series with an aliquot of the most concentrated solution undergoing serial dilution.

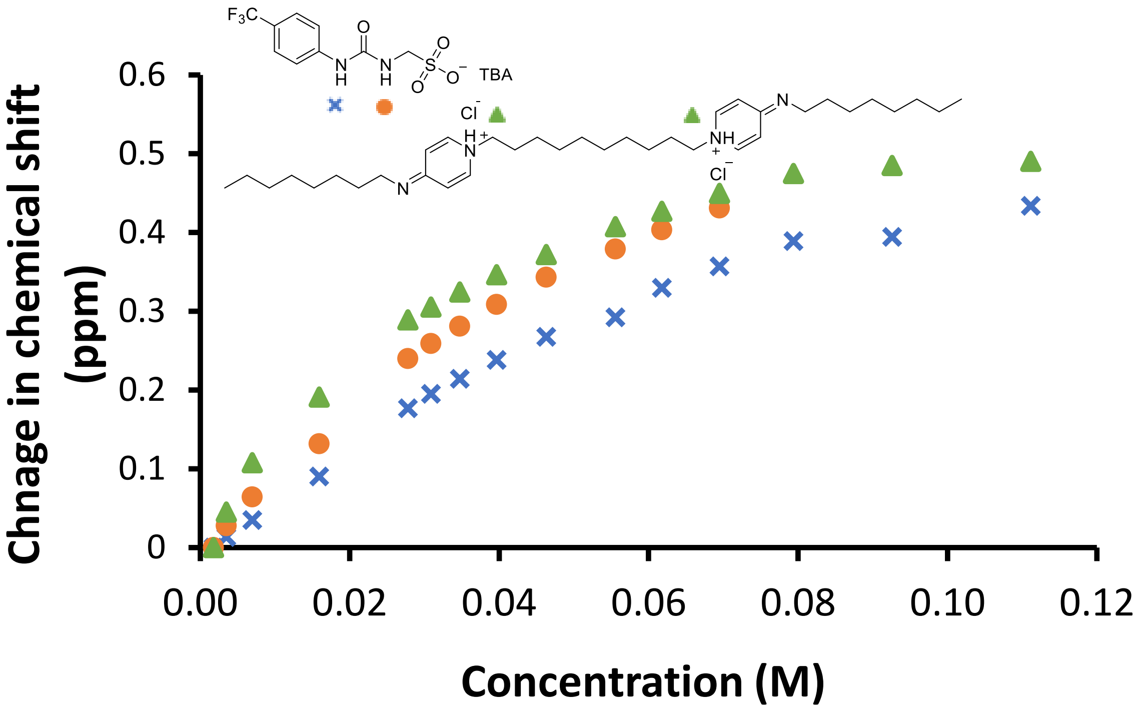

Figure S39 - Graph illustrating the ^{1}H NMR down-field change in chemical shift of SSA (**1**) urea and appropriate co-formulant NH resonances with increasing concentration of co-formulation **c** in DMSO-d_6 0.5 % H_2O (298 K).

Self-association constant calculation

Co-formulation **c** - Dilution study in DMSO-d_6 0.5 % H_2O. Values calculated from data gathered from SSA (1) NH urea resonance.

Equal K/Dimerization model

K_e = 5.85 M^{-1} ± 1.5916 % K_{dim} = 2.92 M^{-1} 0.7958 %

http://app.supramolecular.org/bindfit/view/e15303e8-eb56-4dca-b782-c28be7611357

CoEK model

K_e = 11.30 M^{-1} ± 3.9459 % K_{dim} = 5.65 M^{-1} ± 1.9730 % ρ = 0.60 ± 10.5806 %

http://app.supramolecular.org/bindfit/view/dcaf8e9c-6662-4c84-b1e6-3981726939d

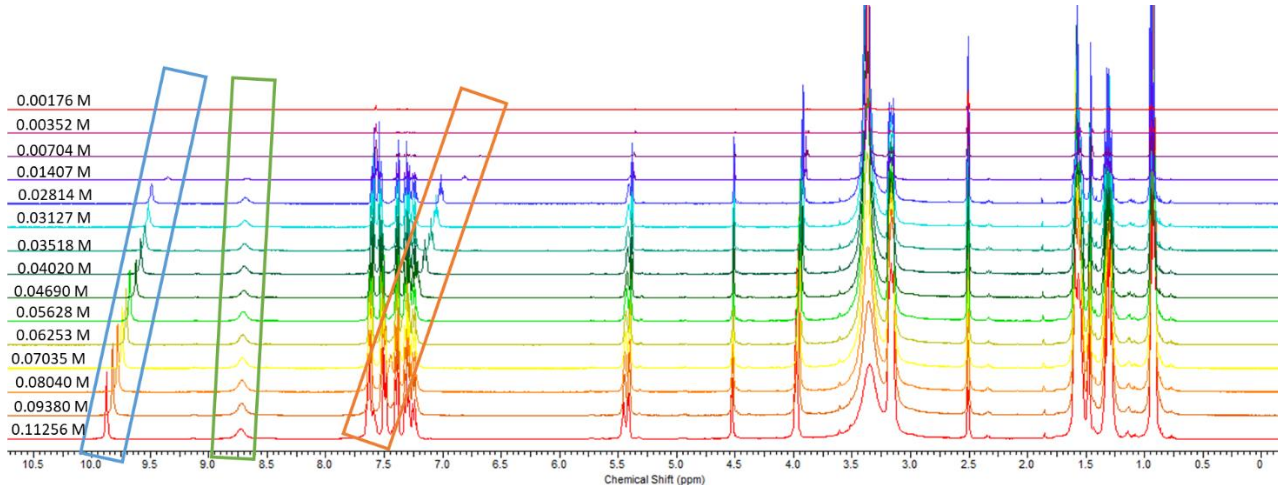

Figure S40 - ^{1}H NMR stack plot of co-formulation **d** in DMSO-d_6 0.5 % H_2O solution. Samples were prepared in series with an aliquot of the most concentrated solution undergoing serial dilution.

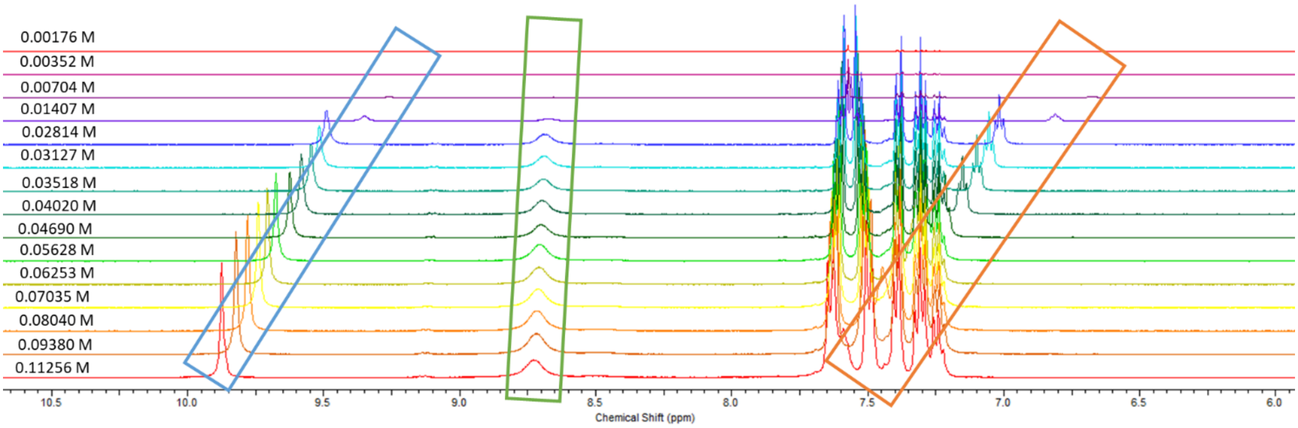

Figure S41 - Enlarged ^{1}H NMR stack plot of co-formulation **d** in DMSO-d_6 0.5 % H_2O solution. Samples were prepared in series with an aliquot of the most concentrated solution undergoing serial dilution.

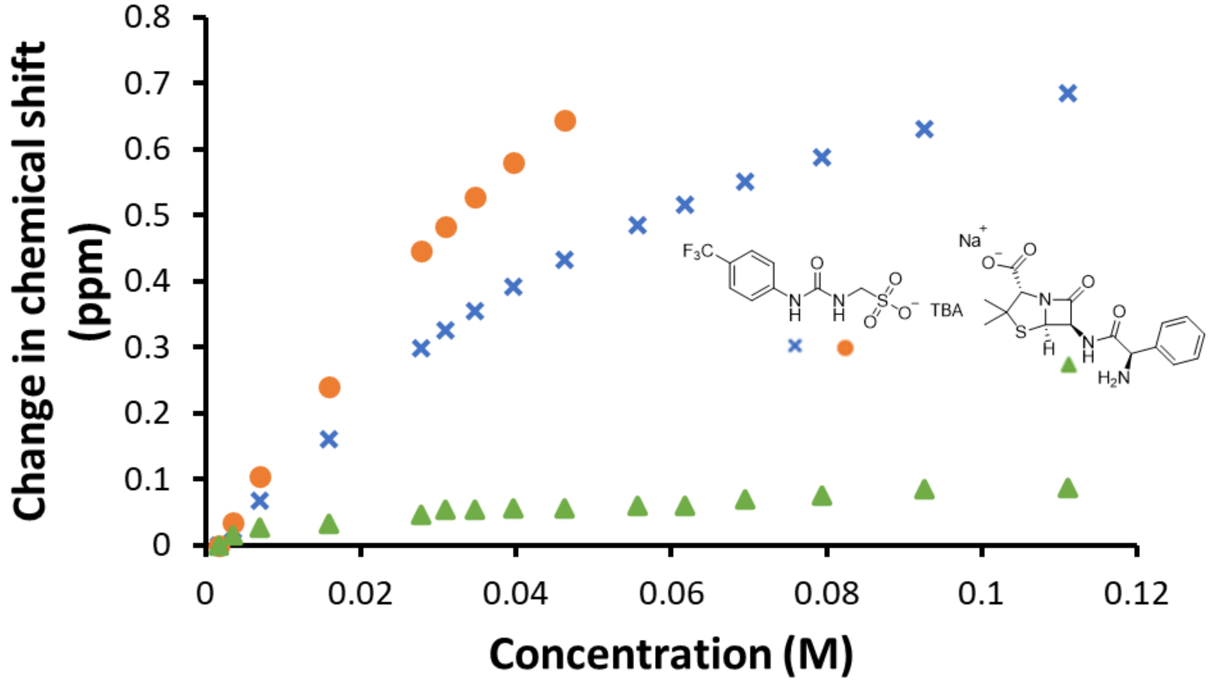

Figure S42 - Graph illustrating the ^{1}H NMR down-field change in chemical shift of SSA (**1**) urea and appropriate co-formulant NH resonances with increasing concentration of co-formulation **d** in DMSO-d_6 0.5 % H_2O (298 K).

Self-association constant calculation

Co-formulation **d** - Dilution study in DMSO-d_6 0.5 % H_2O. Values calculated from data gathered from SSA (1) NH urea resonance.

Equal K/Dimerization model

K_e = 9.81 M^{-1} ± 0.9962 % K_{dim} = 4.91 M^{-1} 0.4981 %

http://app.supramolecular.org/bindfit/view/2865831b-c5e4-4eb4-a97d-e3f1a955de0b

CoEK model

K_e = 18.10 M^{-1} ± 0.5718 % K_{dim} = 9.05 M^{-1} ± 0.2859 % ρ = 0.55 ± 2.0789 %

http://app.supramolecular.org/bindfit/view/b5e2838a-37d6-40a4-bee9-e033afb6bba2

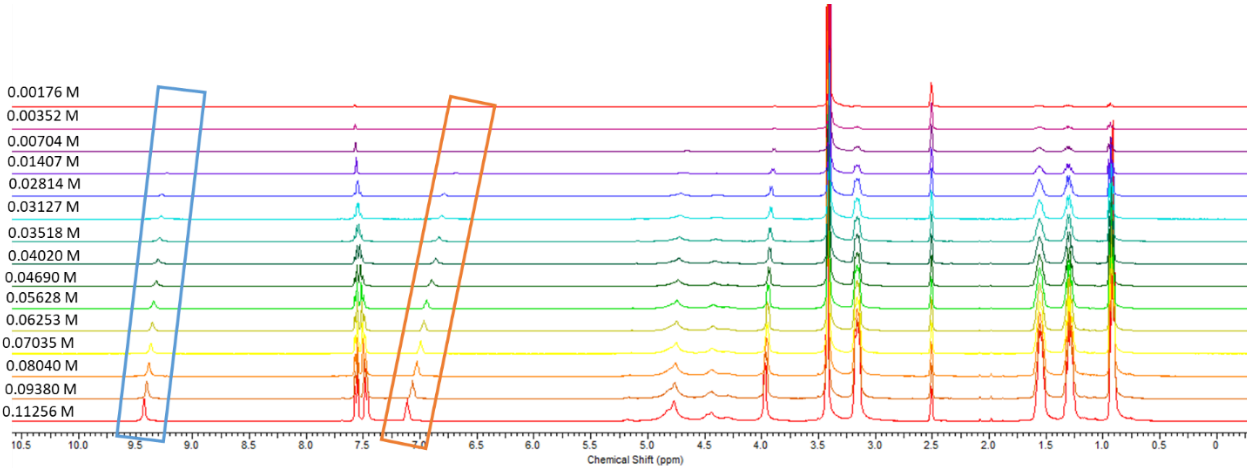

Figure S43 - ^{1}H NMR stack plot of co-formulation **e** in DMSO-d_6 0.5 % H_2O solution. Samples were prepared in series with an aliquot of the most concentrated solution undergoing serial dilution.

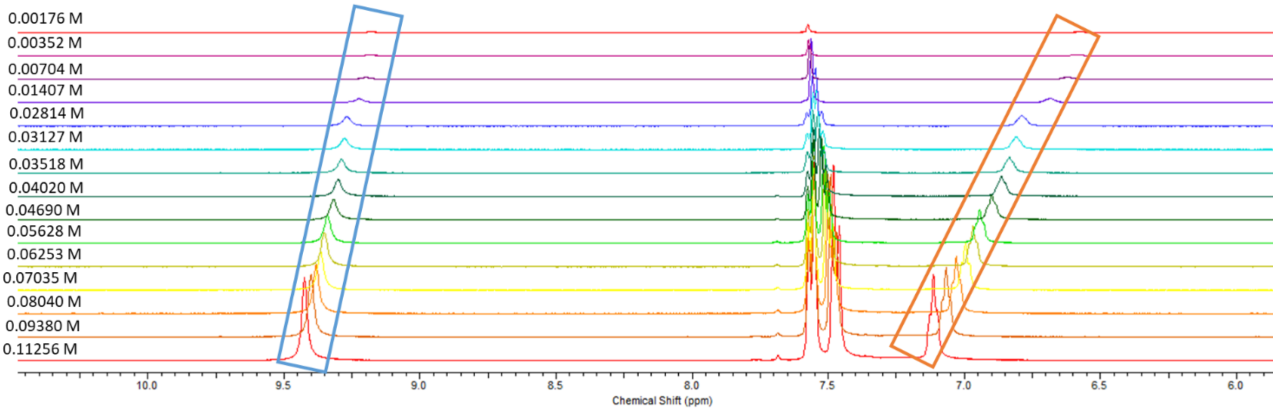

Figure S44 -Enlarged ^{1}H NMR stack plot of co-formulation **e** in DMSO-d_6 0.5 % H_2O solution. Samples were prepared in series with an aliquot of the most concentrated solution undergoing serial dilution.

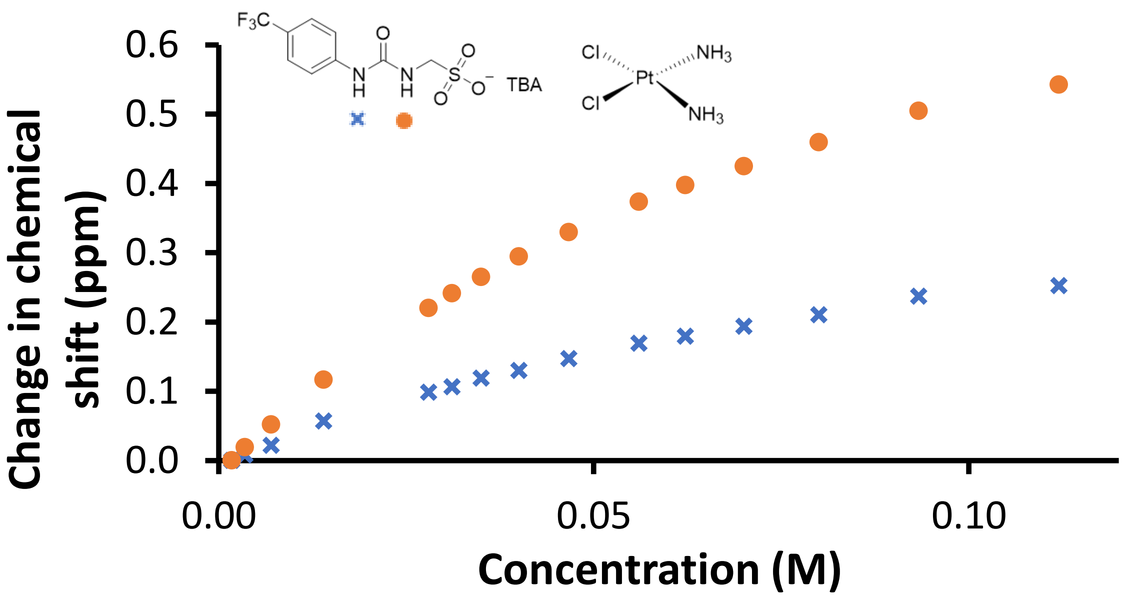

Figure S45 - Graph illustrating the ^{1}H NMR down-field change in chemical shift of SSA (**1**) urea NH resonances with increasing concentration of co-formulation **e** in DMSO-d_6 0.5 % H_2O (298 K).

Self-association constant calculation

Co-formulation **e** - Dilution study in DMSO-d_6 0.5 % H_2O. Values calculated from data gathered from SSA (1) NH urea resonances.

Equal K/Dimerization model

K_e = 7.34 M^{-1} ± 0.7666 % K_{dim} = 3.67 M^{-1} ± 0.3833 %

http://app.supramolecular.org/bindfit/view/9a4e48ba-c79c-48cf-8ef9-70df96f1595e

CoEK model

K_e = 13.11 M^{-1} ± 1.5699 % K_{dim} = 6.55 M^{-1} ± 0.7849 % ρ = 0.62 ± 4.4228 %

http://app.supramolecular.org/bindfit/view/a686415f-cefd-4e16-b9e8-3670179f4e3a

Overview

Table S4 – Summary of self-association constants calculated from dilution studies in DMSO-d_6 0.5 % H_2O

	EK model (M^{-1})		CoEK (M^{-1})		
	K_e	K_{dim}	K_e	K_{dim}	ρ
**Control**[5]	5.3 ± 0.6 %	2.7 ± 0.3 %	13.0 ± 0.7 %	6.5 ± 0.4 %	0.5 ± 2.1 %
**a**	4.18 M^{-1} ± 2.9 %	2.09 M^{-1} ± 1.5 %	1.88 M^{-1} ± 36.3 %	0.94 M^{-1} ± 18.1 %	1.70 ± 45.4 %
**b**	8.73 M^{-1} ± 0.6 %	4.37 M^{-1} ± 0.3 %	13.37 M^{-1} ± 1.2 %	6.69 M^{-1} ± 0.6 %	0.70 ± 3.4 %
**c**	5.85 M^{-1} ± 1.6 %	2.92 M^{-1} 0.8 %	11.30 M^{-1} ± 4.0 %	5.65 M^{-1} ± 2.0 %	0.60 ± 10.6 %
**d**	9.81 M^{-1} ± 1.0 %	4.91 M^{-1} 0.5 %	18.10 M^{-1} ± 0.6 %	9.05 M^{-1} ± 0.3 %	0.55 ± 2.1 %
**e**	7.34 M^{-1} ± 0.76 %	3.67 M^{-1} ± 0.38 %	13.11 M^{-1} ± 1.56 %	6.55 M^{-1} ± 0.78 %	0.62 M^{-1} ± 4.42 %

Dynamic Light Scattering

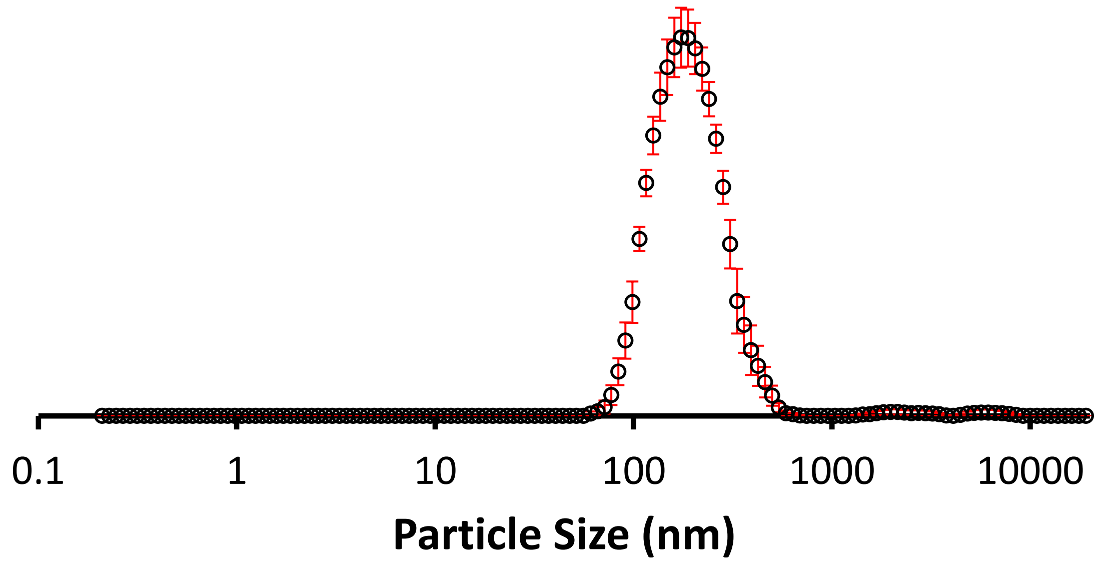

Figure S46 - The average intensity particle size distribution calculated using 9 DLS runs for co-formulation **a** (5.56 mM) in an EtOH: H_2O (1:19) solution at 298 K.

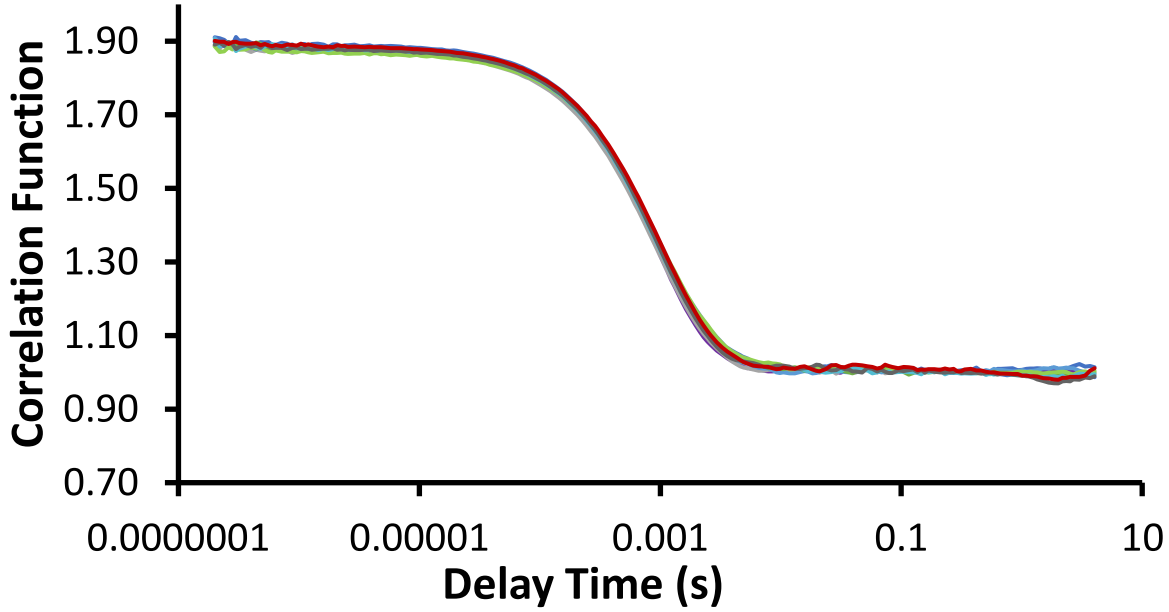

Figure S47 - Correlation function data for 9 DLS runs of co-formulation **a** (5.56 mM) in an EtOH: H_2O (1:19) solution at 298 K.

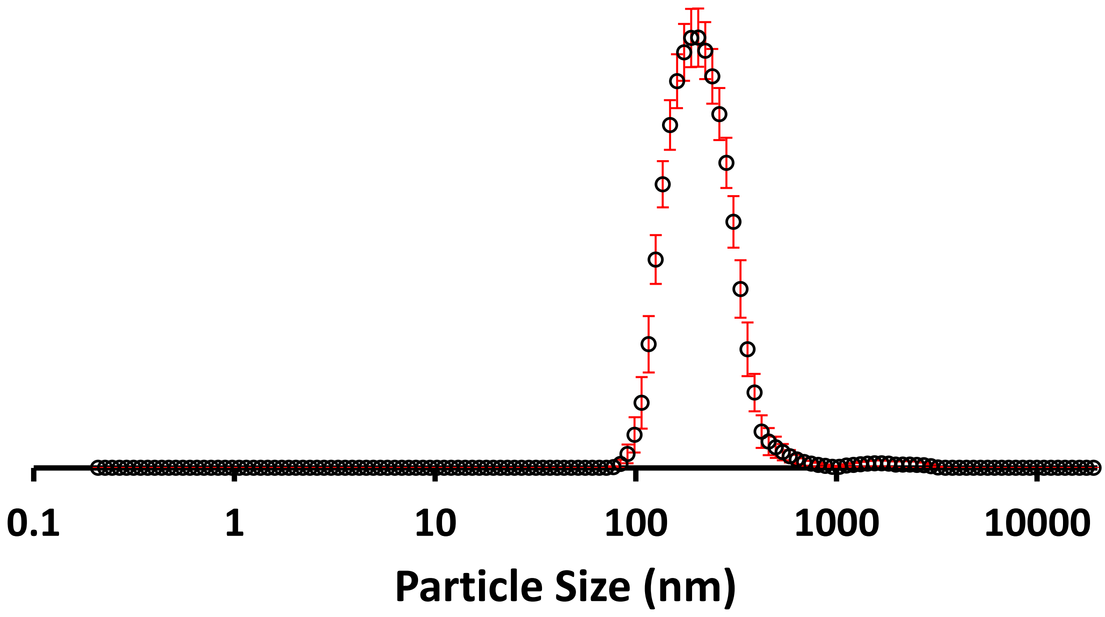

Figure S48 - The average intensity particle size distribution calculated using 9 DLS runs for co-formulation **a** (0.56 mM) in an EtOH: H_2O (1:19) solution at 298 K.

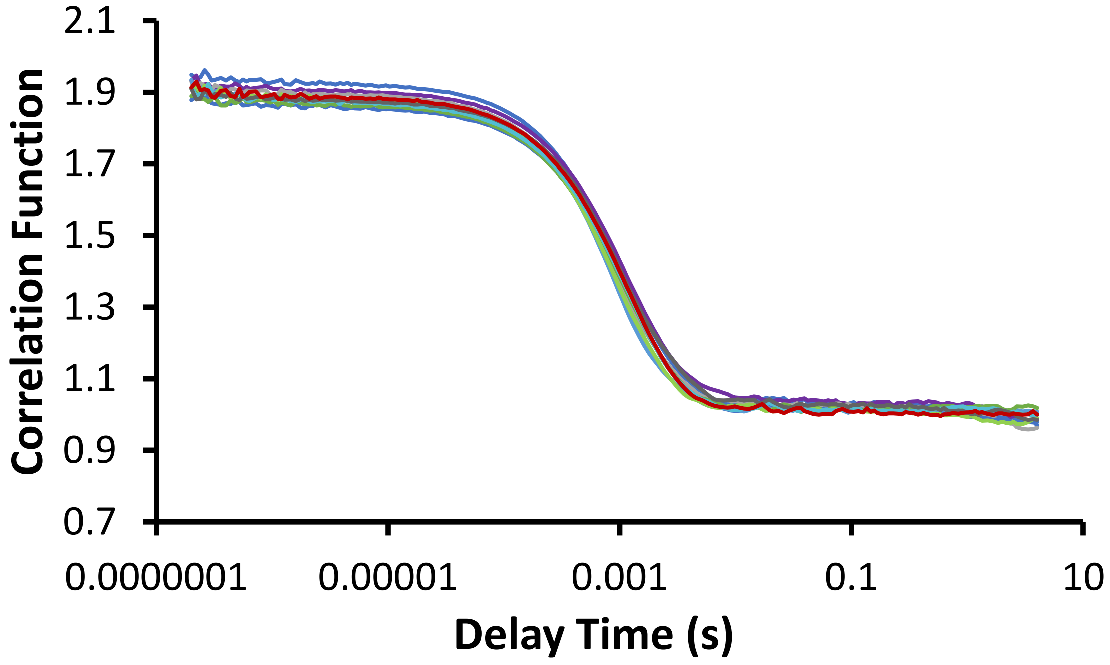

Figure S49 - Correlation function data for 9 DLS runs of co-formulation **a** (0.56 mM) in an EtOH: H_2O (1:19) solution at 298 K.

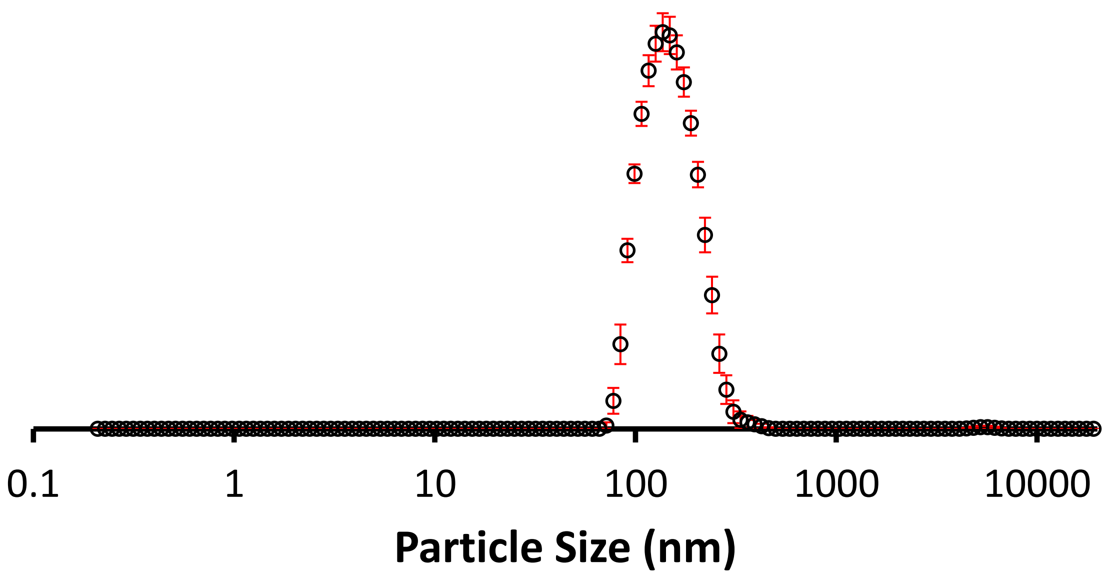

Figure S50 - The average intensity particle size distribution calculated using 9 DLS runs for co-formulation **b** (5.56 mM) in an EtOH: H_2O (1:19) solution at 298 K.

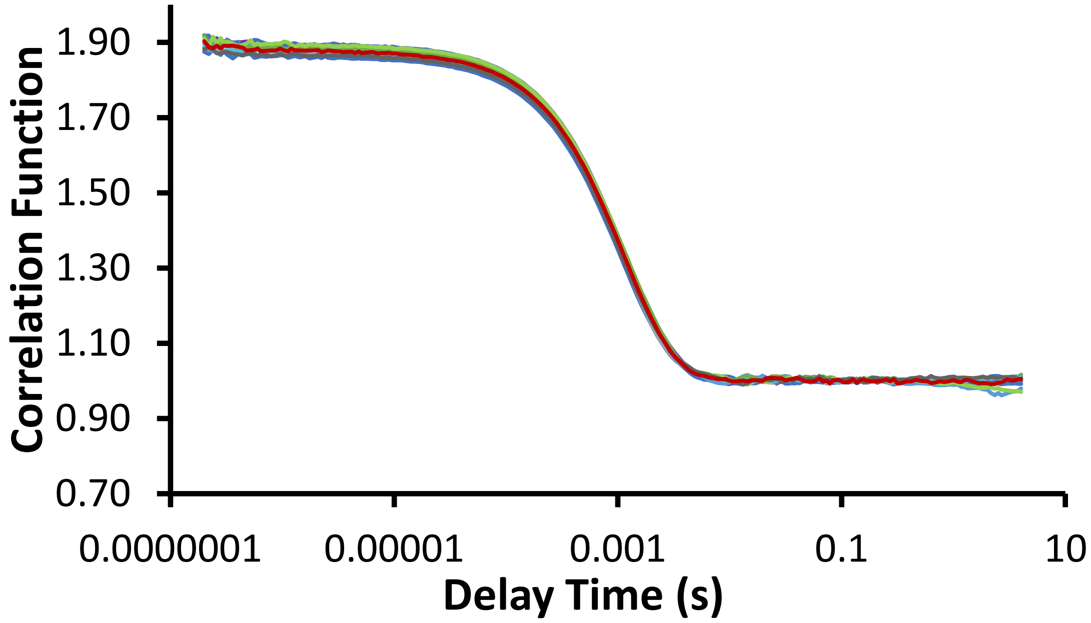

Figure S51 - Correlation function data for 9 DLS runs of co-formulation **b** (5.56 mM) in an EtOH: H_2O (1:19) solution at 298 K.

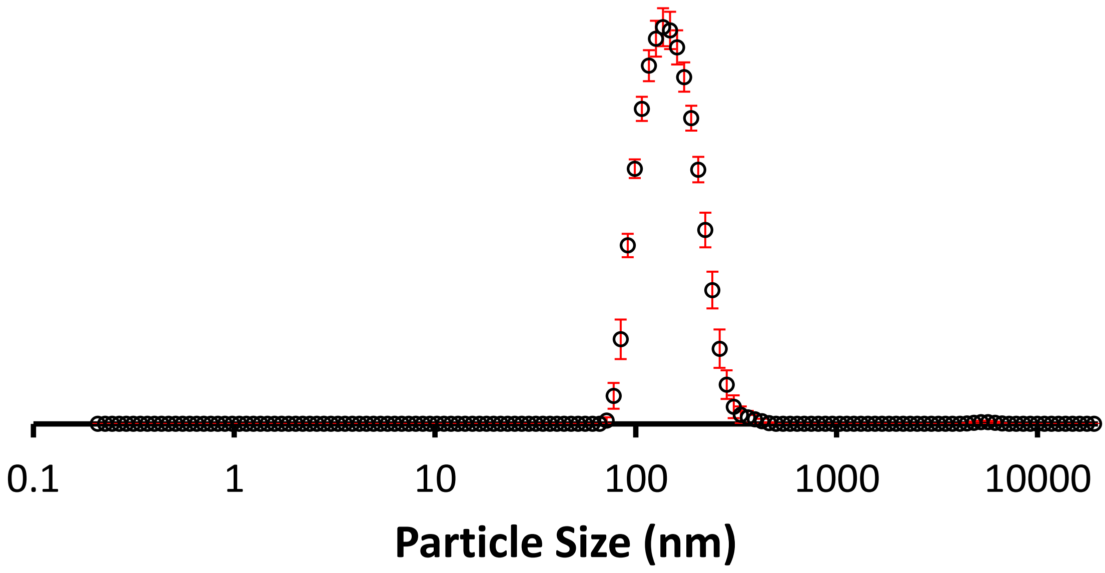

Figure S52 - The average intensity particle size distribution calculated using 9 DLS runs for co-formulation **b** (0.56 mM) in an EtOH: H_2O (1:19) solution at 298 K.

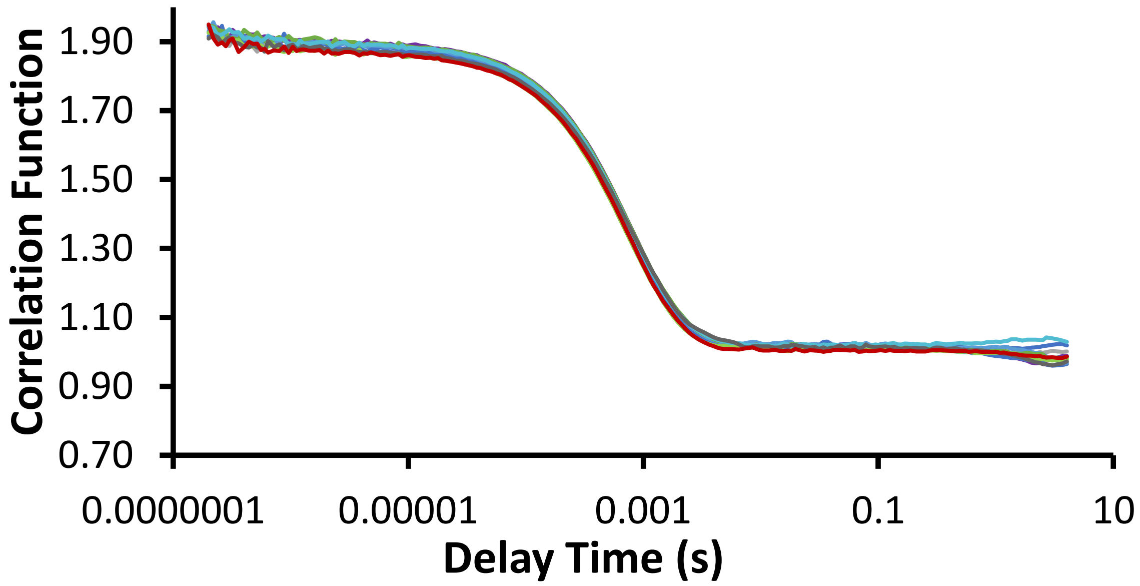

Figure S53 - Correlation function data for 9 DLS runs of co-formulation **b** (0.56 mM) in an EtOH: H_2O (1:19) solution at 298 K.

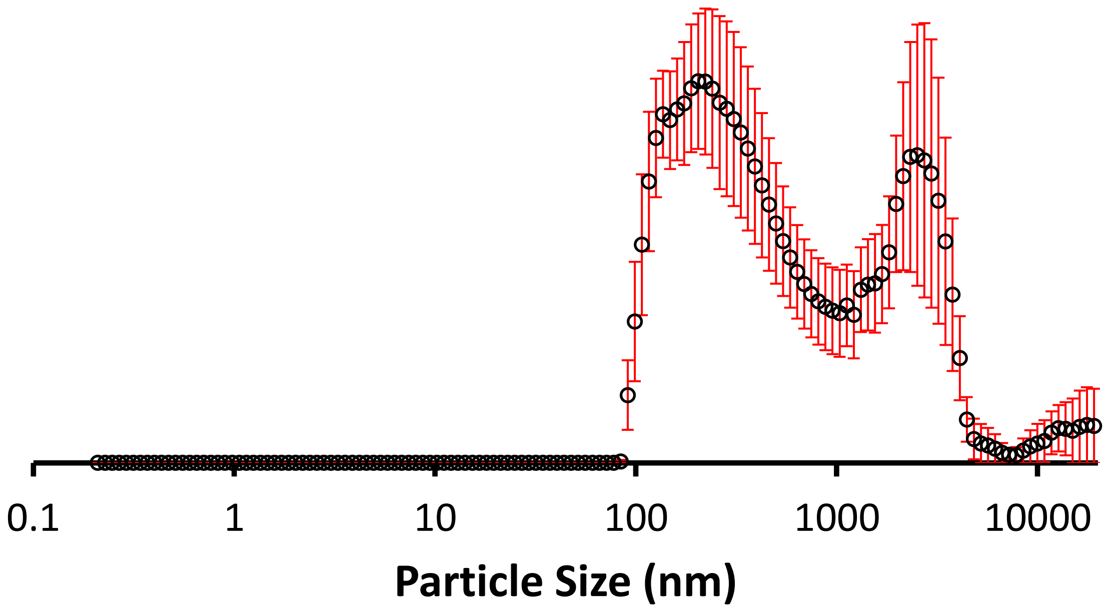

Figure S54 - The average intensity particle size distribution calculated using 9 DLS runs for co-formulation **c** (0.56 mM) in an EtOH: H_2O (1:19) solution at 298 K.

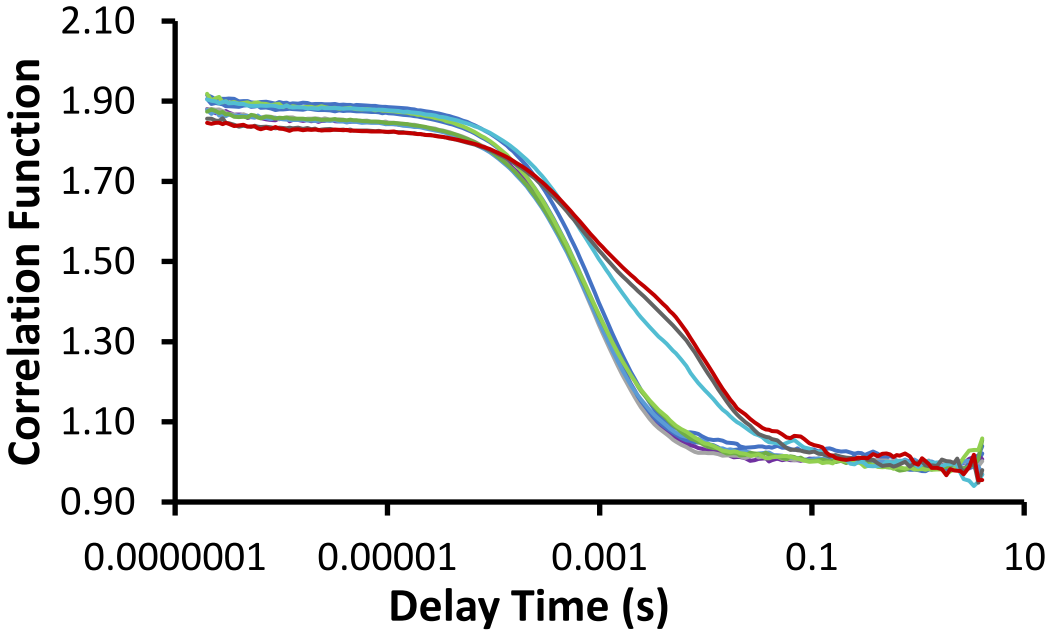

Figure S55 - Correlation function data for 9 DLS runs of co-formulation **c** (0.56 mM) in an EtOH: H_2O (1:19) solution at 298 K.

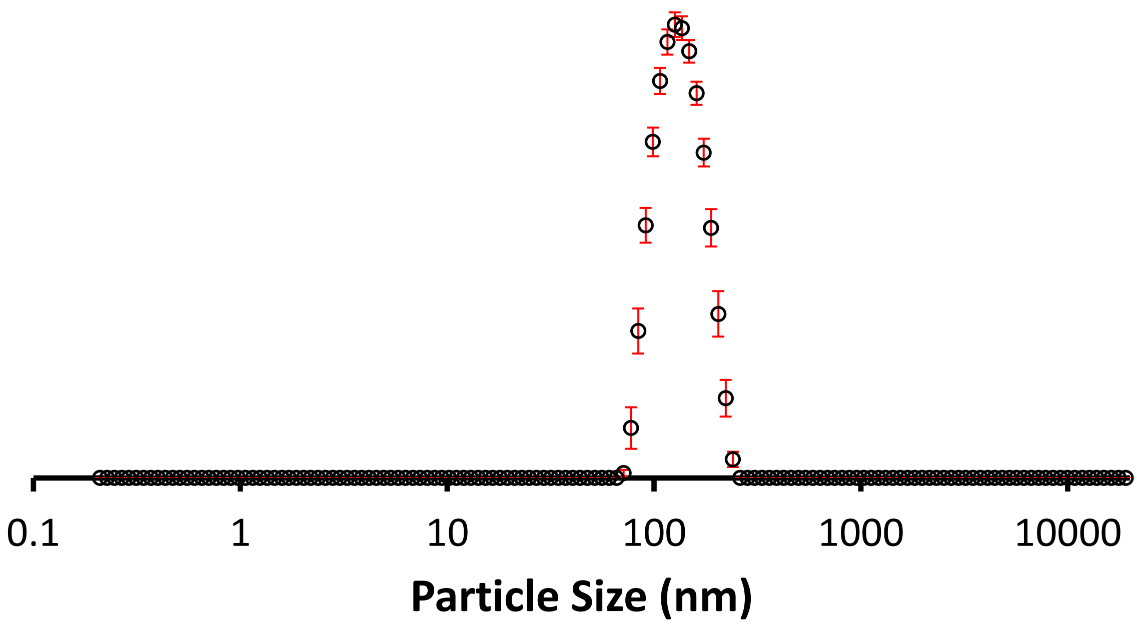

Figure S56 - The average intensity particle size distribution calculated using 9 DLS runs for co-formulation **d** (5.56 mM) in an EtOH: H_2O (1:19) solution at 298 K.

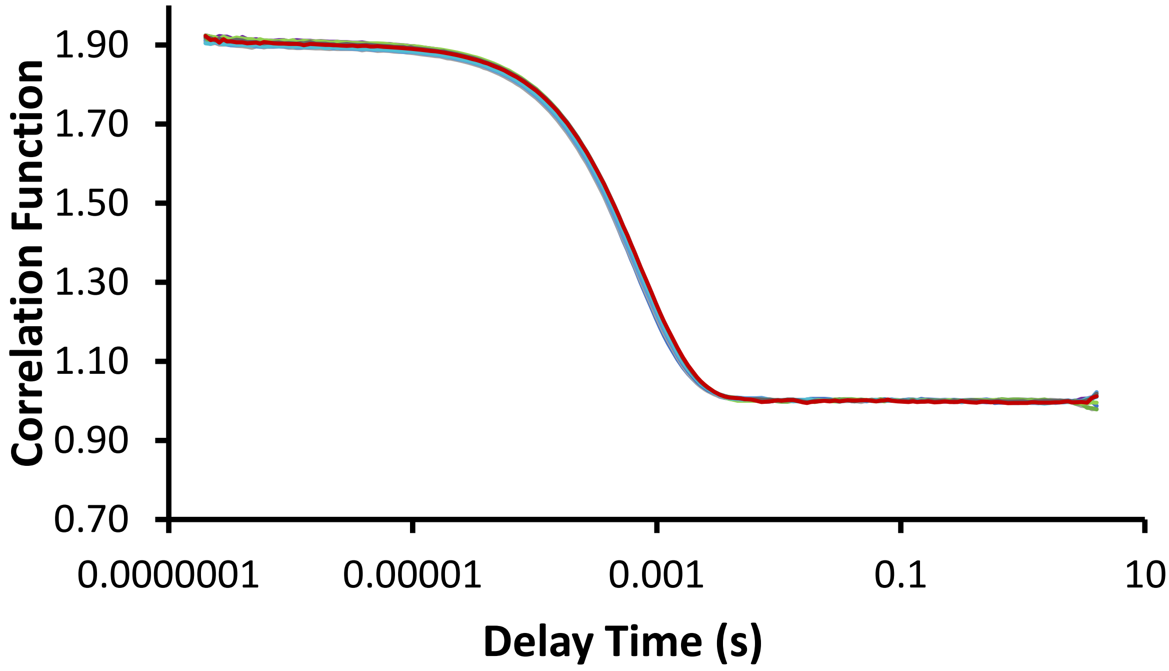

Figure S57 - Correlation function data for 9 DLS runs of co-formulation **d** (5.56 mM) in an EtOH: H_2O (1:19) solution at 298 K.

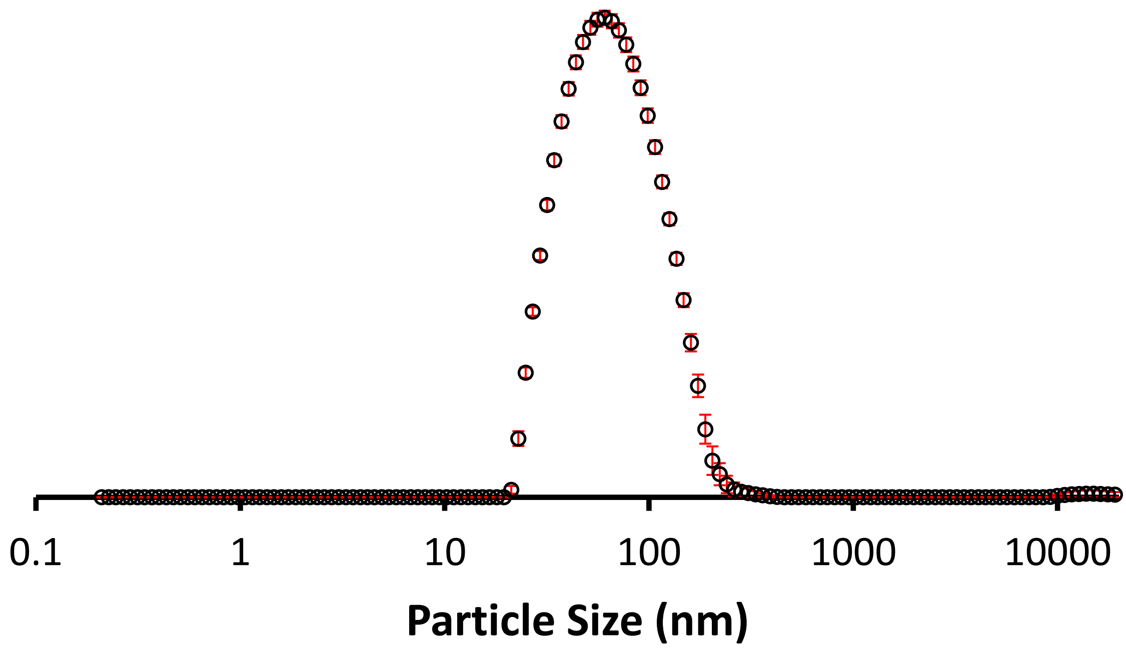

Figure S58 - The average intensity particle size distribution calculated using 9 DLS runs for co-formulation **d** (0.56 mM) in an EtOH: H_2O (1:19) solution at 298 K.

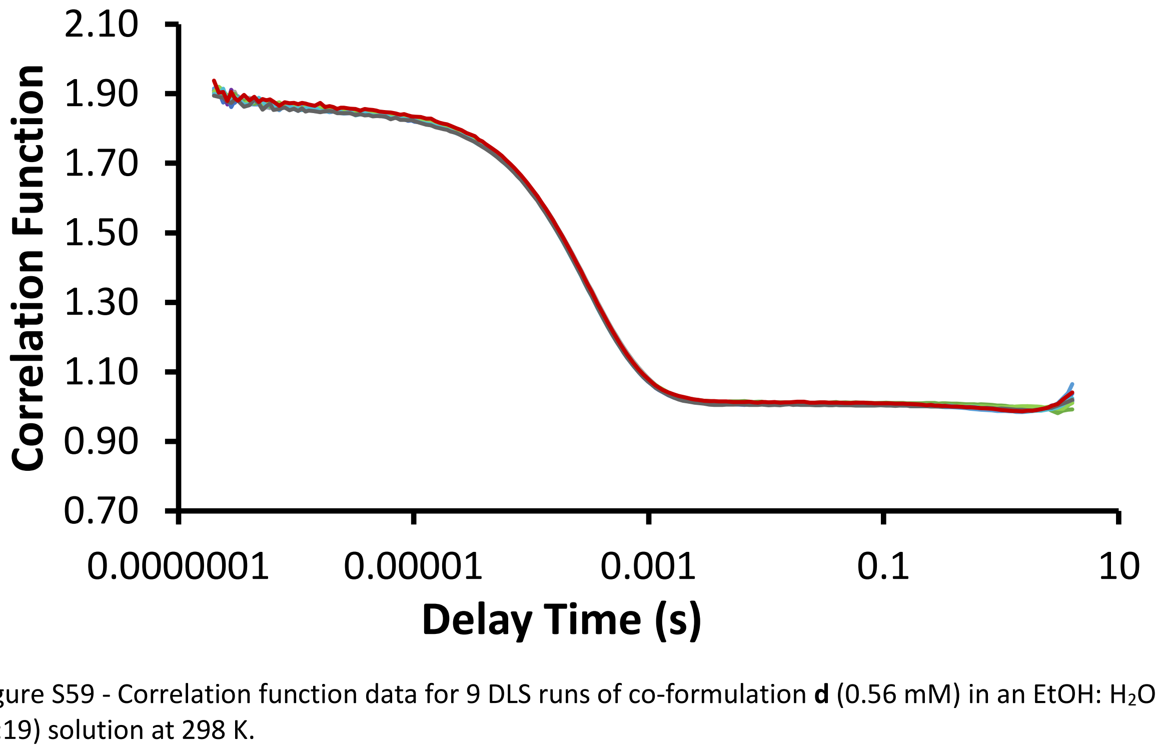

Figure S59 - Correlation function data for 9 DLS runs of co-formulation **d** (0.56 mM) in an EtOH: H_2O (1:19) solution at 298 K.

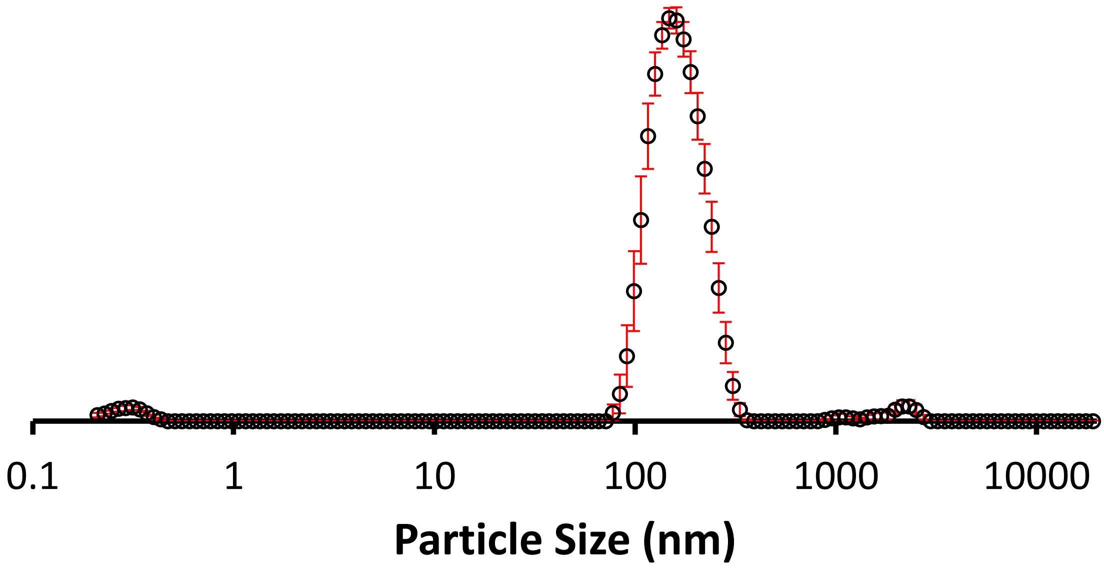

*F*igure S60 - The average intensity particle size distribution calculated using 9 DLS runs for co-formulation **e** (0.56 mM) in an EtOH: H_2O (1:19) solution at 298 K.

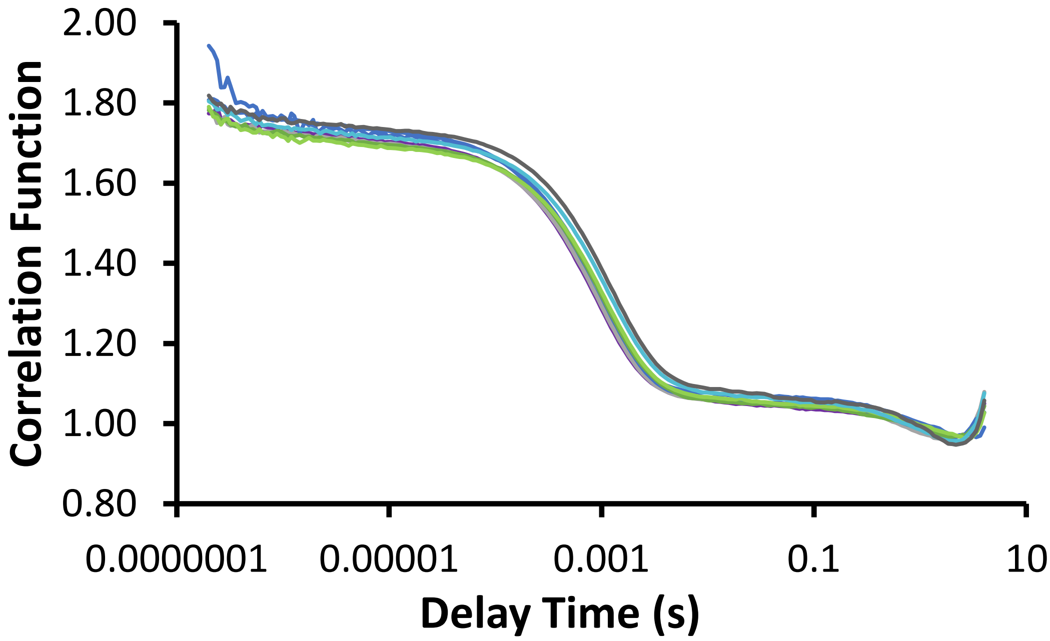

Figure S61 - Correlation function data for 9 DLS runs of co-formulation **e** (0.56 mM) in an EtOH: H_2O (1:19) solution at 298 K.

Zeta potential

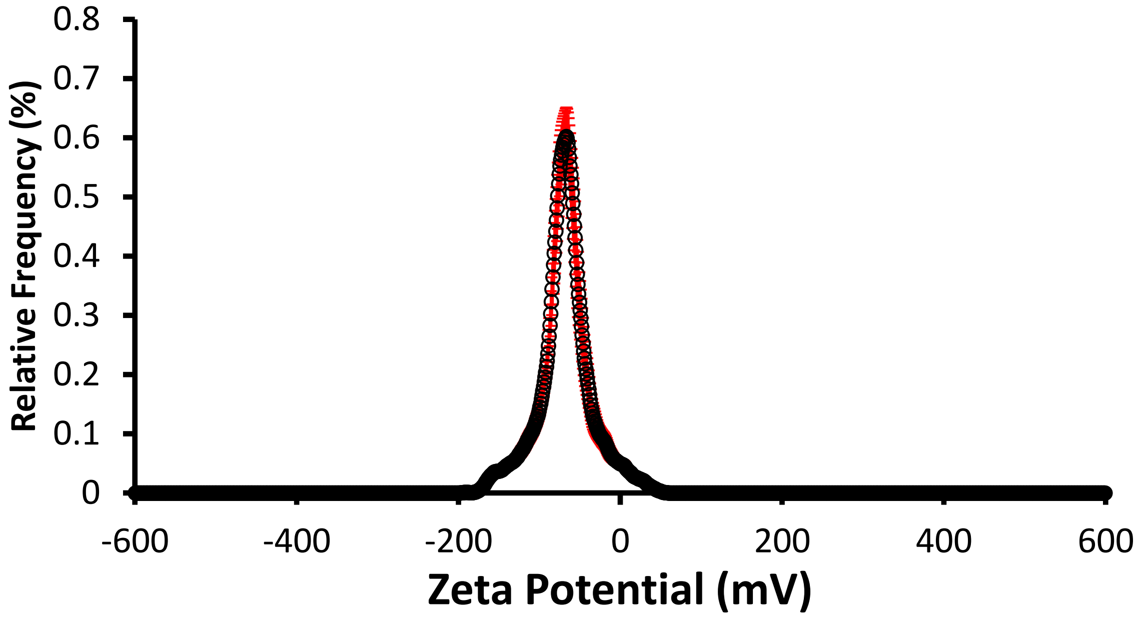

Figure S62 - The average zeta potential distribution calculated using 9 runs for compound **1** (0.56 mM) in an EtOH:H_2O (1:19) solution at 298 K. Average measurement value -66.8 mV.

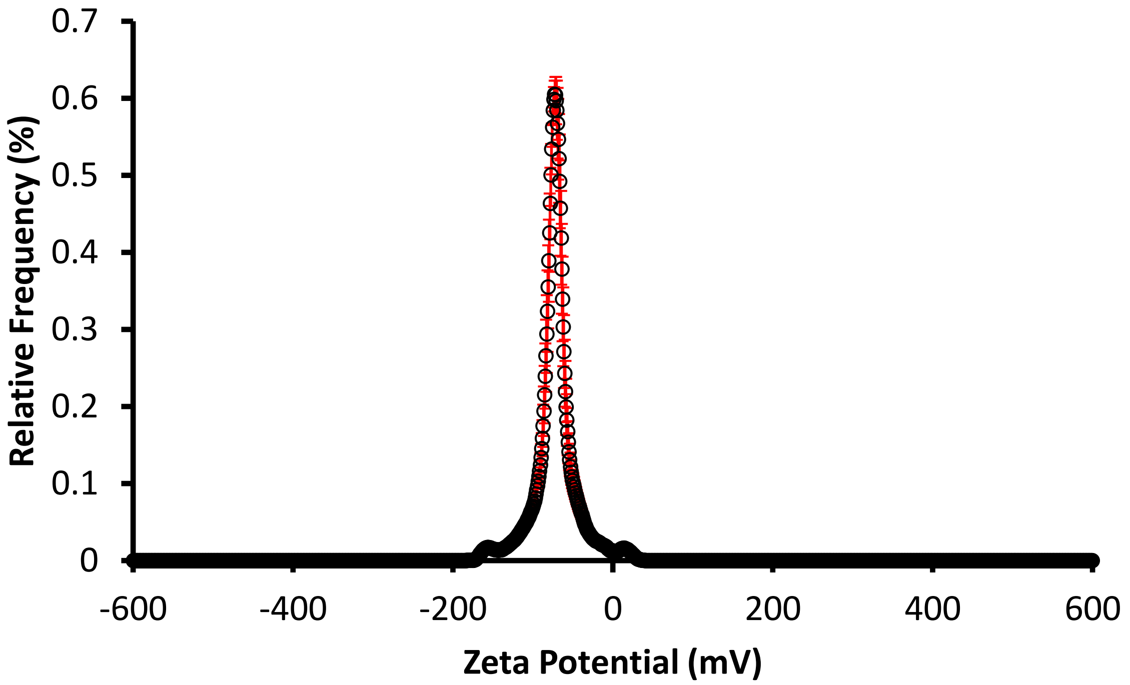

Figure S63 - The average zeta potential distribution calculated using 9 runs for co-formulation **a** (5.56 mM) in an EtOH: H_2O (1:19) solution at 298 K. Average measurement value -71.8 mV.

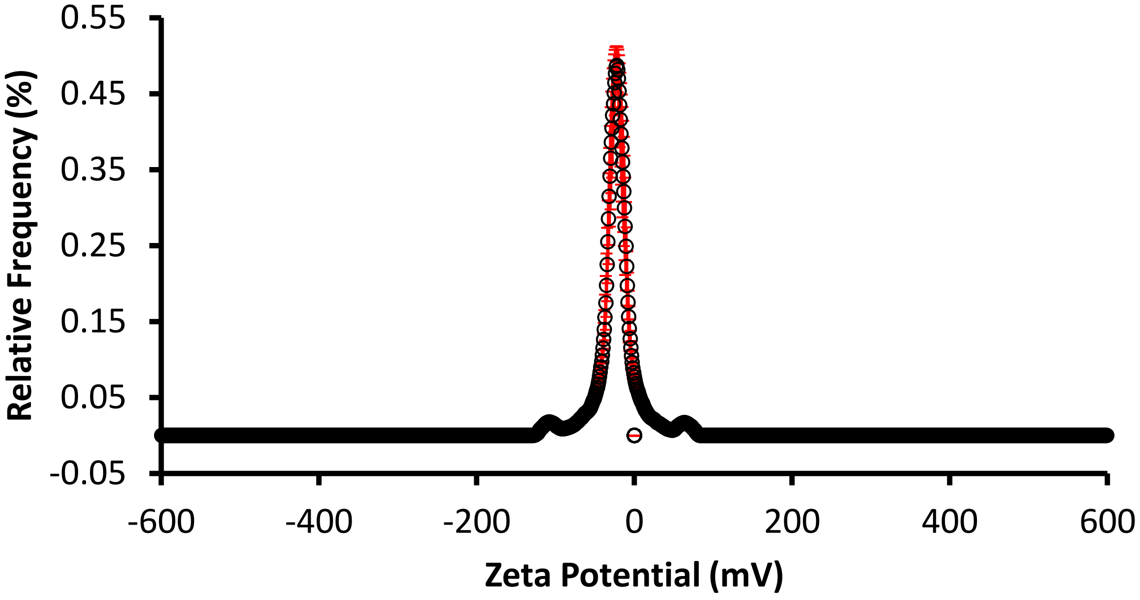

Figure S64 - The average zeta potential distribution calculated using 9 runs for co-formulation **b** (5.56 mM) in an EtOH: H_2O (1:19) solution at 298 K. Average measurement value **-**22.0 mV.

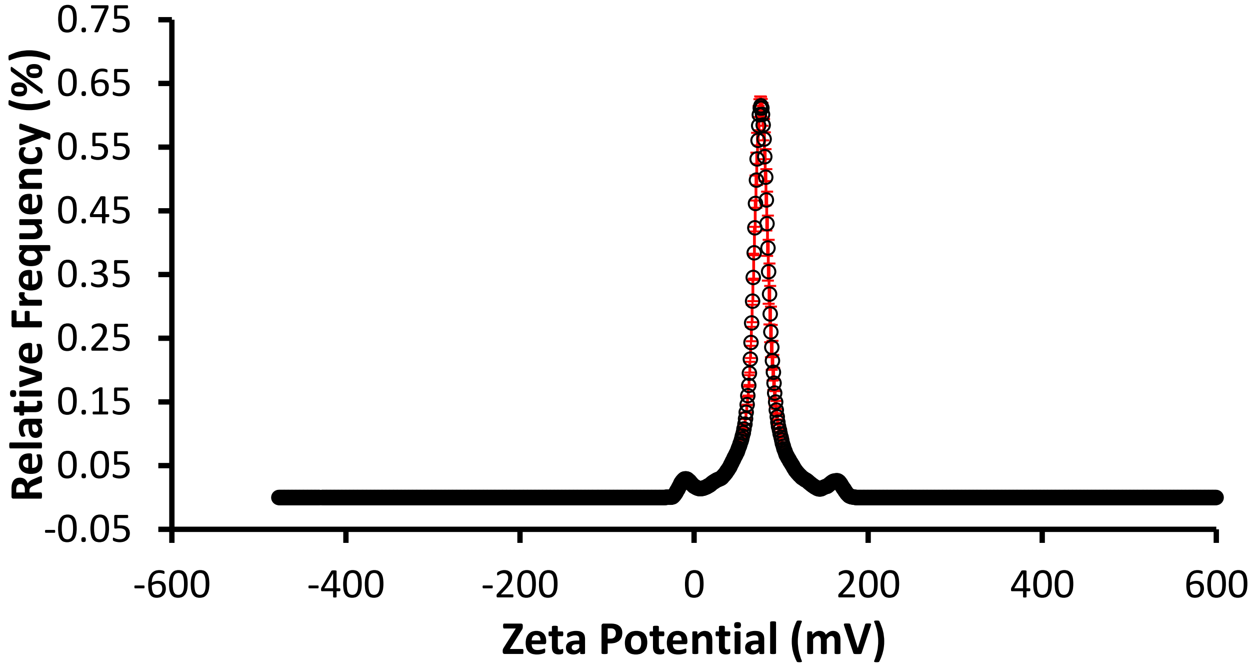

Figure 65 - The average zeta potential distribution calculated using 9 runs for co-formulation **c** (0.56 mM) in an EtOH: H_2O (1:19) solution at 298 K. Average measurement value 76.27 mV.

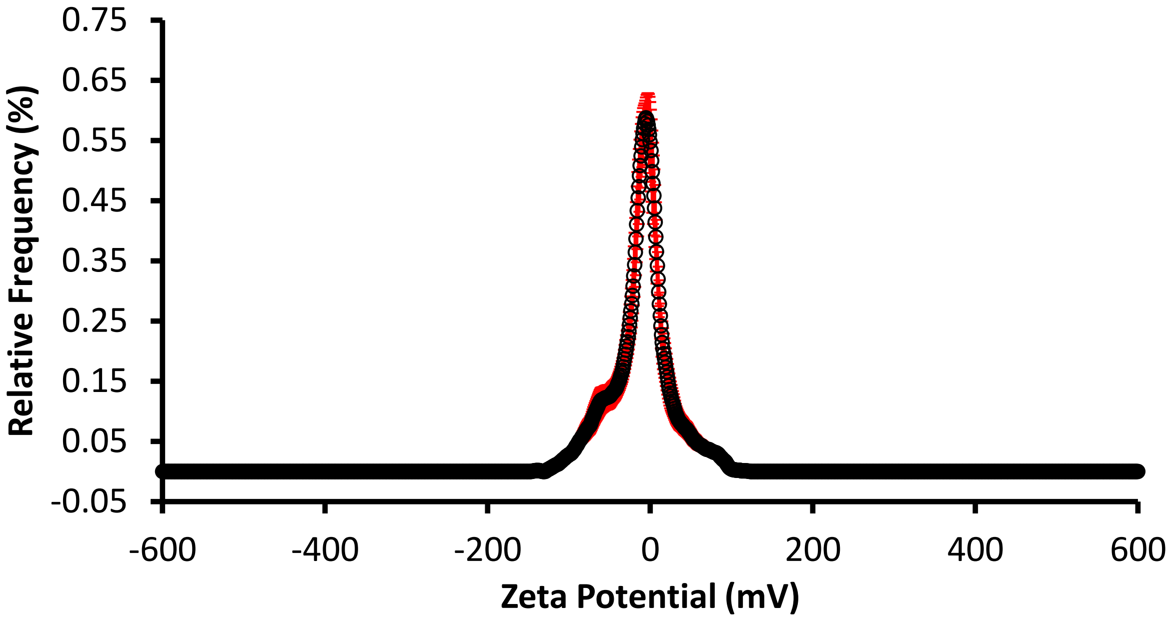

Figure 66 - The average zeta potential distribution calculated using 9 runs for co-formulation **d** (5.56 mM) in an EtOH: H_2O (1:19) solution at 298 K. Average measurement value -2.62 mV.

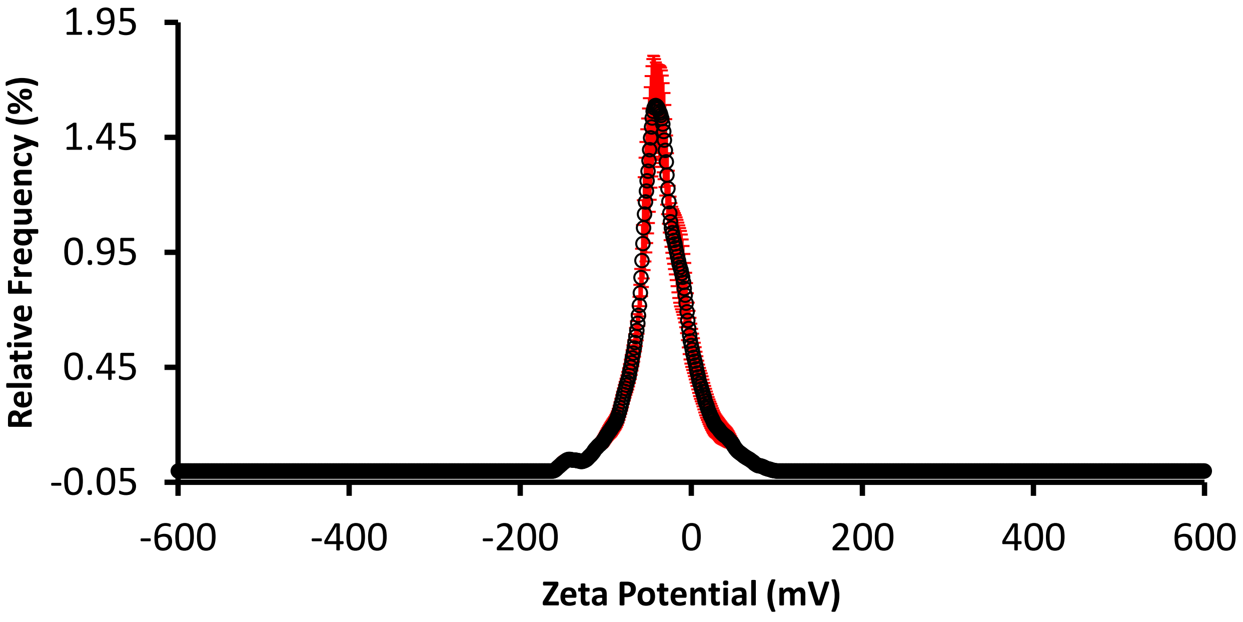

Figure 67 - The average zeta potential distribution calculated using 9 runs for co-formulation **e** (0.56 mM) in an EtOH: H_2O (1:19) solution at 298 K. Average measurement value -42.16 mV.

Surface Tension measurements and Critical micelle concentrations (CMC) determination

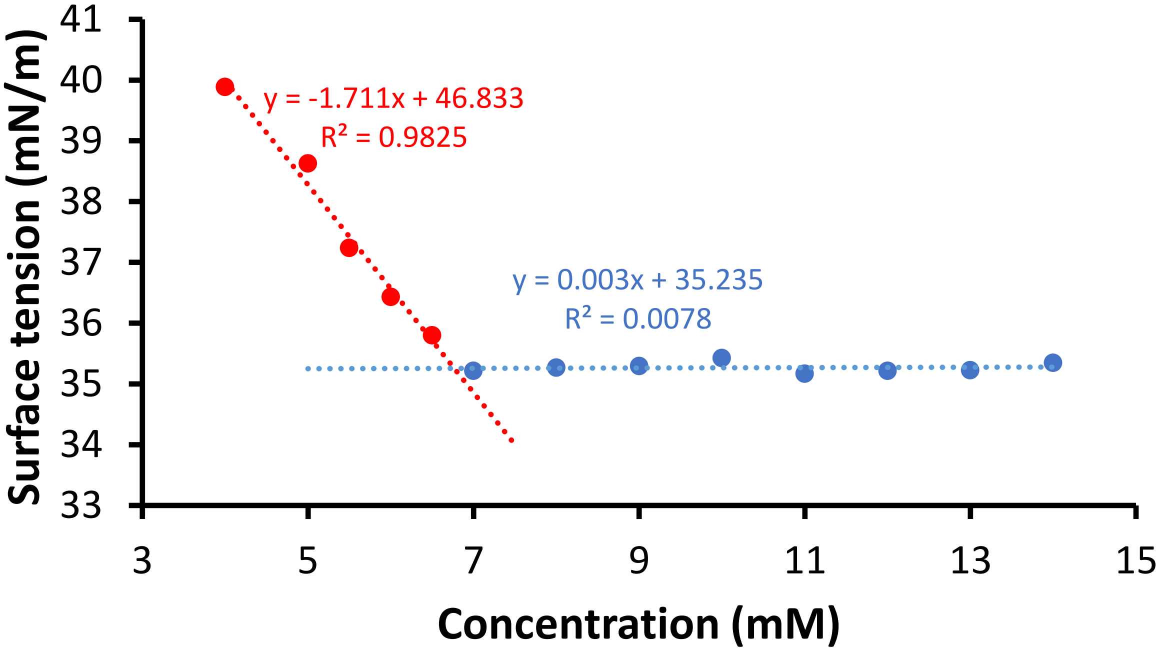

Figure S68 - Calculation of CMC (6.76 mM) for co-formulation **a** in an EtOH:H_2O 1:19 mixture using surface tension measurements.

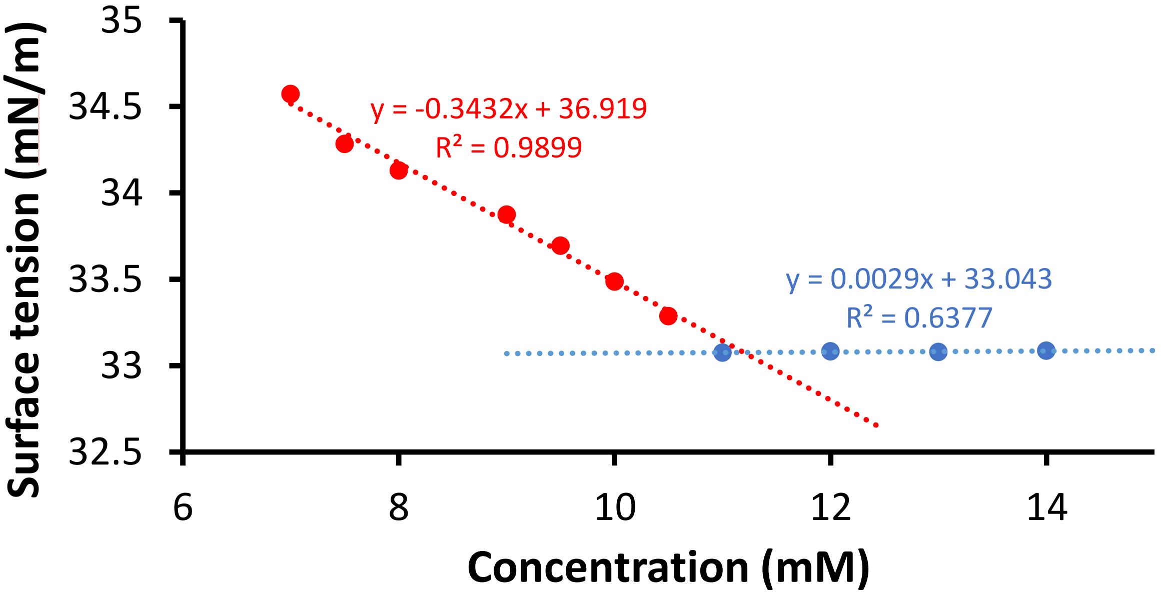

Figure S69 - Calculation of CMC (11.21 mM) for co-formulation **b** in an EtOH:H_2O 1:19 mixture using surface tension measurements.

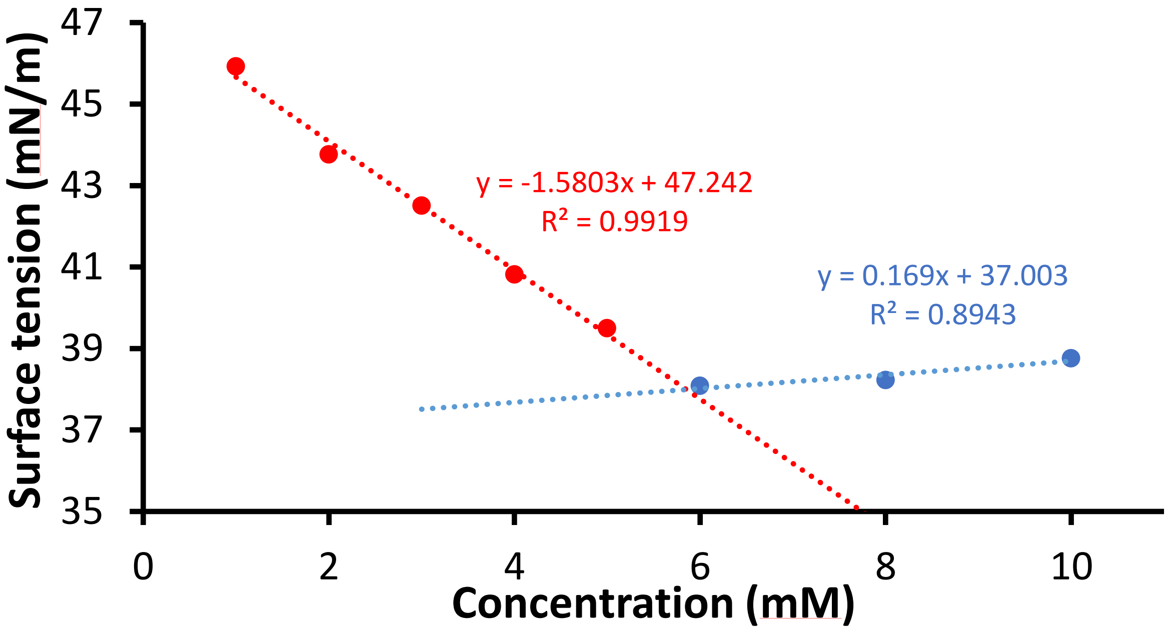

Figure S70 - Calculation of CMC (5.58 mM) for co-formulation **c** in an EtOH:H_2O 1:19 mixture using surface tension measurements.

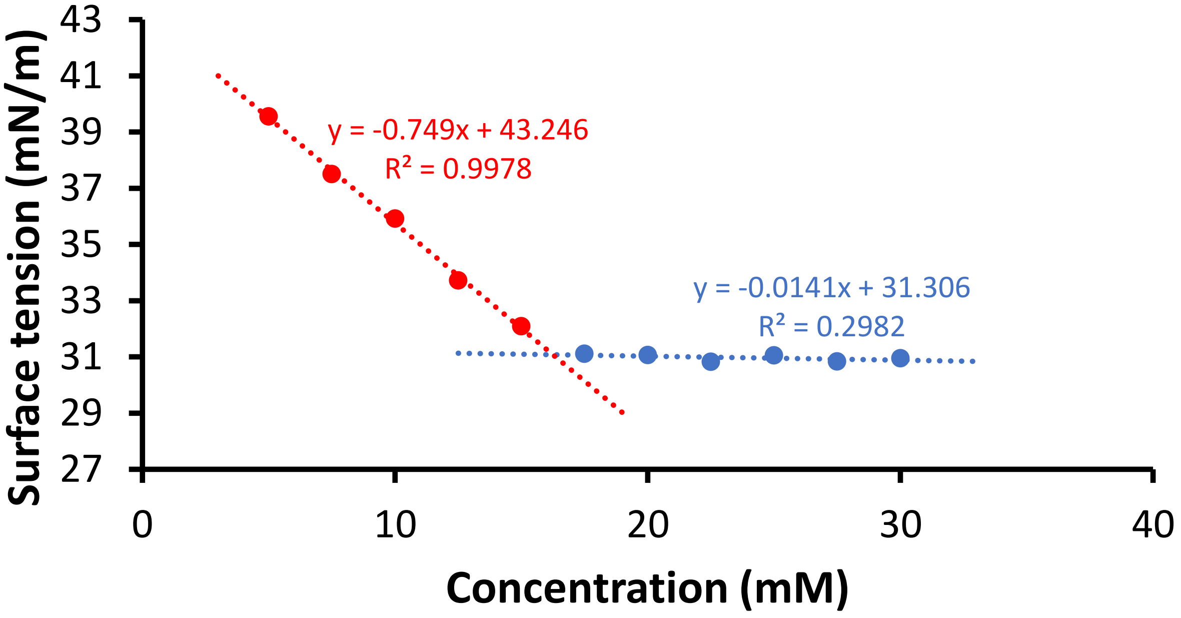

Figure S71 - Calculation of CMC (16.24 mM) for co-formulation **d** in an EtOH:H_2O 1:19 mixture using surface tension measurements.

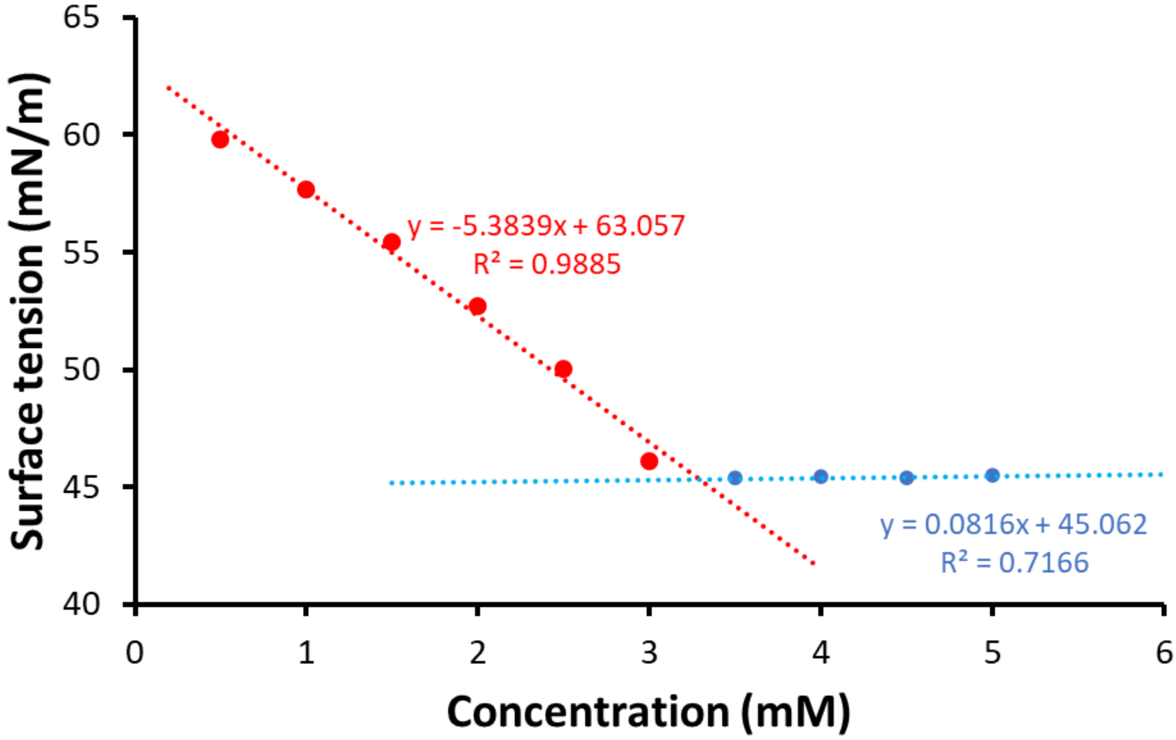

Figure S72 - Calculation of CMC (3.29 mM) for co-formulation **e** in an EtOH:H_2O 1:19 mixture using surface tension measurements.

Overview

Table 5 - Overview of average DLS intensity particle size distribution peak maxima, zeta potential and CMC, measurements obtained for a H_2O/ 5.0 % EtOH solution of an SSA (**1**) or co-formulation **a-e** (5.56 mM) at 298 K.* = experiments were performed at 0.56 mM due to solubility issues.

Co-formulation	Peak maxima (nm)	Polydispersity (%)	Zeta potential (mV)	CMC (mM)
**1** only (5.56 mM)[5]	164	24 (± 1.20)	-76	10.39
**1** only (0.56 mM)*[5,6]	142	25 (± 0.68)	-66	n/a
a	200	20 (± 1.56)	-72	6.76
b	209	17 (± 1.65)	-22	11.21
c*	240	25 (± 0.75)	+76	5.58
d	136	13 (± 1.26)	-3	16.24
e*	160	21 (± 21.51)	-42	3.29

Single Crystal X-ray structures

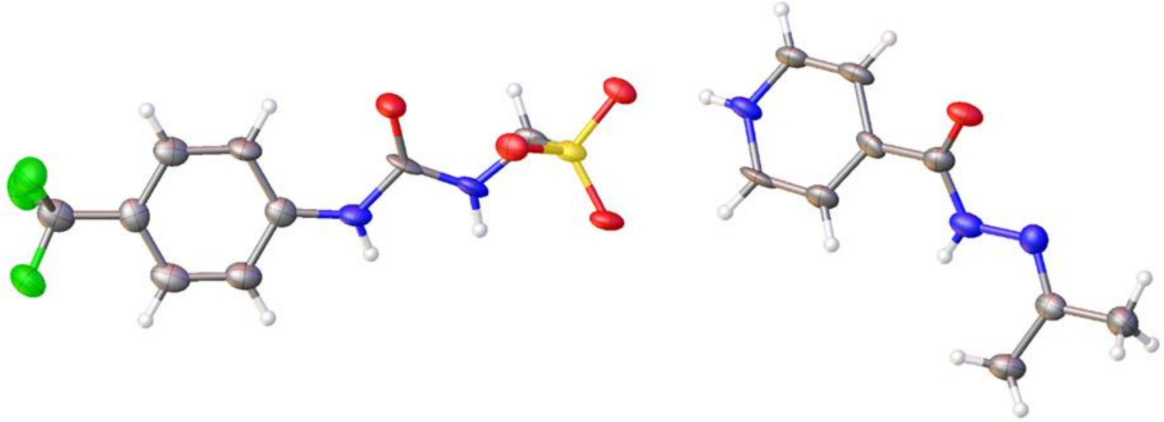

Figure S73 - A single crystal X-ray structure obtained from a single crystal sample produced from a solution of co-formulation *a* in acetone. Red = oxygen; yellow = sulfur; green = fluorine; blue = nitrogen; white = hydrogen; grey = carbon. CCDC 1999018, $C_{18}H_{20}F_3N_5O_5S$ (M = 475.45): orthorhombic, space group P n a 21, a = 8.3209(5) Å, b = 46.084(5) Å, c = 5.1719(2) Å, α = 90°, β = 90°, γ = 90°, V = 1983.2(2) Å^3, Z = 4, T = 100(1) K, CuK\α = 1.5418 Å, Dcalc = 1.592 g/cm^3, 12545 reflections measured (7.674 ≤ 2Θ ≤ 146.356), 3395 unique (R_{int} = 0.1296, R_{sigma} = 0.1161) which were used in all calculations. The final R_1 was 0.0787 (I > 2σ(I)) and wR_2 was 0.1732 (all data).

Table S6 - Hydrogen bond distances and angles observed for a single crystal sample produced from a solution of **co-formulation a**, calculated from the single crystal X-ray structure shown in Figure S73.

Hydrogen bond donor	Hydrogen bond acceptor	Hydrogen bond length (D•••A) (Å)	Hydrogen bond angle (D-H•••A) (°)
N1	O2	3.172 (10)	151.9 (6)
N2	O2	3.042 (10)	158.0 (5)
N2	O3	3.016 (10)	136.8 (5)
N3	O3	2.657 (10)	169.0 (5)
N4	O5	3.173 (11)	153.6 (5)

Co-formulation growth curves

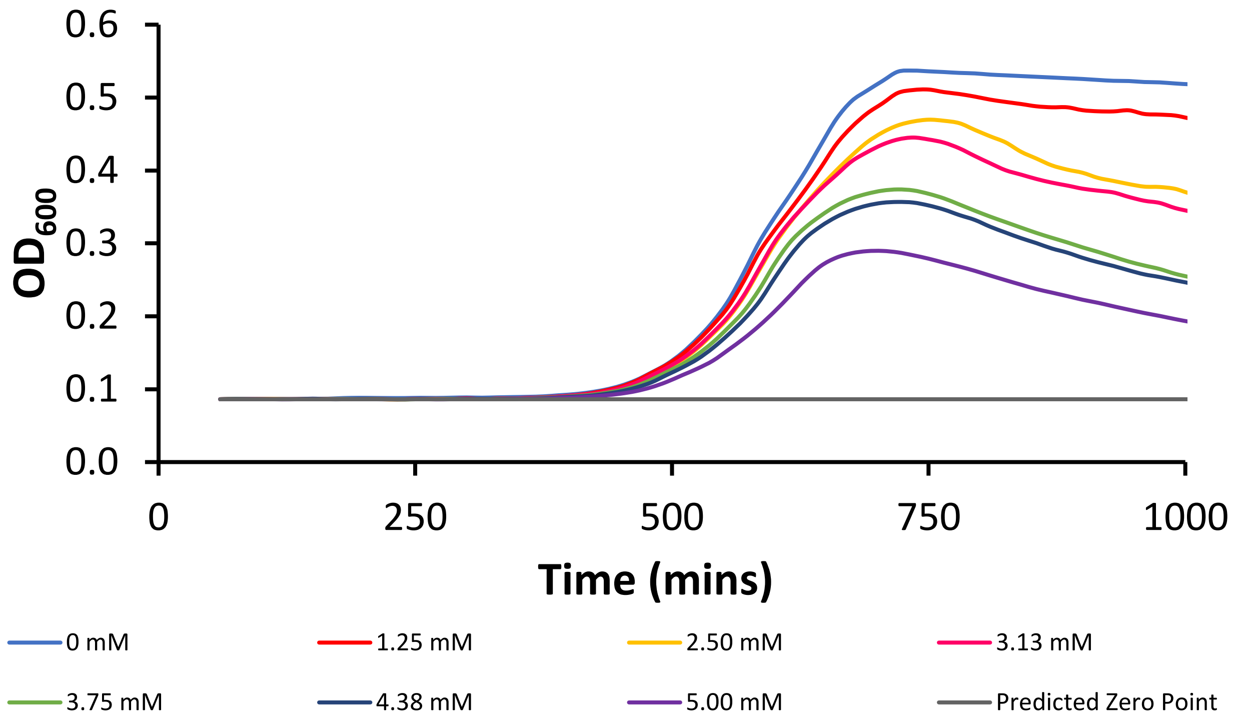

Figure S74 - Averaged growth curves created from absorbance readings of *E.coli* DH10B in the presence of compound **1** at varying concentrations.

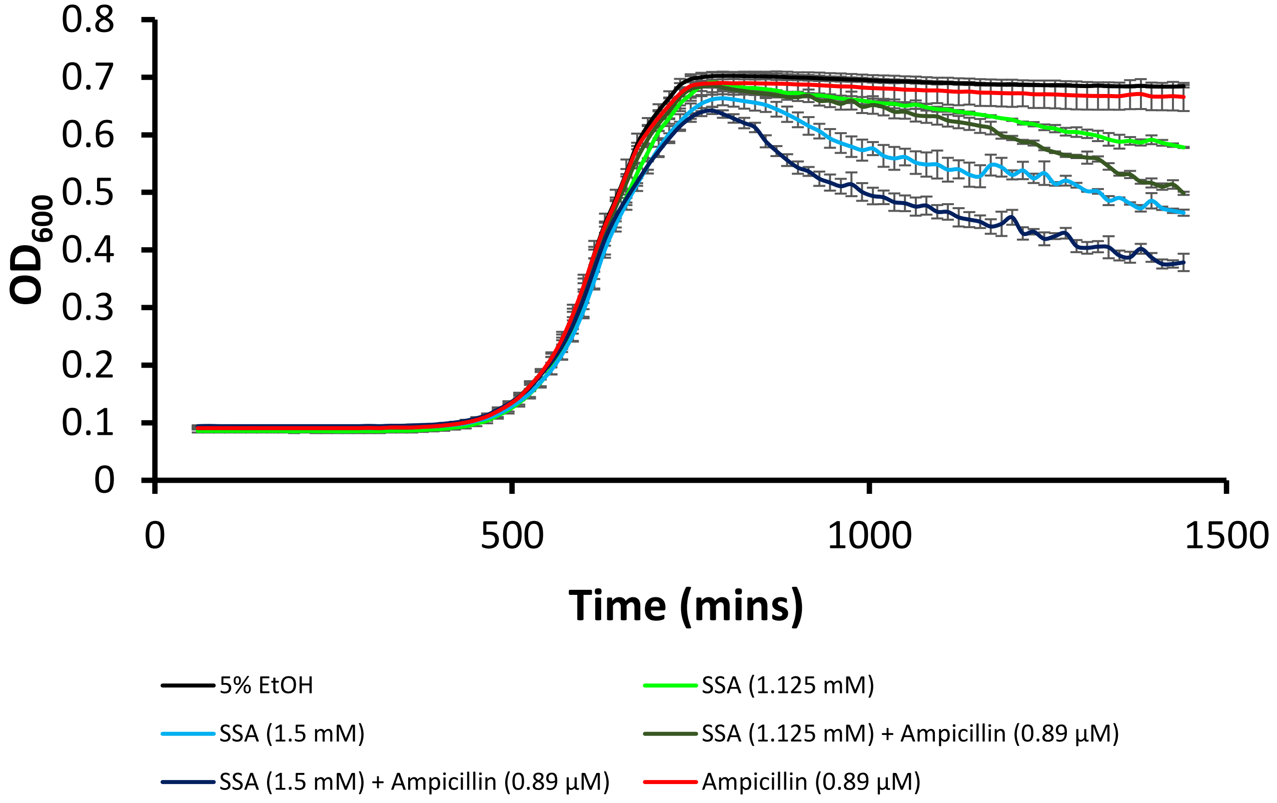

Figure S75 - Averaged growth curves created from absorbance readings of *E. coli* DH10B in the presence of SSA **1**, ampicillin (0.89 µM) and co-formulated systems following a 10 min incubation of SSA prior to the addition of the ampicillin.

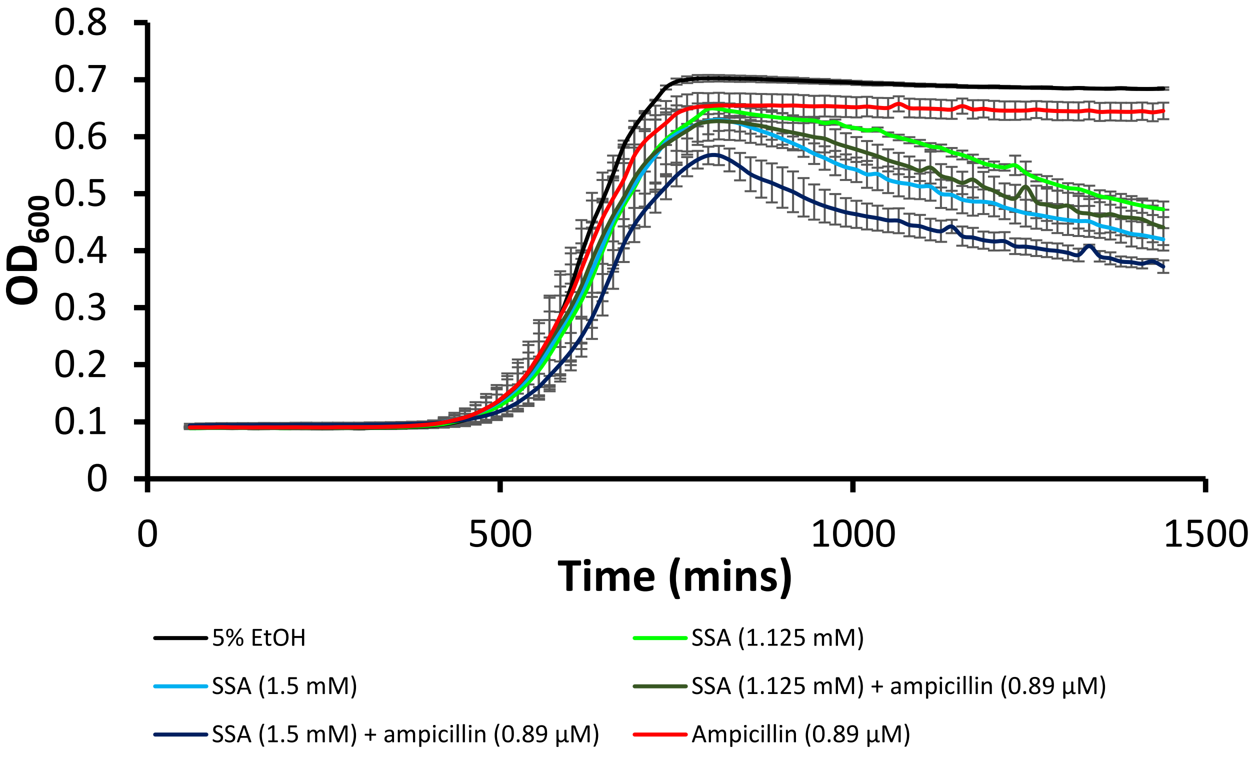

Figure S76 - Averaged growth curves created from absorbance readings of *E. coli* DH10B in the presence of SSA **1**, ampicillin (0.89 µM) and co-formulated systems following a 10 min incubation of ampicillin prior to the addition of the SSA.

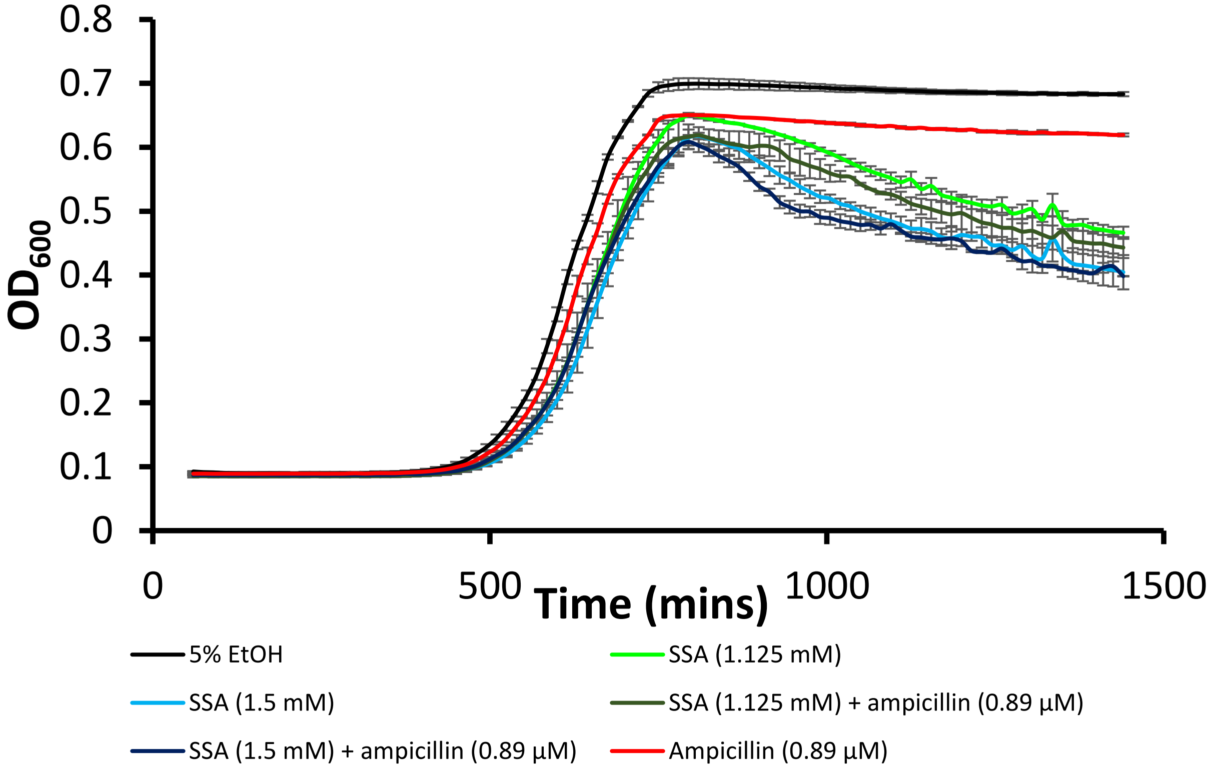

Figure S77 - Averaged growth curves created from absorbance readings of *E. coli* DH10B in the presence of SSA **1**, ampicillin (0.89 µM) and co-formulated systems with no prior incubation.

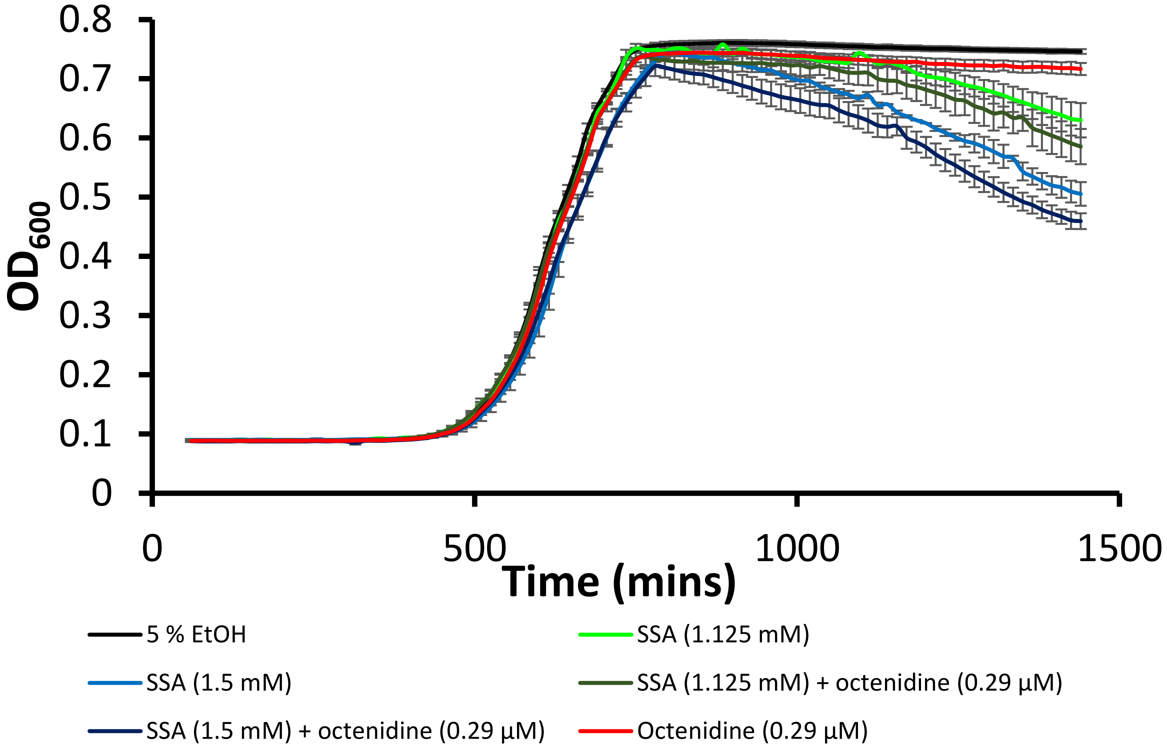

Figure S78 - Averaged growth curves created from absorbance readings of *E. coli* DH10B in the presence of SSA **1**, octenidine (0.29 µM) and co-formulated systems following a 10 min incubation of SSA prior to the addition of the octenidine.

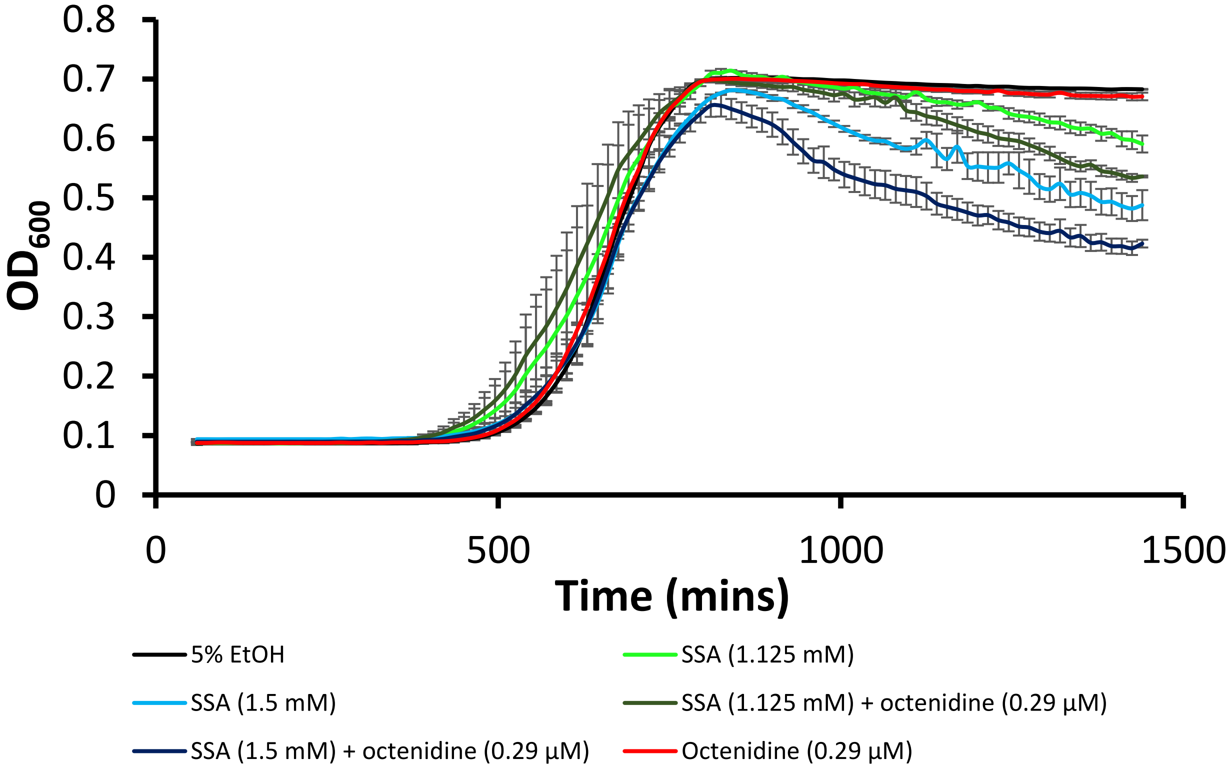

Figure S79 - Averaged growth curves created from absorbance readings of *E. coli* DH10B in the presence of SSA **1**, octenidine (0.29 µM) and co-formulated systems following a 10 min incubation of octenidine prior to the addition of the SSA.

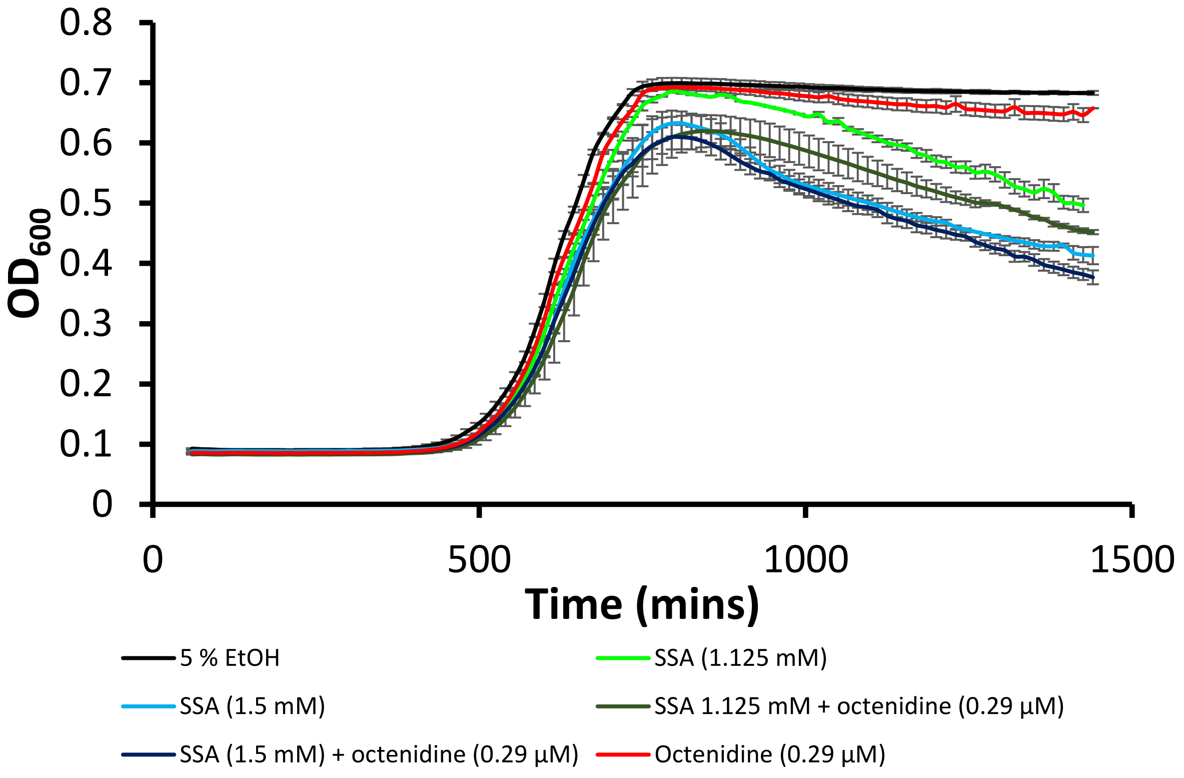

Figure S80 -Averaged growth curves created from absorbance readings of *E. coli* DH10B in the presence of SSA **1**, octenidine (0.29 µM) and co-formulated systems with no prior incubation.

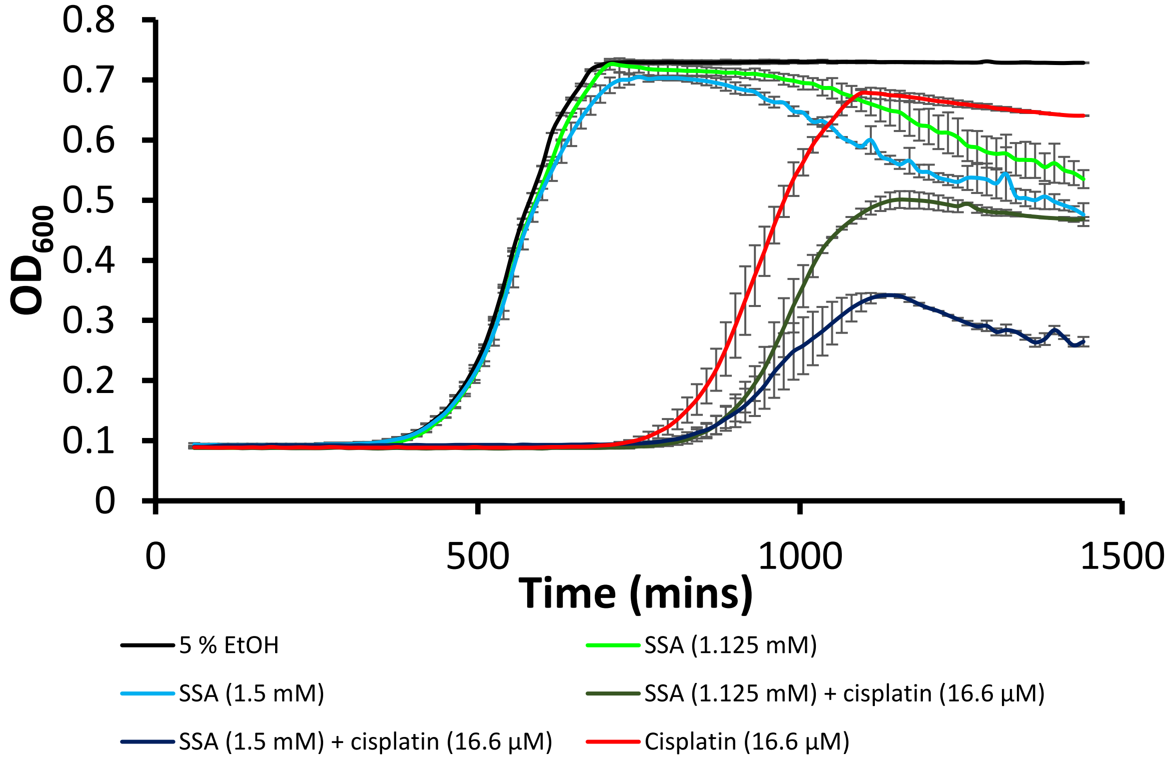

Figure S81 -Averaged growth curves created from absorbance readings of *E. coli* DH10B in the presence of SSA **1**, cisplatin (16.6 µM) and co-formulated systems following a 10 min incubation of SSA prior to the addition of the cisplatin.

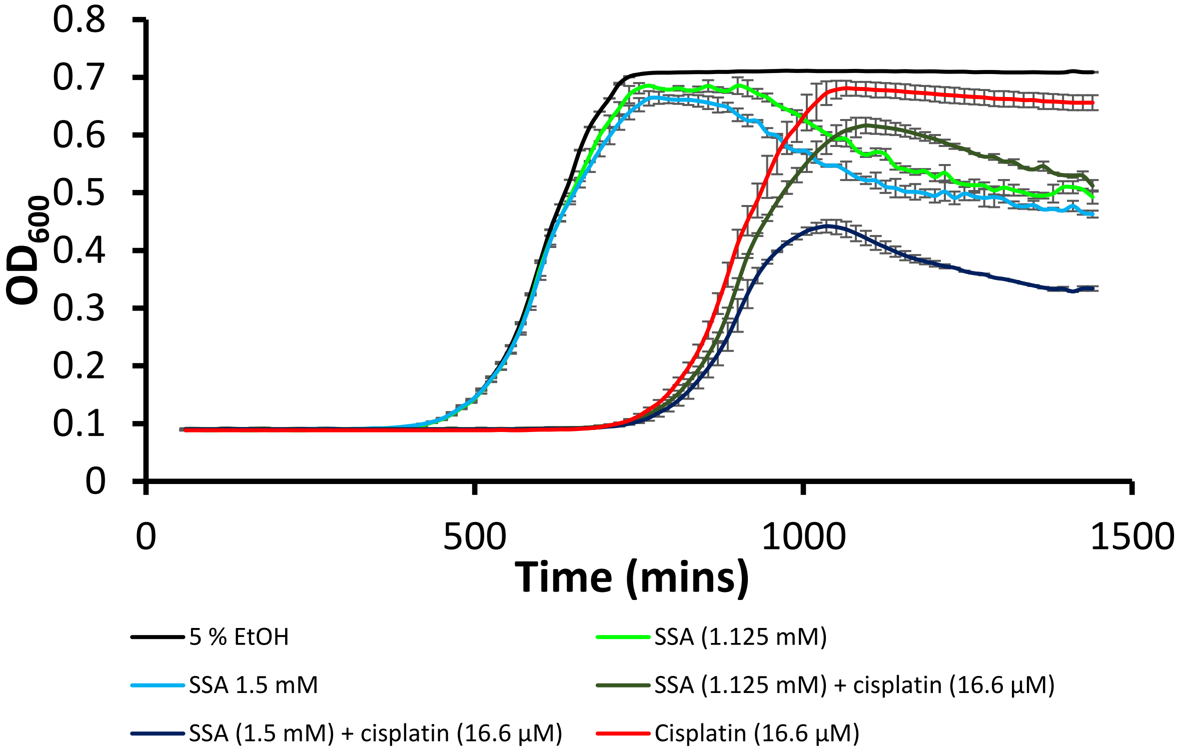

Figure S82 - Averaged growth curves created from absorbance readings of *E. coli* DH10B in the presence of SSA **1**, cisplatin (16.6 μM) and co-formulated systems following a 10 min incubation of cisplatin prior to the addition of the SSA.

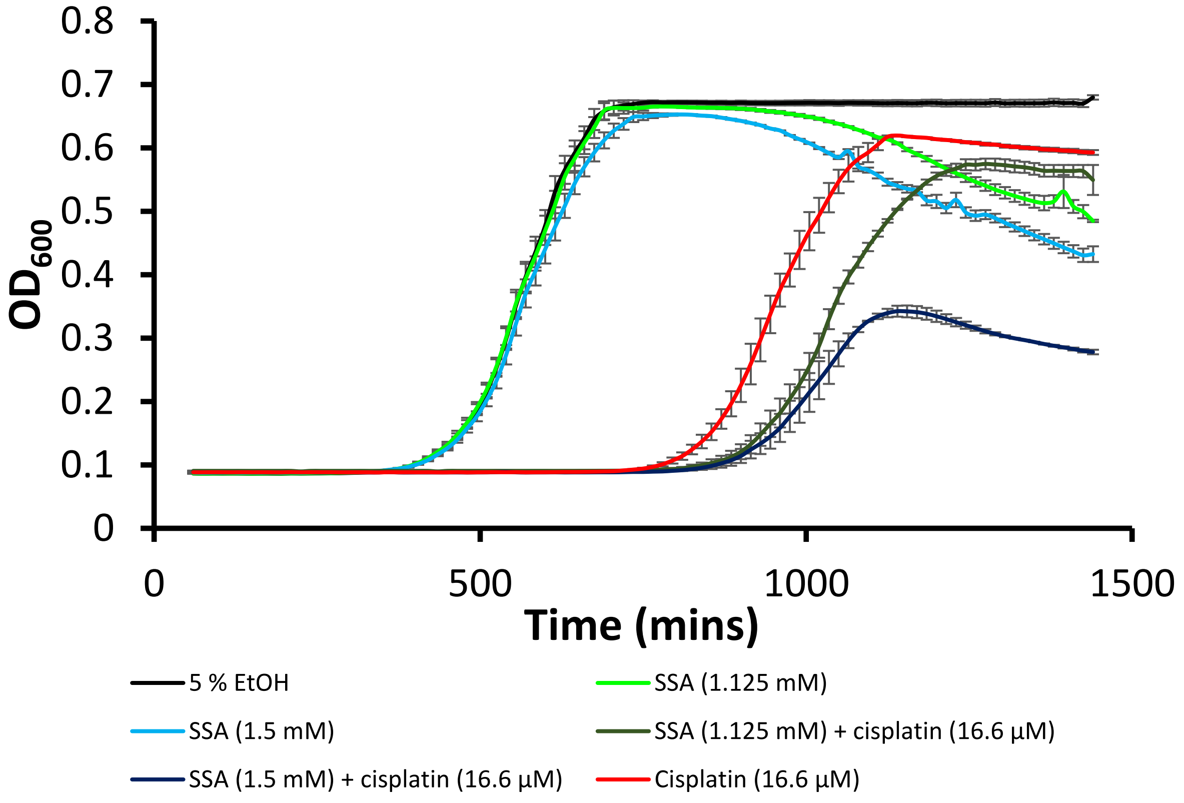

Figure S83 - Averaged growth curves created from absorbance readings of *E. coli* DH10B in the presence of SSA **1**, cisplatin (16.6 μM) and co-formulated systems with no prior incubation.

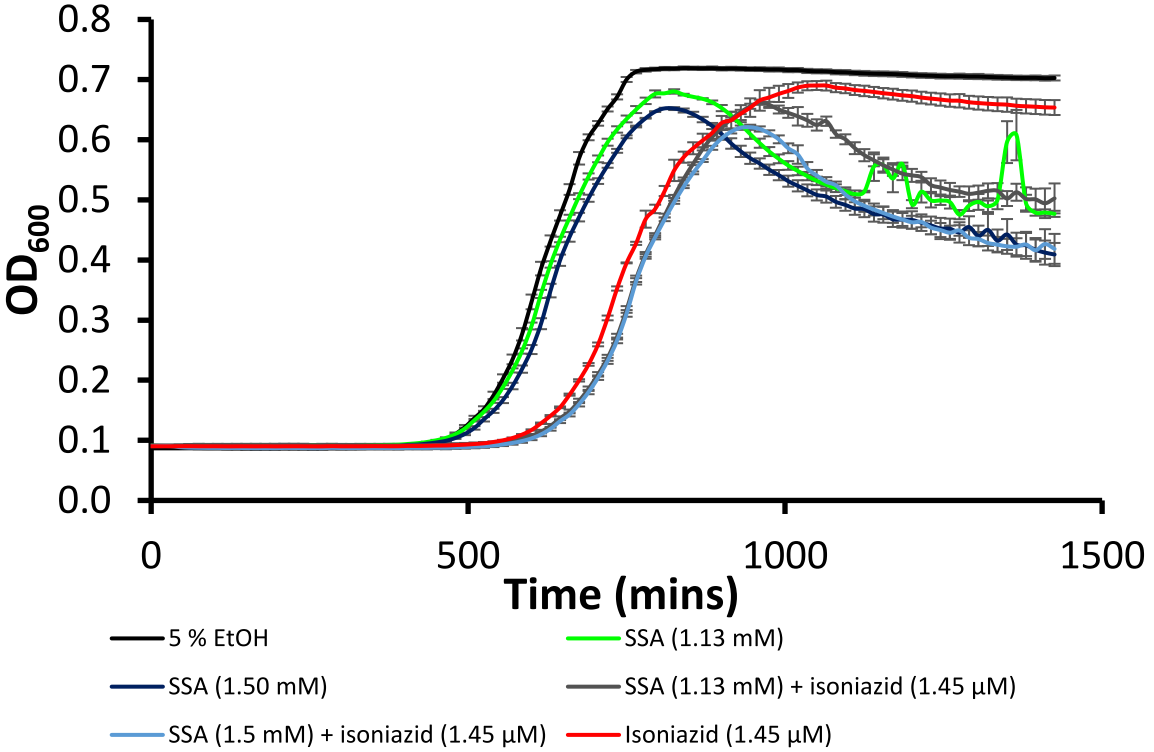

Figure S84 - Averaged growth curves created from absorbance readings of *E. coli* DH10B in the presence of SSA **1**, isoniazid (1.45 µM) and co-formulated systems with no prior incubation.

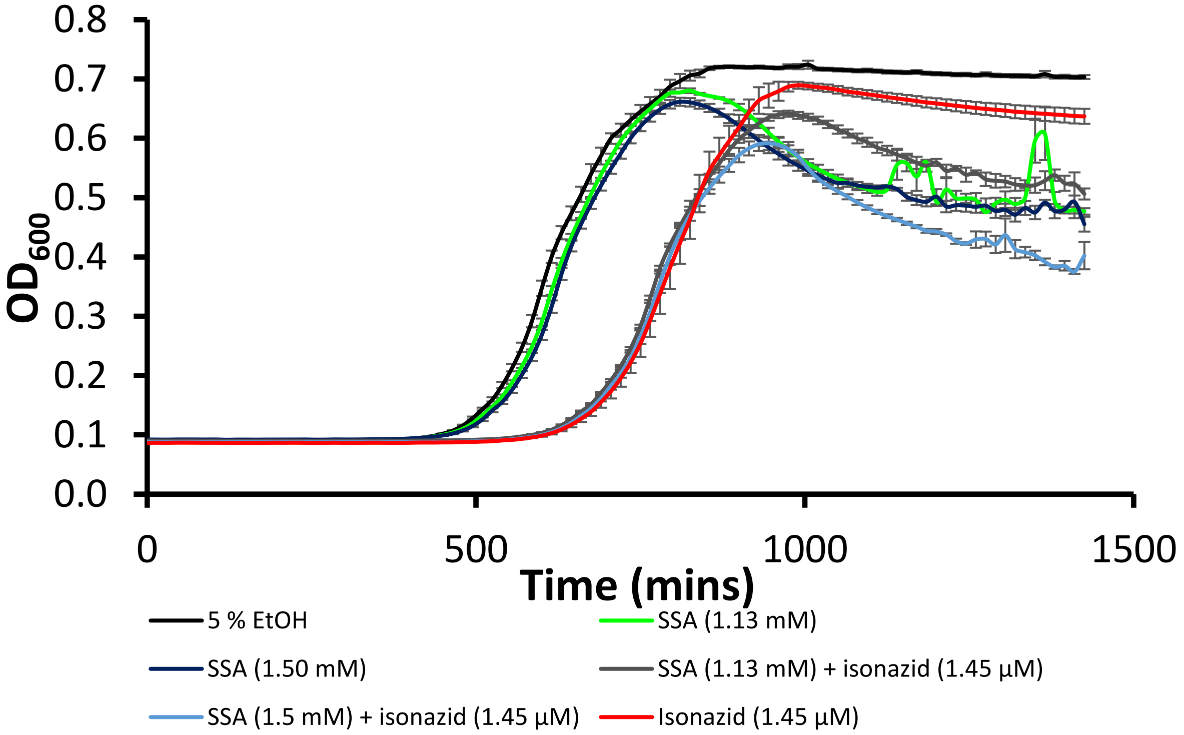

Figure S85 - Averaged growth curves created from absorbance readings of *E. coli* DH10B in the presence of SSA **1**, isoniazid (1.45 µM) and co-formulated systems following a 10 min incubation of isoniazid prior to the addition of the SSA.

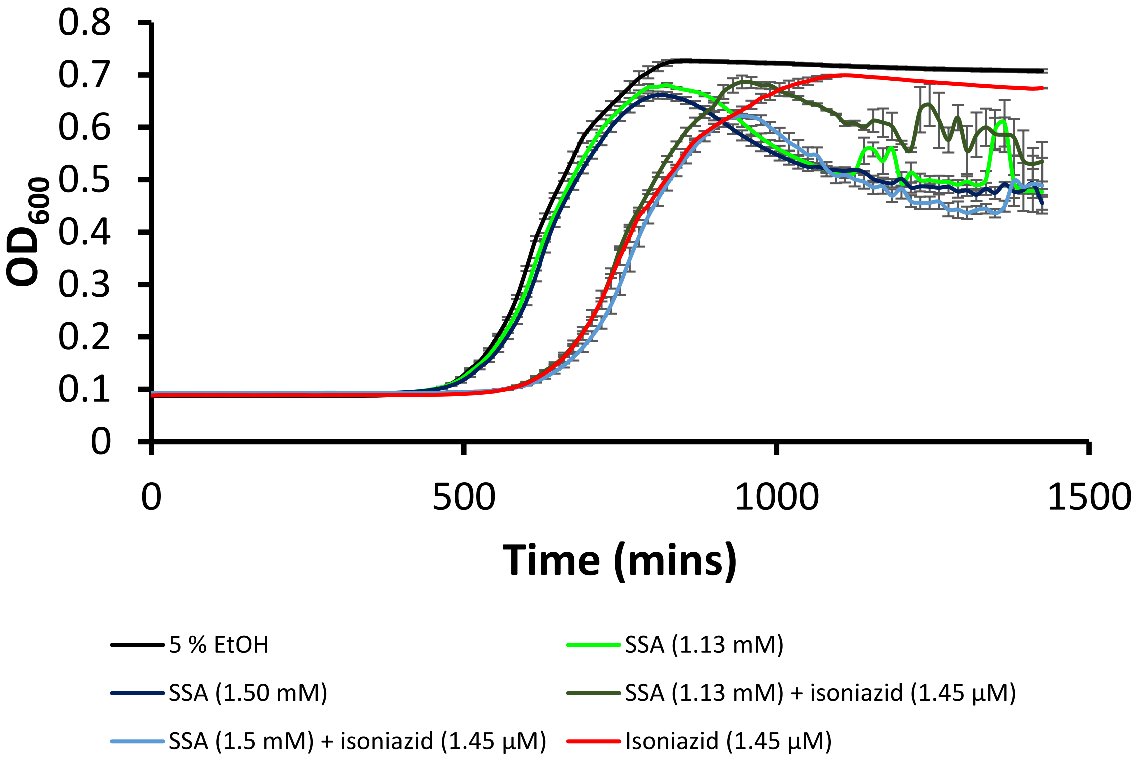

Figure S86 - Averaged growth curves created from absorbance readings of *E. coli* DH10B in the presence of SSA **1**, isoniazid (1.45 µM) and co-formulated systems following a 10 min incubation of SSA prior to the addition of the isoniazid.

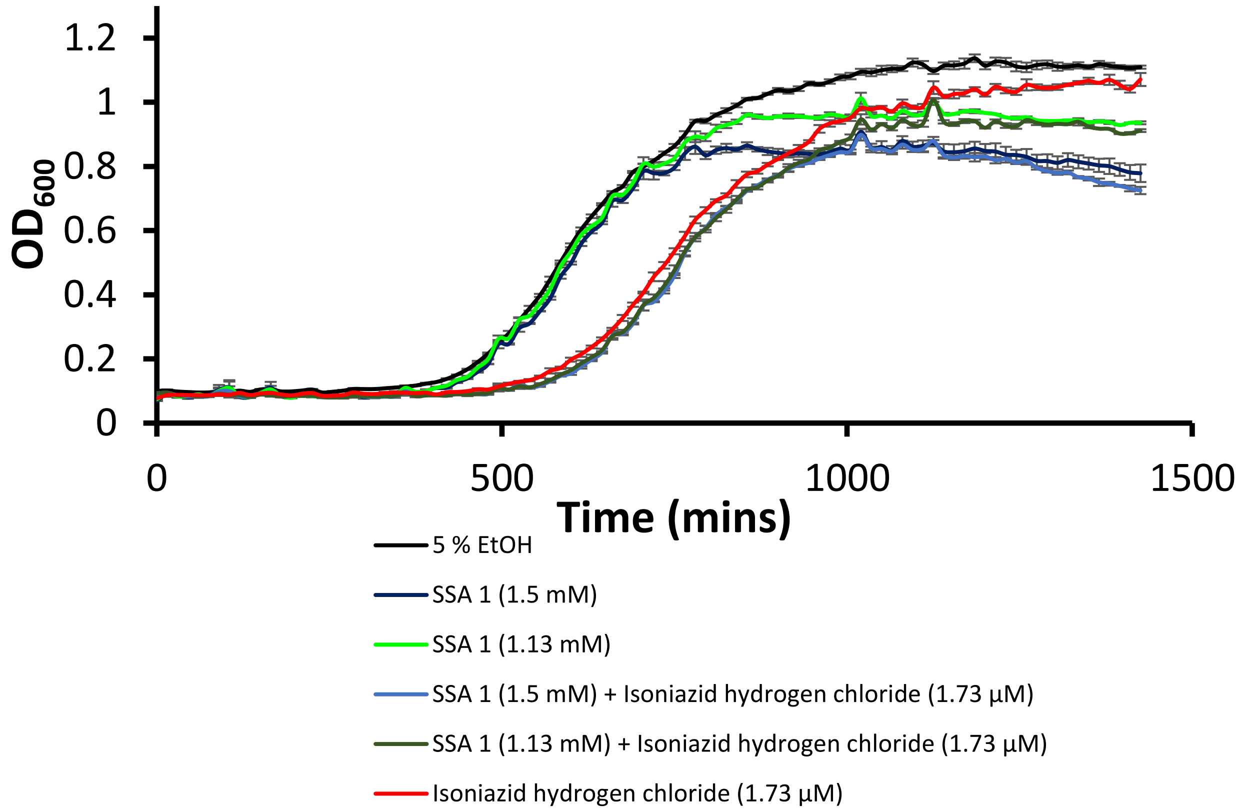

Figure S87 - Averaged growth curves created from absorbance readings of *E. coli* DH10B in the presence of SSA **1**, isoniazid hydrogen chloride (1.73 µM) and co-formulated systems with no prior incubation.

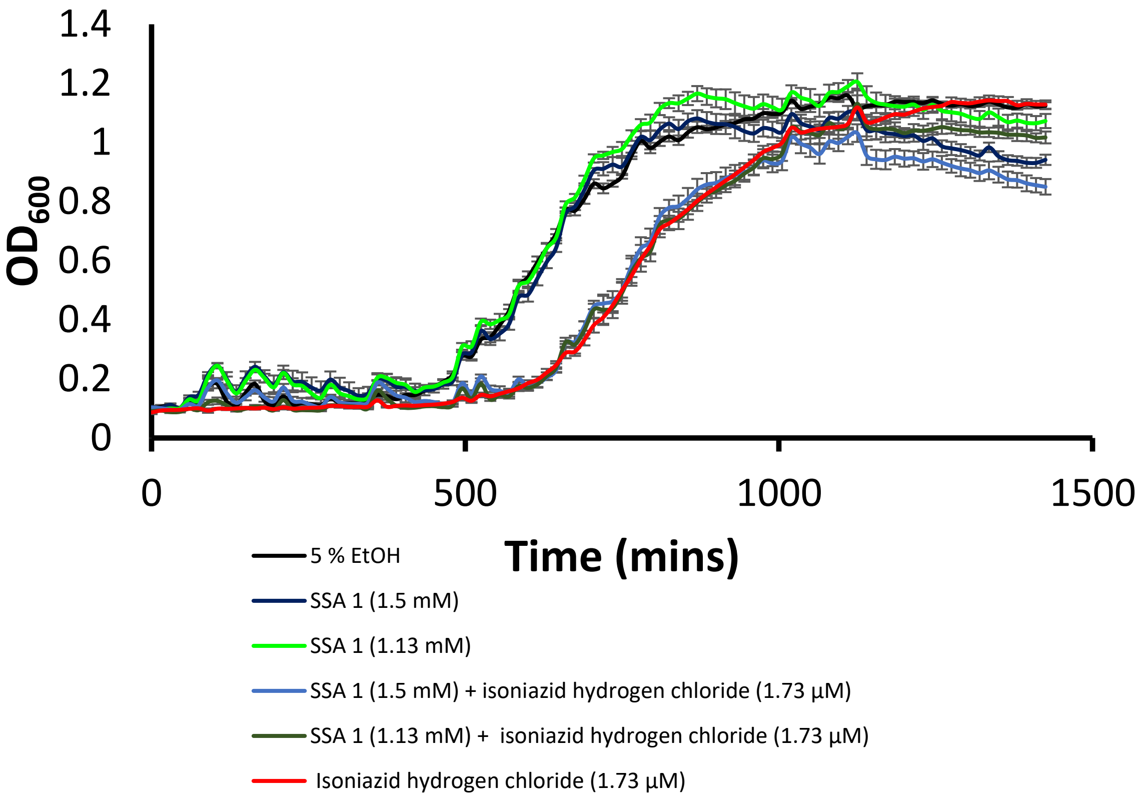

Figure S88 - Averaged growth curves created from absorbance readings of *E. coli* DH10B in the presence of SSA **1**, isoniazid hydrogen chloride (1.73 µM) and co-formulated systems following a 10 min incubation of isoniazid hydrogen chloride prior to the addition of the SSA.

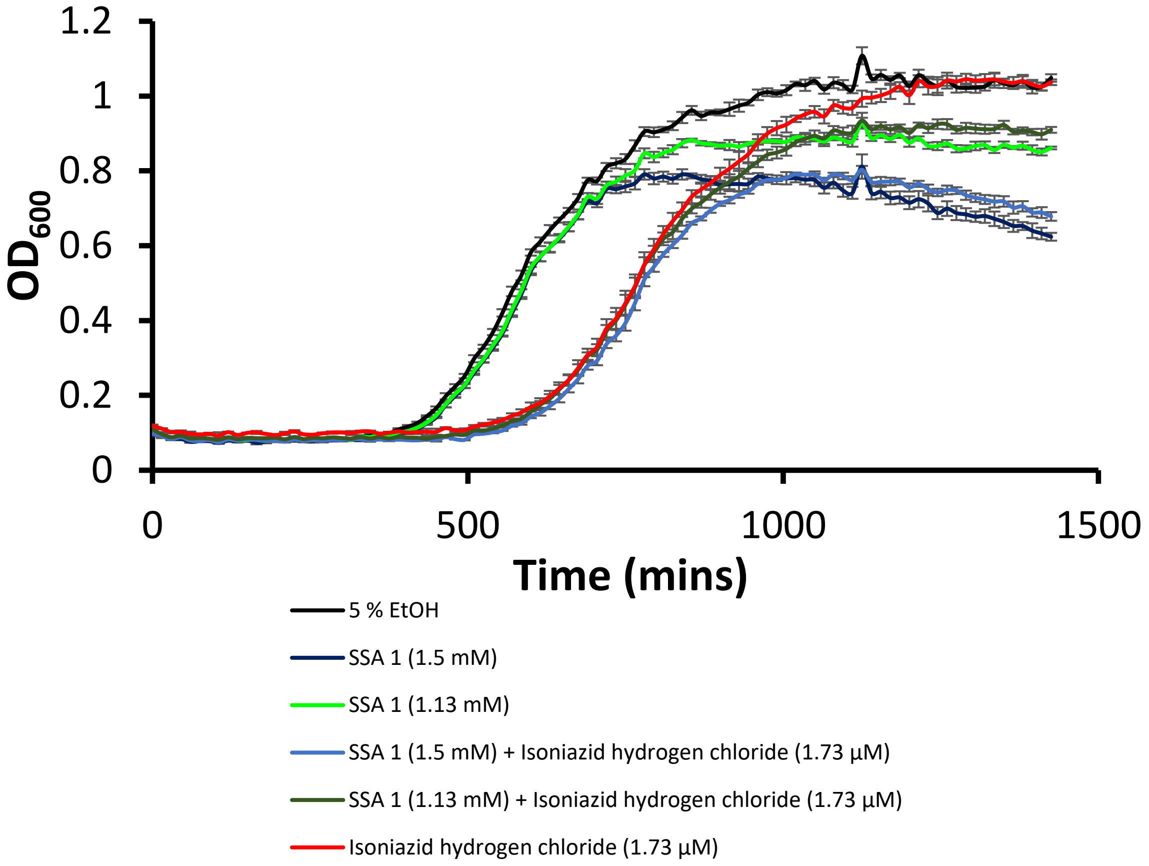

Figure S89 - Averaged growth curves created from absorbance readings of *E. coli* DH10B in the presence of SSA **1**, isoniazid hydrogen chloride (1.73 µM) and co-formulated systems following a 10 min incubation of SSA prior to the addition of the isoniazid hydrogen chloride.

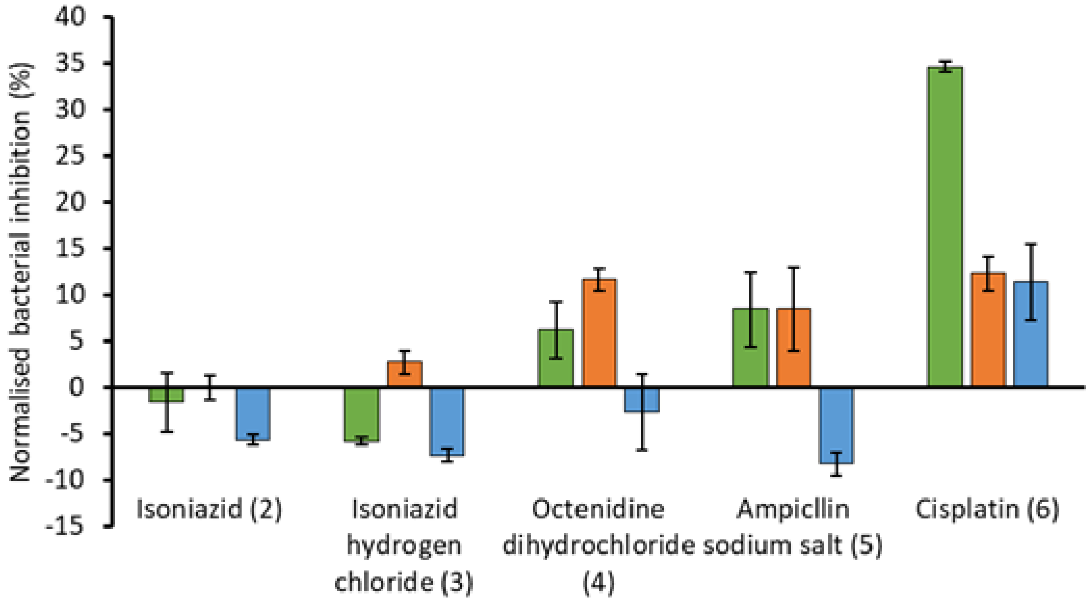

Figure S90 – Bar chart showing the increase/decrease in efficacy of the antimicrobial/therapeutic agent (**2**-**6**) when supplied as a co-therapy in the presence of SSA **1**. These results have been normalised against the combined bacterial growth inhibition effects of **1** and co-formulants **2**-**6** alone. OD_{600} measurements were obtained at 1100 minutes against *E. coli* DH10B in the presence of SSA **1** (1.5 mM) and either isoniazid (**2**) (1.45 µM), isoniazid hydrogen chloride (**3**) (1.73 µM), octenidine dihydrochloride (**4**) (0.29 µM), ampicillin sodium salt (**5**) (0.89 µM) or cisplatin (**6**) (16.6 µM). Each co-formulated system was studied where the: i) SSA was pre-incubated with the *E. coli* for 10 mins before the antimicrobial agent was added (green); ii) antimicrobial agent was pre-incubated with the *E. coli* for 10 mins before the SSA was added (orange); iii) SSA and antimicrobial agent was added in co-formulation without any prior incubation (blue). The concentration of **2**-**6** alone was found to impede bacterial growth by < 30 % over 1100 mins.

References

1 G. M. Sheldrick, *Acta Crystallogr. Sect. A Found. Crystallogr.*, 2015, **71**, 3–8.
2 G. M. Sheldrick, *Acta Crystallogr. Sect. C Struct. Chem.*, 2015, **71**, 3–8.
3 O. V. Dolomanov, L. J. Bourhis, R. J. Gildea, J. A. K. Howard and H. Puschmann, *J. Appl. Crystallogr.*, 2009, **42**, 339–341.
4 J. R. Hiscock, G. P. Bustone, B. Wilson, K. E. Belsey and L. R. Blackholly, *Soft Matter*, 2016, **12**, 4221–4228.
5 L. J. White, S. N. Tyuleva, B. Wilson, H. J. Shepherd, K. K. L. Ng, S. J. Holder, E. R. Clark and J. R. Hiscock*, Chem. - A Eur. J.*, 2018, **24**, 7761–7773.
